# Haplotype-resolved reference genomes of the sea turtle clade unveil ultra-syntenic genomes with hotspots of divergence

**DOI:** 10.1093/gigascience/giaf105

**Published:** 2025-09-18

**Authors:** Larissa S Arantes, Tom Brown, Diego De Panis, Scott D Whiting, Erina J Young, Erin L LaCasella, Gabriella A Carvajal, Adam Kennedy, Deana Edmunds, Blair P Bentley, Jennifer Balacco, Conor Whelan, Nivesh Jain, Tatiana Tilley, Brian O'Toole, Patrick Traore, Erich D Jarvis, Oliver Berry, Peter H Dutton, Lisa M Komoroske, Camila J Mazzoni

**Affiliations:** Department of Evolutionary Genetics, Leibniz Institute for Zoo- and Wildlife Research (IZW), 10315 Berlin, Germany; Berlin Center for Genomics in Biodiversity Research (BeGenDiv), 14195 Berlin, Germany; Department of Evolutionary Genetics, Leibniz Institute for Zoo- and Wildlife Research (IZW), 10315 Berlin, Germany; Berlin Center for Genomics in Biodiversity Research (BeGenDiv), 14195 Berlin, Germany; Department of Evolutionary Genetics, Leibniz Institute for Zoo- and Wildlife Research (IZW), 10315 Berlin, Germany; Berlin Center for Genomics in Biodiversity Research (BeGenDiv), 14195 Berlin, Germany; Department of Biodiversity, Conservation and Attractions, Marine Science Program, Kensington, WA 6151, Australia; Conservation Medicine Program, Sc hool of Veterinary Medicine, Murdoch University, Murdoch, WA 6150, Australia; Marine Mammal and Turtle Division, Southwest Fisheries Science Center , National Marine Fisheries Service, National Oceanic and Atmospheric Administration, La Jolla, CA 92037, USA; Department of Biological Sciences, Florida Atlantic University, Boca Raton, FL 33431, USA; Rescue and Rehabilitation Department, New England Aquarium, Quincy, MA 02169 , USA; Animal Health Department, New England Aquarium, Quincy, MA 02169, USA; Department of Biological Sciences, Smith College, Northampton, MA 01060, USA; Vertebrate Genome Laboratory, The Rockefeller University, New York, NY 10065, USA; Vertebrate Genome Laboratory, The Rockefeller University, New York, NY 10065, USA; Vertebrate Genome Laboratory, The Rockefeller University, New York, NY 10065, USA; Vertebrate Genome Laboratory, The Rockefeller University, New York, NY 10065, USA; Vertebrate Genome Laboratory, The Rockefeller University, New York, NY 10065, USA; Vertebrate Genome Laboratory, The Rockefeller University, New York, NY 10065, USA; Vertebrate Genome Laboratory, The Rockefeller University, New York, NY 10065, USA; CSIRO Environomics Future Science Platform, Indian Ocean Marine Research Centre, Crawley, Western Australia 6009, Australia; Marine Mammal and Turtle Division, Southwest Fisheries Science Center , National Marine Fisheries Service, National Oceanic and Atmospheric Administration, La Jolla, CA 92037, USA; Department of Environmental Conservation, University of Massachusetts Amherst, Amherst, MA 01003, USA; Department of Evolutionary Genetics, Leibniz Institute for Zoo- and Wildlife Research (IZW), 10315 Berlin, Germany; Berlin Center for Genomics in Biodiversity Research (BeGenDiv), 14195 Berlin, Germany

**Keywords:** Cheloniidae, reference genomes, conservation genomics, adaptive evolution, genetic diversity, demography, synteny

## Abstract

**Background:**

Reference genomes for the entire sea turtle clade have the potential to reveal the genetic basis of traits driving the ecological and phenotypic diversity in these ancient and iconic marine species. Furthermore, these genomic resources can support conservation efforts and deepen our understanding of their unique evolution.

**Results:**

We present haplotype-resolved, chromosome-level reference genomes and high-quality gene annotations for 5 sea turtle species. This completes the catalog of reference genomes of the entire sea turtle clade when combined with our previously published reference genomes. Our analysis reveals remarkable genome synteny and collinearity across all species, despite the clade’s origin dating back more than 60 million years. Regions of high interspecific genetic distance and intraspecific genetic diversity are consistently clustered in genomic hotspots, which are enriched with genes coding for immune response proteins, olfactory receptors, zinc fingers, and G-protein-coupled receptors. These hotspot regions may offer insights into the genetic mechanisms driving phenotypic divergence among species and represent areas of significant adaptive potential. Ancient demographic analysis revealed a synchronous population expansion among sea turtle species during the Pleistocene, with varying magnitudes of demographic change, likely shaped by their diverse ecological adaptations and biogeographic contexts.

**Conclusions:**

Our work provides genomic resources for exploring genetic diversity, evolutionary adaptations, and demographic histories of sea turtles. We outline genomic regions with increased diversity, linked to immune response, sensory evolution, and adaptation to varying environments that have historically been subject to strong diversifying selection and likely will underpin sea turtles’ responses to future environmental change. These reference genomes can assist conservation by providing insights into the demographic and evolutionary processes that sustain and threaten these iconic species.

## Background

The rapid loss of biodiversity, driven by erosion and destruction of habitats globally, underscores the urgent need to develop strategies to mitigate this crisis and safeguard the planet’s ecological balance, reversing declines in biodiversity. One of the fastest growing technologies for understanding biodiversity and supporting its management is genomics [1]. Recent advances in high-quality genomic resources have facilitated our abilities to explore the genetic underpinnings of Earth’s biodiversity, enabling a deeper understanding of the evolutionary and functional complexities of life. Initiatives such as the Earth Biogenome Project [2], European Reference Genome Atlas [3], Darwin Tree of Life [4], and Vertebrate Genomes Project [5] have driven standards and recommendations for the production of high-quality reference genomes for conservation of biodiversity. These initiatives have resulted in an ever-growing database of high-quality reference genomes, which is expanding rapidly as technologies evolve.

This growth in genomic resources has allowed researchers to investigate the genetic bases of a number of features key to assisting in species management and conservation such as age and life span [6, 7], sex [8], abundance [9], and community composition [10], among others. Focusing on the evolutionary adaptations of iconic or umbrella species within ecosystems gives us the opportunity to efficiently monitor biodiversity and assess the health of these ecosystems [11, 12]. Anchoring such analyses to high-quality, chromosome-level reference genomes allows for a much more comprehensive interrogation of genomic architecture. This is largely due to the improved contiguity of these assemblies, which facilitates the resolution of complex genomic features such as multigenic regions potentially under selection, repeat-rich areas, and large-scale structural variants [13]. Transitioning from fragmented draft assemblies to highly contiguous genomes also enhances the detection of long runs of homozygosity (ROHs), offering critical insights into recent inbreeding and population history [14]. Finally, contiguous, accurate, chromosome-level assemblies, such as those presented here, allow us to investigate all of these features using one reference, which is not possible with fragmented or scaffold-level assemblies.

Sea turtles have existed since nonbird dinosaurs were roaming the Earth [15] and hold critical ecological roles in both oceanic and coastal environments, but they are at threat globally due to anthropogenic activities such as direct harvest, fisheries bycatch, habitat loss, and climate change, among other risks [16–18]. At present, 3 of the 7 extant sea turtle species have been classified under International Union for Conservation of Nature (IUCN) criteria as “endangered” (*Chelonia mydas* [19]) or “critically endangered” (*Eretmochelys imbricata* and *Lepidochelys kempii* [20, 21]) and a further 3 (*Lepidochelys olivacea, Caretta caretta*, and *Dermochelys coriacea* [22–24]) have been classified as “vulnerable.” Finally, while listed as “data deficient” under the IUCN Red List, *Natator depressus* [25] has been classified as “vulnerable” by the Australian government [26]. Extensive conservation efforts have led to positive outcomes for many populations [27], but effort and success have not been universal, with some populations still in decline [28].

Sea turtle species exist around the globe, inhabiting a remarkable diversity of ecological niches [29], spanning from deep cold-water oceanic divers like *D. coriacea* to range-restricted endemic species, such as *N. depressus* and *L. kempii* [30]. For other species, their habitats span the tropics and subtropics (*L. olivacea*) and broader, temperate, and tropical ranges, such as *C. mydas, E. imbricata*, and *C. caretta*. Some sea turtles demonstrate dietary specializations (*D. coriacea* and *E. imbricata*), while others (e.g., *C. mydas* and *C. caretta*) display generalist omnivorous feeding habits [31]. The genomic bases for these traits remain unclear, but having access to high-quality genomic resources would allow more fine-level investigation into genetic drivers behind the capabilities of sea turtles to live in varying habitats and adapt to changing conditions in the Anthropocene [32–34]. Annotated, high-quality reference genomes for each species allow for investigation into areas such as identifying genes under selection or areas of adaptive potential [13].

At present, genomes are available for 5 of the 7 extant sea turtle species, namely from *C. mydas* and *D. coriacea* [14], *C. caretta* [35], *E. imbricata* [36], and *L. olivacea* [37]. Previous analyses in particular of the genomes of *C. mydas* and *D. coriacea* that represent the 2 extant sea turtle families (*Dermochelyidae and Cheloniidae*) have revealed a high degree of synteny and collinearity, defined as blocks of the genome with shared arrangement and orientation of genomic features, such as genes or other aligned elements, within this ancient clade [14, 38]. Alongside this high level of apparent genomic conservation, small highly divergent genomic regions have also been observed between these 2 species, in particular in areas containing multicopy gene families such as the major histocompatibility complex (MHC) and olfactory receptors [14], as well as some rearrangement of genes potentially involved in temperature-dependent sex determination [38]. While these are clearly important genomic regions for understanding sea turtle adaptation and evolution, it is not clear if the differences between *C. mydas* and *D. coriacea* are species specific or how well they characterize comparative patterns within the entire sea turtle clade.

In this study, we add to our previous reference genomes for *C. mydas* and *D. coriacea* [14], by producing high-quality genomes for the remaining 5 extant sea turtle species. Our genomes are assembled using highly accurate PacBio HiFi (High-Fidelity) and Chromatin-Conformation-Capture (Hi-C) sequencing, producing genomes with chromosomes phased into both parental haplotypes. This first full catalog of sea turtle genomes now provides a unique opportunity to understand and investigate the evolution of sea turtles and contextualize their evolution among other turtles and tortoises, spanning hundreds of millions of years of evolution. We uncover high levels of genome-wide synteny across all Testudine genomes, with a notable pattern of genetic diversity and divergence within the sea turtle clade, intricately clustered within specific regions of particular chromosomes. These regions are enriched in immune-related genes, suggesting a role in the adaptive capabilities of these species. Furthermore, we performed demographic analysis, calculated genetic diversity, and identified ROHs in the genome to provide deeper insights for conservation efforts. Thus, we demonstrate the power of high-quality genomes to uncover complex patterns of genetic diversity and adaptation that are vital for understanding species evolution and guiding conservation strategies.

## Data Description

### Sequencing

For the 5 turtle species (*C. caretta* [NCBI Taxonomy ID: 8467], *E. imbricata* [27787], *L. olivacea* [27788], *L. kempii* [8472], *N. depressus* [27790]), we sequenced PacBio HiFi reads ranging from 35X to 60X coverage for each genome (Supplementary Fig. S1) and Hi-C sequences ranging from 47X to 129X coverage. We sequenced optical maps with N50 values ranging from 222 to 266 kbp and total DNA yields from 103 to 498 Gbp for long-range molecules of higher quality for 3 of the 5 species (*L. olivacea, C. caretta*, and *E. imbricata*; Supplementary Table S1). These datasets are available via the European Nucleotide Archive (ENA) and National Center for Biotechnology Information (NCBI; see Data Availability).

### Genome assembly

**Figure 1:**
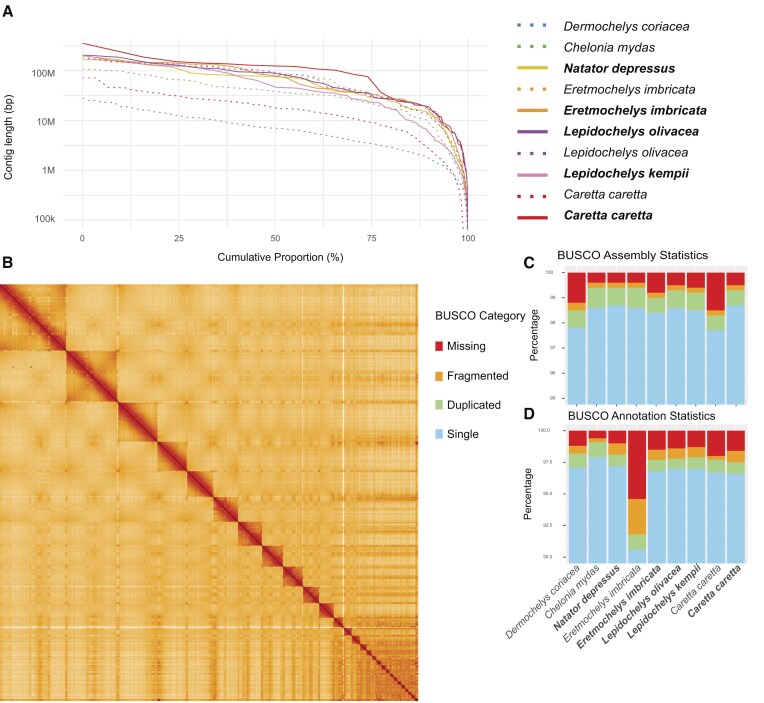
(A) Lengths of the assembled contigs for each species sorted by length (y-axis) and scaled to total length of each genome (x-axis). Genomes from this study are shown as complete lines with names in bold; previously published assemblies are shown as dashed lines. (B) Three-dimensional conformational arrangement of one assembled *Caretta caretta* haplotype genome assembly as evaluated by Hi-C. The x- and y-axes show the coordinates of the respective genome, and each detected contact in the genome is colored with increasing intensity from white to red. The red diagonal shows the self-interactions of each position with itself and close vicinity; squares show the high self-interaction of chromosomes. (C) Percentage detected single-copy orthologs identified in the genome assemblies calculated via BUSCO using the Sauropsida odb10 database. Scores are calculated based on detected mappings of ortholog sequences using the Miniprot mapper. (D) Gene completeness of sea turtle protein-coding annotations based on BUSCO genes identified in each annotated protein set.

Our haplotype-separated chromosome-scale assemblies are highly contiguous and, in particular, are significantly more contiguous than the previously published *D. coriacea* assembly based on PacBio continuous long read (CLR) data and *C. caretta* assembly based on Oxford Nanopore Technologies (ONT) reads (Fig. 1A, Supplementary Figs. S1 & S2, Supplementary Table S2). Moreover, the new haplotype assemblies show exceptional base accuracy, with a quality value (QV) ranging from 65.2 to 70.4 (Supplementary Table S2). For comparison, the older CLR-based primary assemblies display QVs in the range of 38.7 (*D. coriacea*) to 47.6 (*C. mydas*), while the ONT-based *C. caretta* assembly is much lower (in part due to a different sample used for analysis, Supplementary Table S2). All genomes were scaffolded into complete chromosome molecules, with between 99.1% and 99.9% of the assembled sequences assigned to the 28 chromosomes (Fig. 1B, Supplementary Figs. S3 & S4, Supplementary Table S2). The assembled genomes also show high gene completeness, with between 98.5% and 99.5% single-copy orthologs from the Sauropsida lineage identified by BUSCO (Fig. 1C, Supplementary Fig. S5, Supplementary Table S2).

### Genome annotation

Using a combination of approaches based on transcriptomic data, protein sequences from *C. mydas* and *D. coriacea*, liftover annotations from *C. mydas* and *Malaclemys terrapin pileata*, and *de novo* gene predictions, we created a set of protein-coding gene predictions for each of our assembled genomes (Supplementary Table S3). The annotations themselves are highly complete when evaluated based on single-copy orthologs from Sauropsida via BUSCO (Fig. 1D) and hierarchical orthology groups from Archelosauria via OMArk (Supplementary Fig. S6), reaching comparable completeness to previous annotations generated by RefSeq, with BUSCO scores between 97.2% and 98.1% and OMArk completeness scores between 97.46% and 98.47%, furthermore capturing many BUSCO genes missing in the annotation provided for the existing *E. imbricata* reference genome [36].

## Analyses

### Genome synteny

**Figure 2:**
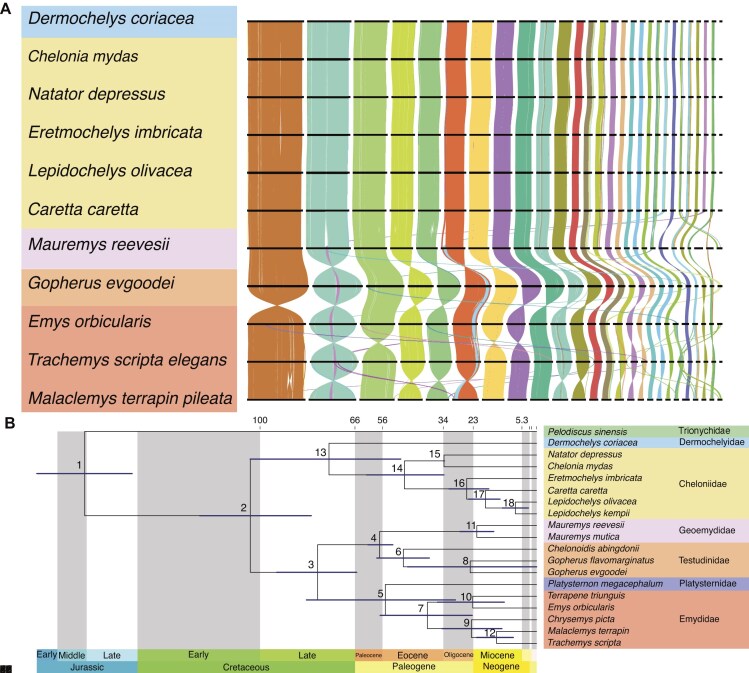
(A) Genome-wide gene-synteny plots across Testudines. Each line represents a reciprocal best-hit protein match between annotated genes in each consecutive genome. Lines are colored based on colocalization across all 11 genomes determined by Fisher’s exact test. Chromosomes are ordered based on synteny to *Dermochelys coriacea*. (B) Divergence times of species within the suborder Cryptodira based on protein-coding genome annotations. The bars on each node represent the 95% highest posterior density (HPD) intervals for node age estimates. Divergence times and confidence intervals for each numbered node are detailed in Supplementary Table S4.

Based on identification of orthologous genes and their locations in Testudine genomes, we uncovered remarkably high synteny across the clade, encompassing over 100 million years of evolution with only a small number of hotspots of variation identified among the unique-sequence regions of the genomes. Particularly among the sea turtles, all 28 chromosomes were highly collinear and syntenic (Fig. 2A & Supplementary Fig. S7) with complete one-to-one synteny demonstrated at the chromosome level, with the exception of one region at the end of chromosome 14 in *D. coriacea*, found in chromosome 11 in the 6 *Cheloniidae* turtle species (Supplementary Fig. S7).

Across Testudine genomes (i.e., including terrestrial and freshwater turtle and tortoise families; Fig. 2), we found that the macrochromosomes (>50 Mb in length) exhibited high synteny across all turtle genomes and among the microchromosomes (<50 Mb in length), with only chromosomes 21 and 26 from sea turtles rearranged in other turtle genomes. In these instances, chromosomes 21 and 26 from the sea turtle genomes were found in the arm of chromosome 4 (which is syntenic to chromosome 6 in the sea turtle genomes) and the central region of chromosome 2, respectively, in the genome of the Chinese pond turtle (*Mauremys reevesii*), with this positioning conserved across all other Testudine genomes (Fig. 2A, Supplementary Figs. S8, S12, & S13).

### Phylogenomic analysis

Phylogenetic analysis using protein-coding sequences for all turtle species with annotation available provided insights into evolutionary relationships and speciation events within suborder Cryptodira, which includes most living turtles and tortoises (Fig. 2B). The topology and divergence time support the findings of previous studies based on a few nuclear markers or mitochondrial DNA [39–41] (Supplementary Table S4). Our genome-wide analysis indicates that the sea turtle clade diverged from other Durocryptodira species 104 million years ago (mya) (95% highest posterior density [HPD] = 81.9 to 122 mya). Dermochelyidae (including *D. coriacea*) separated from the Cheloniidae family approximately 75.4 mya (95% HPD = 49.4, 104). Within the Cheloniidae family, the divergence of *C. mydas* and *N. depressus* occurred approximately 33.6 mya (95% HPD = 33.5, 33.8), while the other species diverged around 25.4 mya (95% HPD = 17.4, 31.9). *Lepidochelys kempii* and *L. olivacea* were the most recently diverged lineages, having split around 7.72 mya (95% HPD = 2.99, 12.4), a time period associated with significant environmental changes such as the closure of the Tethys Sea and cooling of the southern oceans, which likely disrupted gene flow and contributed to the speciation of these 2 *Lepidochelys* species [40, 42]. We acknowledge that the MCMCTree method used to estimate divergence times assumes a strictly bifurcating tree and does not account for postdivergence gene flow. This limitation is particularly relevant given the well-documented history of hybridization among sea turtle species [43], which can lead to underestimated divergence times when gene flow occurs after initial lineage splitting.

### Genome-wide patterns of diversity and divergence

**Figure 3:**
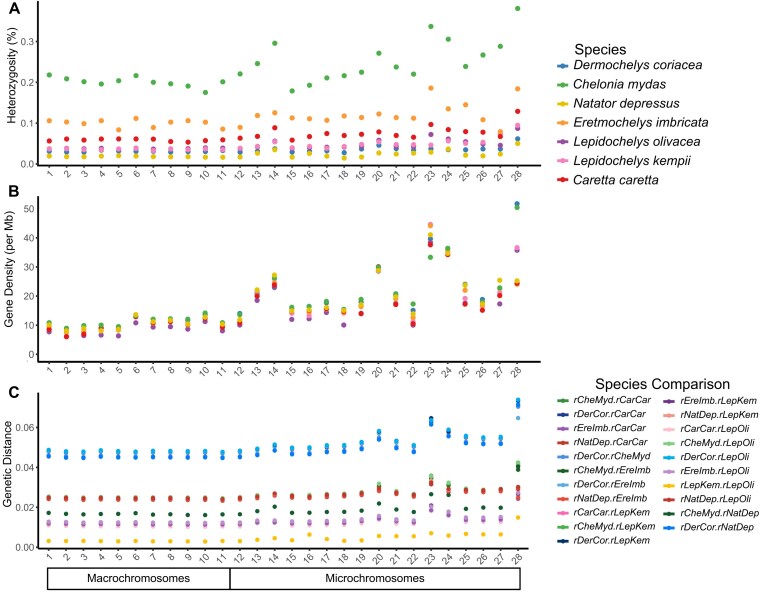
(A) Heterozygosity, (B) gene density (per Mb), and (C) pairwise genetic distance per chromosome for the 7 sea turtle reference genomes. Chromosomes longer or shorter than 50 Mb are highlighted as macrochromosomes or microchromosomes, respectively.

Comparisons of within-individual genetic diversity, measured by average heterozygosity per chromosome, revealed consistent variation across chromosomes in the individuals sequenced, with each species showing distinct magnitude of variation (Fig. 3A & Supplementary Fig. S9). Notably, chromosomes with high intraspecific diversity also exhibited higher gene density (Fig. 3B) and increased interspecific genetic distance (Fig. 3C). Consistent with findings in sea turtles and other species with microchromosomes [44], we found that the average heterozygosity, as well as the gene density and interspecific genetic distance, was higher for microchromosomes (12–28) than for macrochromosomes (1–11) (*P* < 0.05) (Fig. 3A–C). In particular, chromosomes 13, 14, 20, 23, 24, and 28 exhibited heightened genetic diversity, interspecific divergence, and gene density (Fig. 3A, C, Supplementary Figs. S9 & S10, Supplementary Table S5).

**Figure 4:**
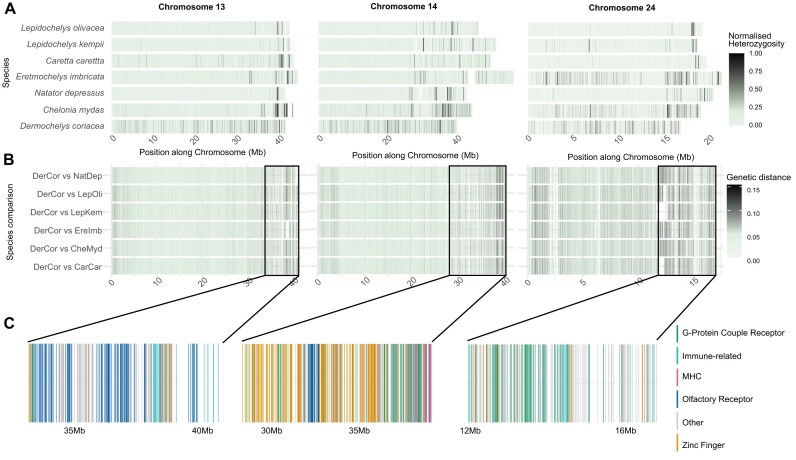
Genetic diversity and divergence hotspots contain genes associated with immune response, olfactory receptors, zinc fingers, and G-protein-coupled receptors. (A) The heatmap illustrates normalized heterozygosity (He) across chromosomes 13, 14, and 24 for 7 sea turtle species, displaying He values in nonoverlapping 50-kb windows. The normalization highlights chromosomal hotspots rather than overall diversity. (B) Pairwise genetic distances between the 6 sea turtle species and *D. coriacea* are shown along the same 3 chromosomes. Genetic distance was calculated as the ratio of interspecific single variants per 10 kb. Black boxes highlight the chromosome areas of increased genetic distance among sea turtle genomes. (C) Multicopy gene families located in the highlighted regions are displayed and color-coded by their annotation. Chromosome coordinates are shown by their position in the genome of *D. coriacea*.

Furthermore, increased levels of heterozygosity and interspecific genetic distance were concentrated at particular hotspot regions, defined as regions with heterozygosity exceeding 4 times the chromosomal mean and genetic distance double that of the chromosomal mean, rather than being uniformly distributed across an entire chromosome (Supplementary Fig. S9). Thus, we identified the 3 regions located in chromosomes 13, 14, and 24 exhibiting colocalized elevations in heterozygosity and genetic distance across sea turtles (Fig. 4). While we identified these hotspot regions by calculating genetic distances from all species in relation to *D. coriacea* (Fig. 4B), this pattern is consistent across pairwise comparisons between all species (Supplementary Figs. S10 & S11).

Following functional annotation of the genes found in these hotspots, we found enrichment for multicopy gene families coding for proteins with functions in immune response, olfactory receptors (ORs), zinc fingers, and G-protein-coupled receptors (GPCRs) (Fig. 4C, Tables S6 & S7). This included enrichment of immunology-related genes, GPCRs, ORs, and zinc-finger genes in chromosome 13 (adjusted *P* < 10^−42^, 10^−47^, 10^−79^, 10^−2^ll, respectively); MHC genes, immunology-related genes, GPCRs, ORs, and zinc-finger genes in chromosome 14 (adjusted *P* < 10^−24^, 10^−6^, 10^−2^, 10^−9^, 10^−52^, respectively); and immunology-related genes and GPCRs in chromosome 24 (adjusted *P* < 10^−3^ and 10^−3^, respectively). A particular concentration of olfactory receptors—known for their role in odor perception and detection of chemical cues—was identified in the hotspot region of chromosome 13 (Fig. 4), and MHC genes were concentrated within the identified hotspot on chromosome 14.

### Homozygosity patterns and historical demography

**Figure 5:**
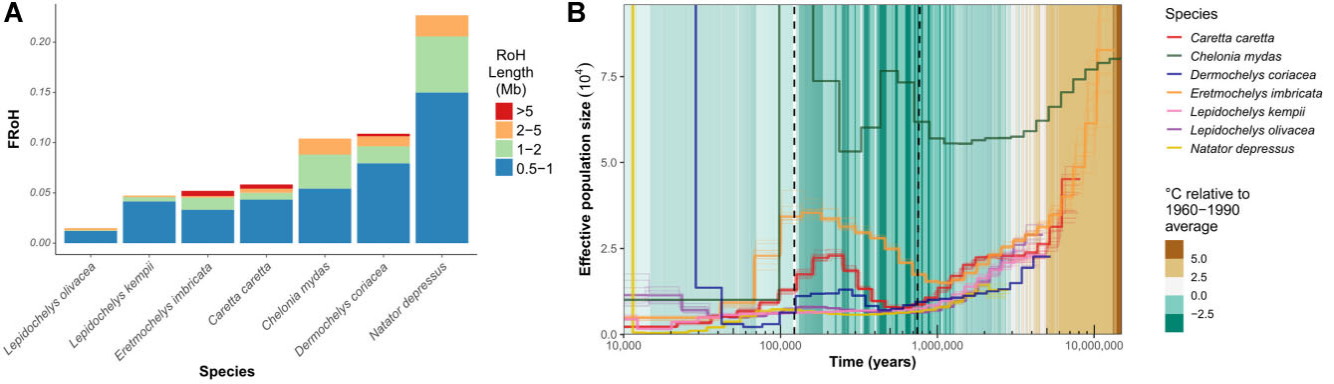
(A) Inbreeding levels for the 7 sea turtle individuals, measured as the proportion of the genome in runs of homozygosity (FROH). FROH are categorized by length (in Mb), where longer runs indicate more recent events associated with a shared common ancestor of the individual’s maternal and paternal lineages, while shorter runs suggest older inbreeding events. (B) Ancient demographic history for the 7 sea turtle species reconstructed with pairwise sequentially Markovian coalescent (PSMC) plot. Dashed lines indicate the Last Interglacial (Eemian Period, 130,000 to 115,000 years ago) and mid-Pleistocene transition (1.2–0.5 million years ago). Bootstrap replicates (10 for each lineage) are plotted in lighter lines. Inferred fluctuations in effective population size (N_e_) were rescaled assuming 30-year generation time and 1.2 × 10^−8^ per generation mutation rate.

We analyzed the proportion of the genome in ROHs (FROH) for each species and categorized segments by length to distinguish between ancient demographic events that resulted in background relatedness (short ROH) and recent consanguinity (long ROH) [45]. The *N. depressus* individual had the highest overall FROH (0.227), predominantly comprising short (0.5–1 Mb) segments but with substantial representation in longer categories (1–2 and 2–5 Mb; Fig. 5A). This distribution suggests its elevated homozygosity results from a combination of ancient demographic processes and more recent population declines. In contrast, the *L. olivacea* individual showed the lowest FROH (0.0149), consisting mainly of short ROH, indicating a historically larger and more stable population. The reference *C. mydas* individual, despite moderate total FROH, showed a higher proportion of long ROH segments (Fig. 5A, Supplementary Fig. S9). This pattern is consistent with Bentley et al. [14], as this individual originates from a small breeding population in the Mediterranean Sea, where recent shared ancestry between maternal and paternal lineages is more likely [45].

Using pairwise sequentially Markovian coalescent (PSMC) models [46], we reconstructed the demographic histories of the 7 extant sea turtle species, revealing consistent patterns of population declines beginning approximately 1–9 mya, likely driven by cooler sea temperatures (Fig. 5B). During the Mid-Pleistocene Transition, between 500 kya and 1.2 mya, population decline ceased, and all species, except *L. kempii*, began to experience synchronous population expansions, with the growth particularly pronounced in *C. mydas, C. caretta*, and *E. imbricata*, while *L. kempii* maintained a relatively stable population size. The population peak occurred between 300 kya and the Last Interglacial in the Eemian period (130–115 kya). This period of growth was followed by a second population decline across all species, starting roughly 100 kya until recently around 50 kya. The 3 species with relatively stable historical population sizes—*N. depressus, L. kempii*, and *L. olivacea*—differ significantly in their levels of genetic diversity. *N. depressus* exhibits the lowest heterozygosity, while the 2 *Lepidochelys* species display relatively high heterozygosity. These results were found to be robust when considering only the largest 11 chromosomes (macrochromosomes, Supplementary Fig. S14), removing their regions with elevated levels of heterozygosity and genetic diversity (Fig. 3, Supplementary Table S9) to minimize potential confounding effects of selection. Demographic trajectories were further inferred using an independent method, MSMC2, which produced broadly consistent demographic trajectories and effective population sizes as those obtained via PSMC (Supplementary Fig. S15).

## Discussion

Our chromosome-scale, annotated genomes from the sea turtle clade revealed remarkable genetic synteny across this slowly evolving group of animals, while also revealing hotspot regions of the genome consistently undergoing accelerated evolution and divergence that likely play important roles in the morphological and ecological diversity exhibited among these species. These regions contained genes important for immune responses, the ability to sense and respond to the environment and regulate gene expression under fluctuating environmental conditions [47]. This builds on previous results comparing genomes of *C. mydas* and *D. coriacea* [14], demonstrating that sea turtle genomes have remained highly syntenic since their split from freshwater turtles and tortoises around 100 million years ago.

Our findings of ultra-synteny across the high-quality genomes of all 7 extant sea turtle species reveals a striking conservation of chromosomal architecture that may underlie the known hybridization among sea turtles observed between ancient [43] and recent [48] species divergence. This structural stability may have supported ancient hybridization events via preserved gene order and structure, facilitating chromosome pairing during meiosis, reducing incompatibilities, and enabling the formation of viable and fertile hybrids [49].

The availability of high-quality reference genomes for all sea turtles opens new avenues to explore fundamental questions about their adaptation, immunity, and sensory evolution. Sea turtles exhibit remarkable adaptations to marine environments, including extreme migratory behaviors [50], saltwater tolerance [51], natal homing [52, 53], and temperature-dependent sex determination [54], yet the genetic basis of these traits remains poorly understood. Our results highlight microchromosomes and specific regions of reduced relative synteny in macrochromosomes as key loci enriched in gene density and genetic variation across the sea turtle clade. This pattern is also observed in birds and other reptiles, with the high GC content and high recombination rate of the microchromosomes potentially playing a significant role in promoting diversification [44].

The highlighted hotspots of evolutionary diversification harbor multicopy gene families, such as olfactory receptors involved in detecting odorants and adapting to the chemical complexity of habitats [55, 56], as well as MHC genes, central to the immune response to diseases [57, 58]. These multicopy gene families found within divergent hotspots may represent adaptation mechanisms that maintain flexibility in response to dynamic or disruptive selective pressures, potentially aiding immune variability, environmental sensing, and essential survival responses across the diverse habitats these turtles inhabit. Sea turtles are known to inhabit a vast proportion of the globe’s seas, found in both deep and shallow waters [47, 48], migrating long distances across highly variable temperatures, currents, and salinity [37], as well as possessing an immune system highly influenced by these changing environments [49, 50]. As enhanced MHC variation is associated with lower disease susceptibility [59], the MHC gene copy numbers and heterozygosity in sea turtles have been previously proposed to vary among species based on their habitats, with those exposed to higher pathogen loads and diversity in neritic environments exhibiting greater MHC gene copy numbers than species inhabiting pelagic habitats, an area that would require manual validation in future studies [14]. Thus, these genome hotspots of increased diversity and divergence may hold the key to understanding chemosensory evolution, disease resistance, and phenotypic diversity in sea turtles. Further exploration of these regions could shed light on adaptive forces that have influenced the evolutionary trajectory of sea turtle species.

From a conservation perspective, genomic resources offer powerful tools to help understand sea turtle population viability and resilience to anthropogenic threats. Genomic diversity, inbreeding levels, effective population sizes, and demographic histories, are critical metrics for assessing extinction risk and adaptive potential [60]. Our results indicate that *N. depressus* has maintained a long-term low population size and genetic diversity, similar to the demographic trajectory observed for *D. coriacea* [14], rather than a sharp loss due to recent declines, highlighting the need to distinguish historical demographic patterns from contemporary inbreeding. While reduced diversity may have been sustainable in the past, potentially leading to some degree of purging of deleterious alleles, it could still limit adaptive capacity in the face of rapid environmental change [61]. In contrast, *Lepidochelys* species exhibit comparatively high genetic diversity despite their historically small population sizes, with *L. kempii* retaining higher genetic variation even with its restricted distribution in the Gulf of Mexico (Supplementary Table S5). This contrasts with the other range-restricted sea turtle species, *N. depressus*, suggesting that endemism alone does not consistently predict genetic diversity in sea turtles. We acknowledge that our results are based on a single individual, and the individual’s origin should be considered, as previous studies have highlighted different demographic histories and genetic diversity between ocean basins [62]. However, we have previously shown demographic histories of *C. mydas* and *D. coriacea* to be consistent even from individuals in different populations [14]. In the case of range-restricted species such as *N. depressus* and *L. kempii*, we anticipate that the demographic histories likely reflect range-wide patterns.

The demographic histories of sea turtle species reveal broadly similar trajectories of expanding and contracting effective population size over the past 10 million years, though with the magnitude of N_e_ varying between the species. These unique patterns likely reflect the intersections of species-specific life histories and changing environments such as fluctuations in ocean temperature, sea level, and connectivity [63, 64]. Species inhabiting shallow coastal habitats, such as *N. depressus*, were likely particularly affected by the dynamic coastal environment [65]. Specifically, the low and stable population size of *N. depressus* may reflect its restricted neritic distribution and tendency to disperse over smaller distances compared to other sea turtle species, potentially limiting foraging opportunities, preventing the species from achieving the global distribution exhibited with some other turtles [66]. On the other hand, the historically low population size of *D. coriacea* may be attributed to its specialized open-ocean cold-water lifestyle and high trophic position, primarily consuming gelatinous zooplankton, along with behavioral constraints tied to its large size and the challenges of terrestrial nesting [29]. The early pleistocene glaciation appears to have impacted dermochelyids more severely than the chelonids, resulting in the extinction of all but one of the dermochelyid species [64], which subsequently entered the Pleistocene expansion as a severely bottlenecked remnant population [67]. Conversely, *E. imbricata, C. caretta*, and *C. mydas* fared better during the glacial contraction [68] and experienced more pronounced population expansions during the Pleistocene. The ability of *E. imbricata* to exploit diverse habitats and food sources, with a diet centered on coral reef organisms, likely favored its population expansion by reducing interspecies competition [69]. Similarly, *C. caretta* and *C. mydas* may have benefited from their broad dietary flexibility and ability to thrive in diverse temperate and tropical environments [29].

We observed a strong synchrony in population expansions, with population peaks between 300,000 years ago and the Eemian period (130,000–115,000 years ago), although the magnitude of demographic changes varied among lineages. Reid et al. [62] also reported a synchronized demographic response after the Last Glacial Maximum across most sea turtle lineages. These population expansions most likely helped maintain genetic diversity in these species. Expanding these comparisons to include individuals from additional populations might further corroborate the links between demographic history and ecological factors such as habitat specificity, feeding habits, thermal preference, developmental and adult foraging stages (oceanic vs. neritic), and environmental conditions. This will be particularly valuable for estimating recent changes in population size, which rely on population-level genomic resources [70], and for understanding their connection to human-mediated environmental disturbances.

## Potential Implications

High-quality reference genomes are important building blocks for creating genomic toolkits for species conservation and management. One exciting consequence of discovering the levels of genome-wide synteny exhibited between sea turtles is that genetic markers identified for determining features such as sex and adaptive traits in one species may also be directly applicable to other species without the need for new rounds of research and development. Having complete, annotated, chromosome-level genomes for all sea turtles means that such markers or genetic regions can be quickly verified between the species and turned into practical conservation toolkits. While they may not be required for individual studies with a scope of a single or few populations, they are critical for anchoring markers and comparing across studies and species. For example, the advancement from mitochondrial to whole-genome markers helps alleviate conflicting signals that can arise from nuclear integrations of mitochondrial sequences (NuMTs), recently misinterpreted as evidence of a new species of *D. coriacea* [71, 72], giving better resolution to future genomic studies with potential conservation implications. In this case, the identified NuMT contained a portion of the mtDNA Control Region, commonly used for population structure analysis in all the sea turtle species, but we did not find such NuMTs in the other genomes reported here.

We believe these reference genomes will also be valuable for measuring and predicting the impact of climate change on sea turtles. For ectotherms like reptiles, climate impacts may be particularly pronounced due to their sensitivity to thermal fluctuations [73]. For sea turtles, these effects have potential to be even greater due to their temperature-dependent sex determination, where changes to nest temperature can disrupt sex ratios and reproductive success [54, 74]. As these effects may be best evidenced via the epigenome, having access to complete, annotated reference genomes increases the predictive power of markers based on measuring levels of DNA or chromatin modifications.

Our findings highlight how different sea turtle species have responded to ancient climate changes, reflecting a range of adaptive strategies and unique biogeographic scenarios. Understanding how species have historically responded to changes in climate offers insights into their potential reactions to current and future anthropogenic disturbances, helping to inform conservation strategies and predict the long-term impacts of climate shifts on sea turtle populations.

## Methods

### Sampling

Whole blood samples were collected from juvenile *C. caretta, L. kempii, L. olivacea*, and *E. imbricata* and immediately flash-frozen at −80°C. A blood sample from a female *N. depressus* was collected as described in Young et al. [75] and subsequently stored in ice for 24 hours before being frozen at −80°C. Additionally, organ tissue samples were collected opportunistically from *L. kempii* (brain, kidney, and ovary) and *C. caretta* (thymus, ovary, brain, liver, heart, spleen, testes, kidney, and lung) and flash frozen at −80°C for long- and short-read transcriptomic sequencing for genome annotation. We shipped the samples on dry ice or in a liquid nitrogen dry shipper, ensuring that they remained consistently frozen throughout transit.

The sampled *C. caretta* and *L. olivacea* individuals were originally stranded on the coast of Oregon, USA, in 2021 and 2022 at 44.9426 N, 124.024 W and 44.5455 N, 124.0751 W, respectively, and are part of the loggerhead North Pacific regional management unit (RMU), including nesting beaches in Japan and foraging and migration through the North Pacific, and the olive ridley East Pacific RMU, including nesting beaches in Mexico and North America and foraging and migration throughout the Pacific, respectively. The sampled *E. imbricata* individual was stranded in Hawaii, USA (20.0334 N, 155.8264 W), and belongs to the relatively small population part of the hawksbill North Central Pacific RMU [76]. The sampled *N. depressus* individual comes from a summer nesting population close to the center of the range and from within the largest and most genetically diverse Western and Northern Australian stocks (https://www.wamsi.org.au/kmrp/kimberley-marine-research-node-projects). The *L. kempii* individual was sampled at the New England Aquarium as a rehabilitated cold-stun animal from Cape Cod Bay, MA, USA, belonging to the Northwest Atlantic RMU, which constitutes the single population of this species.

### Sample processing and sequencing

We extracted and purified DNA using a Bionano SP DNA kit (PN 80042) for *C. caretta, E. imbricata*, and *L. kempii*. We used a MagAttract HMW DNA Kit (Qiagen 67563) for *N. depressus* and *L. kempii*. We measured DNA quantity using triplicate measures and Qubit 3 fluorometer (Invitrogen Qubit dsDNA Broad Range Assay cat no. Q32850) and measured DNA size with an Agilent Femto Pulse. We fragmented the DNA to 15–20 kb length prior to library preparation using a Megaruptor 3 (Diagenode) and standard hydropores (cat. E07010003).

We prepared the PacBio HiFi libraries using a SMRTbell prep kit 3.0 (Pacific Biosciences PN 102–182-700) and PacBio barcoded primers. We size-selected the libraries to remove DNA under 10 kb using a Pippin HT instrument (Sage Science). We then quantified the size-selected HiFi libraries with a Qubit 3 Fluorometer (Qubit dsDNA HS Assay Kit) and assessed the average size with an Agilent Femto Pulse.

For *C. caretta, E. imbricata*, and *L. kempii*, we sequenced HiFi libraries with a PacBio Sequel IIe instrument on 8 M sequencing molecule, real-time (SMRT) cells (101–389-001) using the Binding kit 3.2 (102–333-300) and Sequel II sequencing kit 2.0 (101–820-200), along with a 40-hour movie time with a 2-hour preextension. For *N. depressus* and *L. olivacea*, we sequenced HiFi libraries with a PacBio Revio instrument using a Revio polymerase kit (102–817-600), Revio sequencing plate (102–587-400), and 24-hour movie with a 1.6-hour preextension.

For *C. caretta, E. imbricata, L. kempii*, and *L. olivacea*, we prepared Omni-C libraries using the Dovetail Omni-C Kit (Dovetail Genomics) according to the manufacturer’s protocol. We then sequenced the Omni-C libraries with the Illumina NovaSeq 6000 platform with a 2 × 150-bp read length. For *N. depressus*, we prepared the Hi-C library using the Arima-HiC 2.0 kit (Arima Genomics) following the manufacturer’s protocol. We then sequenced the Hi-C libraries with the Illumina NovaSeq 6000 platform with a 2 × 150-bp read length.

For Bionano optical mapping, we labeled 750 ng DNA using direct labeling enzyme (DLE1) and the Bionano Prep Direct Label and Stain (DLS) protocol (document number 30206) and then imaged the DNA on the Bionano Saphyr instrument.

To prepare RNA for sequencing, we extracted and purified total RNA using a QIAGEN RNAeasy kit (cat. 74104). We determined the RNA quantity using a Qubit 3 fluorometer (Invitrogen Qubit RNA High Sensitivity [HS] Kit, cat. Q32852) and measured the RNA integrity (RIN) score using an Agilent Fragment Analyzer. We prepared the RNA-seq libraries using the Illumina Stranded mRNA Prep kit and sequenced the libraries with the Illumina NovaSeq 6000 platform with a 2 × 100-bp read length. We generated Iso-seq cDNA libraries using the NEBNext Single Cell/Low Input cDNA Synthesis & Amplification Module in combination with PacBio’s SMRTbell Prep Kit 3.0. We then sequenced the Iso-seq libraries on a PacBio Sequel IIe machine using a Sequel II 8 M SMRTcell.

### Genome assembly

We performed the assemblies of each genome following the best practices established by the Vertebrate Genomes Project [5, 77]. In particular, we trimmed the raw sequencing reads for adapters using cutadapt v4.9 to remove any remaining PacBio adapter sequences from the PacBio HiFi reads and Illumina adapters from the Hi-C reads. We assembled initial contig sets for each species using hifiasm [78], v0.19.4–9, l2-l3, Hi-C phasing mode, using both PacBio HiFi and Illumina Hi-C reads as input to generate 2 haplotype-phased sets of contigs. We then removed retained haplotigs from each assembly with purge-dups [79] v1.2.6, -e. To scaffold the assembled contigs into chromosomes, we used the hybrid-scaffold tool from the Bionano Solve suite (v3.7.0, VGP mode) to scaffold with optical maps and then mapped the Hi-C reads to the set of initial scaffolds using bwa-mem [80] v2.2.1, -5SP -T0, and scaffolded into pseudo-chromosomal units using yahs [81] v1.2a.1. Finally, we performed rounds of manual curation following the Sanger rapid-curation pipeline [82], joining any missed scaffolds and removing any false joins in the assembly. We screened for any retained adapter or vector sequences using NCBI’s FCS-adapter and for foreign contaminant sequences using NCBI’s FCS-GX [83] v0.5.4.

### Genome annotation

To generate a set of protein-coding annotations for each genome, we incorporated evidence from transcript data, protein sequences, *ab initio* machine learning approaches, and homology to genomes of related species. To create *ab initio* predictions, we ran Helixer [84] vv0.3.3_cuda_11.8.0 using argument *–lineage vertebrate*. To generate protein-based gene model predictions, we mapped protein sequences from existing *C. mydas* and *D. coriacea* assemblies (GCF_015237465.2 and GCF_009764565.3, respectively) using miniprot [85] v0.13-r248. To create transcript-based gene model predictions, we mapped paired-end RNA-seq data to each genome using hisat2 [86] v2.2.1 using argument *–dta* and filtered the alignments using samtools [87] v1.19.2 with argument *-F 3840*. We then generated a *de novo* transcript assembly using stringtie [88] v2.2.1 and predicted coding sequences using TransDecoder [89] v5.7.1 and included only those gene models with a TransDecoder score greater than 20. Similarly, we mapped PacBio Iso-seq data to the genome using minimap2 [90] v2.28-r1209 with argument *-x splice:hq* and filtered the alignments using samtools with argument *-F 3840* and built gene models using stringtie with argument *-L* and predicted CDS using TransDecoder as above. To generate homology-based predictions, we created lastz-alignment chains from *C. mydas* and *Malaclemys terrapin pileata* genomes (GCF_015237465.2 and GCF_027887155.1) using the *make_lastz_chains* (v2.0.8) tools from TOGA [91], and we generated the set of homology gene predictions using TOGA (v1.1.6).

To generate a set of best gene models, we used EvidenceModeler [92] v2.1.0 to combine all of the above evidences using the weights defined in Supplementary Table S8.

To create functional annotations, we mapped the amino acid sequences from each gene model against the swissprot database [93], release 2023_03, using the diamond [94] v2.1.8 blastp search, and we identified Pfam, PROSITE, and SUPERFAMILY homology using Interproscan [95, 96] v5.59–91.0. Finally, we filtered gene models that had no identified swissprot or Pfam homology and were over 50% masked or missing start and/or stop codons.

### Genome synteny

To determine the number and sizes of syntenic regions within turtle and tortoise genomes, we made use of the annotated protein sequences to find orthologous genes within the genomes and uncover regions of local syntenic inheritance. By identifying synteny based on orthologous protein sequences, we relied on the unique elements of the genome, ignoring repetitive or other noncoding areas of the genome. We used Oxford Dot Plot [97] v0.3.3 to identify orthologous genes and plot synteny via ribbon plots, particularly the https://github.com/conchoecia/odp/blob/main/scripts/odp_nway_rbh pipeline. We extracted protein sequences from the annotated chromosomes of each Testudine assembly using the AGAT [98] v1.0.0 command *agat_sp_extract_sequences.pl* and mapped the sequences against each other using the diamond [94] v2.1.9 blastp command with an e-value cutoff of 1e-5. Syntenic protein alignments were only included in the next step if the same hit was found to be the best for each pairwise comparison (reciprocal best hits). To determine syntenic blocks, permutation tests were performed with 10,000 bootstraps, and only those syntenic blocks with a falser discovery rate of less than 0.05 were included and plotted as distinct colors in the ribbon diagrams. We performed this analysis once using the sea turtle genomes as input and once with 1 species per genus for all currently available chromosome-scale reference genomes with annotations on GenBank alongside those from this study (GCF_016161935.1, GCF_007399415.2, GCF_028017835.1, GCF_013100865.1, GCF_027887155.1, GCF_009764565.3, and GCF_015237465.2).

### Phylogenetic analysis

To reconstruct the phylogenetic history of the Testudine clade, we built a tree based on the protein sequences of all reference genomes submitted to GenBank with a protein-coding annotation. For each genome, we reduced the gff files to contain only the longest isoform per gene using the AGAT [98] v1.0.0 command *agat_sp_keep_longest_isoform.pl* and then extracted the protein sequences for each gene using the command *agat_sp_extract_sequences.pl*. To find the single-copy orthologs, we used OrthoFinder [99] v2.5.5.2 using the amino acid files as input. We then aligned the single-copy orthologs using MAFFT [100] v7.475, trimmed the resulting multialignment files using trimAL [101] v1.4.1 with argument -automated1, concatenated the trimmed alignments into a supermatrix containing all aligned sequences, and constructed a phylogenetic tree using IQtree [102] v2.2.5 with 1,000 bootstraps (-B 1000). To further estimate the branching points in the tree, we took upper- and lower-bound divergence time estimates from timetree.org for all internal nodes and used these as calibration times for MCMCTree [103] paml v4.10.7 using the JC69 model. A full list of commands can be found in the script “create_tree.sh.”

### Genome-wide diversity and divergence

Aiming to explore the genome-wide patterns of genetic diversity in the sea turtle clade, we performed the single-nucleotide polymorphism (SNP) calling for the 7 sea turtle species using the jATG pipeline [104]. First, we mapped PacBio HiFi reads for the 5 genomes generated in this work against their own reference genome using minimap2 [90], v2.26 and mapped Illumina 10x reads for *D. coriacea* and *C. mydas* using bwa-mem2 [105] v2.2.1. Following the mapping, we removed PCR duplicates from BAM files using MarkDuplicates from GATK [106] v4.6. We performed variant calling using GATK v4.6 HaplotypeCaller and GenotypeGVCF. We then filtered the resulting genomic variant call format (gVCF) using BCFtools [107] following GATK’s recommended parameter thresholds [106], removing low mapping quality positions (MQ >30), as well as SNPs with a depth lower than 8 and greater than 2× the average coverage, and keeping only biallelic positions. We also filtered small scaffolds, keeping only the 28 chromosomes for the subsequent analysis. Additionally, we excluded SNPs located in masked regions from subsequent analyses, identified by masking the genome with Dfam TE Tools v1.85 using RepeatModeler [108] and RepeatMasker [109]. We converted all filtered genotypes to missing data, producing a base-pair resolution gVCF file.

This filtered gVCF was used for genome-wide heterozygosity assessment and ROH analysis using Darwindow [110]. This tool enables the visualization of heterozygosity and ROH along the scaffolds, providing a clear visual assessment of the accuracy of the ROH calls. We calculated heterozygosity based on a sliding-window approach with nonoverlapping windows of 50 kb, without applying a filter for missing data. We identified ROHs using a heterozygosity threshold calculated from the average genome-wide heterozygosity of each species. A window was considered to have low heterozygosity if its value fell below one-fifth of the mean heterozygosity. The minimum length of an ROH was set to 500 kb, composed of at least 10 adjacent windows of 50 kb. The maximum proportion of missing data per window was 0.7. The inbreeding level was calculated as the proportion of the genome marked as ROH (FROH).

We calculated gene density using a custom Python script (GeneDensityCalculation.py) that counts the number of genes per Mb across the genome. We estimated pairwise genetic distances using a window-based approach, leveraging genome alignments generated with Progressive Cactus [111] v2.9. The repeat-masked genomes were aligned to ensure that unique regions of the genome were correctly aligned, while repetitive regions were excluded from the mapped regions. We used the halSnps pipeline to identify interspecific single variants and the halAlignmentDepth pipeline to define 10-kb windows of aligned regions across the genome. We defined genetic distance in each window as the ratio of interspecific single variants per 10 kb. We then identified hotspots of genetic divergence, diversity, and gene density by screening these metrics along the chromosomes and targeting windows where heterozygosity was higher than 4 times the chromosome mean and genetic distance exceeded twice the chromosome mean.

### Demographic analysis

We inferred the demographic histories of the 5 sea turtle species whose genomes were assembled in this study using the PSMC model [46]. We first extracted the consensus sequence from the filtered gVCF files generated above with BCFtools, then converted the resulting consensus fasta file into the PSMC input format using fq2psmcfa. We ran PSMC using the following default parameters: -N25 -t15 -r5 -p “4+25*2+4+6,” scaling the output and assuming a mutation rate (μ) of 1.2 × 10^−7^ per site per generation and a generation time of 30 years. Given the uncertainty in generation time estimates across species and the variability reported in the literature for each, we selected a generation time of 30 years as an approximate midpoint of reported values. This choice provides a reasonable and biologically plausible basis for our analyses, as previously tested by Bentley et al. [14]. We conducted an additional PSMC analysis using data exclusively from the 11 macrochromosomes, excluding their identified high-diversity regions (Supplementary Table S9), which may be subject to balancing or diversifying selection, potentially biasing the demographic inferences [112].

Demographic history was further reconstructed using the multiple sequentially Markovian coalescent model (MSMC2; [113]). Input files were prepared with utilities from the MSMC toolkit (https://github.com/stschiff/msmc-tools). Initially, filtered gVCF files, restricted to the 28 autosomes, were processed using the VCFAllSiteParser.py script to create individual masking files. To account for genome regions with reliable read mapping, a mappability mask was generated using SNPable (https://lh3lh3.users.sourceforge.net/snpable.shtml), identifying uniquely mappable loci in the reference genome. The formatted input for MSMC2 was then created using the generate_multihetsep.py script. For robustness, we produced 50 bootstrap replicates with multihetsep_bootstrap.py (parameters: -n 50 -s 20000000 –chunks_per_chromosome 10). MSMC2 analyses were executed using the default time segment pattern (1*2+25*1+1*2+1*3), incorporating both the individual masks and the mappability mask for each chromosome. Effective population size estimates were scaled based on the previously specified per-generation mutation rate and generation time.

### Hotspot gene family annotation

To further refine the annotation of gene families in the identified hotspot regions, we widened the search to include more functional databases available in InterProScan. Using the *D. coriacea* RefSeq annotation, we extracted the amino acid sequence of the longest isoform for each gene using *agat_sp_keep_longest_isoform.pl* and the protein sequence using *agat_sp_extract_sequences.pl* as above and used these protein sequences as input to InterProScan (InterPro v102.0), using the Pfam [114], PRINTS [115], SUPERFAMILY [116], PANTHER [117], Gene3D [118], FunFam [119], and SMART [120] databases. To annotate genes belonging to multicopy gene families, we classified genes as “MHC,” “Immunology-related,” “G-Protein Coupled Receptor” (GPCR), “Olfactory Receptor,” or “Zinc-Finger” following terminology described in Supplementary Table S10. Genes that did not fall into any of these multicopy gene families were classified as “Other.” We then tested the enrichment of these multicopy gene families against all other protein-coding genes annotated via Fisher’s exact test, followed by Benjamini–Hochberg correction of *P* values to account for multiple testing. A full R script is available in the file “enrichment_test.R.”

## Source Code Availability and Requirements

Project name: Sea Turtle Genome Analysis

Project homepage: https://git.imp.fu-berlin.de/begendiv/sea_turtlegenomes

Operating system(s): Unix

Programming languages: R, Python, bash

Other requirements: bioconda, conda-forge, singularity/docker/apptainer

License: MIT

Any restrictions to use by nonacademics: Use in line with MIT License

## Additional Files

**Supplementary Fig. S1**. The *k*-mer–based genome profiling for the sequences of sea turtles. Shown are the distributions of 31-mers in each PacBio HiFi dataset for the sequenced individuals. Overlaid are the genome profile models as calculated by GenomeScope, giving indications of genome size, heterozygosity (ab), nonrepetitive content of the genome (uniq), and estimates of the heterozygous genome coverage (kcov), read error (err), and duplication rate (dup).

**Supplementary Fig. S2**. Genome contiguity statistics of sea turtle genomes. Shown are the contig N50 and N90 values for all available sea turtle genomes. N50 values are shown as circles and N90 values as triangles. Included are the 10 haplotype-separated assemblies from this study, PacBio CLR-based assemblies for *Dermochelys* and *Chelonia*, an ONT-based assembly for *Caretta*, and 1 PacBio HiFi assembly for *Eretmochelys*.

**Supplementary Fig. S3**. Snail plots summary of assembly statistics. The main plot is divided into 1,000 size-ordered bins around the circumference, with each bin representing 0.1% of the total assembly. The distribution of sequence lengths is shown in dark gray, with the plot radius scaled to the longest sequence present in the assembly (shown in red). Orange and pale-orange arcs show the scaffold N50 and N90 sequence lengths, respectively. The pale gray spiral shows the cumulative sequence count on a log-scale, with white scale lines showing successive orders of magnitude. The blue and pale blue area around the outside of the plot shows the distribution of GC, AT, and N percentages in the same bins as the inner plot. A summary of complete, fragmented, duplicated, and missing BUSCO genes found in the assembled genome from the Sauropsida database (odb10) is shown in the top right.

**Supplementary Fig. S4**. Three-dimensional conformational arrangement of the sea turtle genomes as evaluated by Hi-C. The x- and y-axes show the coordinates of the respective genome, and each detected contact in the genome is colored with increasing intensity from white to red. The red diagonal shows the self-interactions of each position with itself and close vicinity.

**Supplementary Fig. S5**. Gene completeness of sea turtle genomes. Shown are the percentages of detected single-copy orthologs calculated via BUSCO using the Sauropsida database. Scores are calculated based on detected mappings of ortholog sequences using Metaeuk (above) and Miniprot (below). Note the Miniprot mode of BUSCO also gives estimates of BUSCO genes potentially containing early STOP codons.

**Supplementary Fig. S6**. Gene completeness of sea turtle protein-coding annotations. Shown are the percentages of Sauropsida Hierarchical Orthology Groups (above) and single-copy orthologs (below) as calculated by OMArk and BUSCO, respectively, from the annotated protein sequences in each sea turtle genome.

**Supplementary Fig. S7**. Genome-wide gene-synteny plots for sea turtles. Each line represents a best-reciprocal-hit protein match between annotated genes in each consecutive genome. Lines are colored based on colocalization across all 7 genomes determined by the Fisher exact test. Chromosomes are ordered based on synteny to the *Dermochelys coriacea* genome.

**Supplementary Fig. S8**. Genome-wide gene-synteny plots across testudines. Each line represents a best-reciprocal-hit protein match between annotated genes in each consecutive genome. Lines are colored based on colocalization across all 11 genomes determined by the Fisher exact test. Chromosomes are ordered based on the assigned chromosome name in GenBank.

**Supplementary Fig. S9**. Variation of genome-wide heterozygosity (He) across the 28 chromosomes for the 7 sea turtle species, depicting He of nonoverlapping 50-kb windows with RoH segments highlighted in gray. Chromosomes are represented with equal widths; therefore, RoH sizes are not proportional among them, as depicted in the figure.

**Supplementary Fig. S10**. Pairwise genetic distances between different sea turtle species along the 28 chromosomes. The first species listed in each comparison refers to the genome considered the reference in the Cactus alignment. Genetic distance was calculated as the ratio of interspecific single variants per 30-kb window. Red dots represent windows with genetic distance greater than 2× the average. Chromosomes are shown with equal widths.

**Supplementary Fig. S11**. Pairwise genetic distances between different sea turtle species along the chromosomes 13, 14, and 24. The first species listed in each comparison refers to the genome considered as the reference in the Cactus alignment. Genetic distance was calculated as the ratio of interspecific single variants per 10-kb window, with normalization applied to highlight chromosomal hotspots rather than overall species divergence.

**Supplementary Fig. S12**. Ribbon plot showing the locations of syntenic genes between the previous available genome from *Caretta caretta* (top) and the 2 haplotype assemblies presented in this study.

**Supplementary Fig. S13**. Ribbon plot showing the locations of syntenic genes between all assemblies presented in this study.

**Supplementary Fig. S14**. Ancient demographic history for the 5 sea turtle genomes assembled in this study reconstructed with the pairwise sequential Markovian coalescent (PSMC). This analysis focused exclusively on the 11 macrochromosomes, excluding regions of high genetic diversity (Supplementary Table S9). Dashed lines indicate the middle points of the Last Interglacial (Eemian Period, 130,000 to 115,000 years ago) and mid-Pleistocene transition (1.2–0.5 million years ago). Bootstrap replicates (10 for each lineage) are plotted in lighter lines. Inferred fluctuations in effective population size (Ne) were rescaled assuming 3a 0-year generation time and 1.2 × 10^−8^ per generation mutation rate.

**Supplementary Fig. S15**. Ancient demographic history for the 7 sea turtle reference genomes inferred using MSMC2. Shades indicate the Last Interglacial (Eemian Period, 0.13–0.115 million years ago) and mid-Pleistocene transition (1.2–0.5 million years ago). Bootstrap replicates (50 for each species) are plotted in lighter lines. Both axes were constrained for clear visualization. Inferred fluctuations in effective population size (Ne) were rescaled assuming a 30-year generation time and 1.2 × 10^−8^ per generation mutation rate.

**Supplementary Table S1**. PacBio HiFi and OmniC Hi-C sequencing coverage after adapter and quality trimming.

**Supplementary Table S2**. Assembly metrics for all the available sea turtle assemblies.

**Supplementary Table S3**. Statistics from protein-coding annotations.

**Supplementary Table S4**. Divergence time estimates for all nodes of the phylogenetic tree shown in Figure 3.

**Supplementary Table S5**. Heterozygosity, inbreeding level (FROH) and gene density per chromosome, for the 7 sea turtle reference genomes. These data are provided in a separate file in Excel format.

**Supplementary Table S6**. Enrichment *P* values for multicopy gene families in hotspots of genetic diversity

**Supplementary Table S7**. Benjamini–Hochberg corrected *P* values for multicopy gene families in hotspots of genetic diversity.

**Supplementary Table S8**. Weights used to combine transcriptome evidences into a single gene-model set.

**Supplementary Table S9**. Chromosomes and regions included in the PSMC analysis for each sea turtle species. These regions excluded the identified high-diversity regions that may be subject to balancing or diversifying selection, which can bias the demographic inferences.

**Supplementary Table S10**. Database and terminology used for classifying genes based on their functional annotation.

giaf105_giaf105_Supplemental_Files

giaf105_Authors_Response_To_Reviewer_Comments_Original_Submission

giaf105_Authors_Response_To_Reviewer_Comments_Revision_1

giaf105_GIGA-D-25-00103_Original_Submission

giaf105_GIGA-D-25-00103_Revision_1

giaf105_GIGA-D-25-00103_Revision_2

giaf105_Reviewer_1_Report_Original_Submission Laura Caquelin -- 4/9/2025

giaf105_Reviewer_2_Report_Original_Submission Brendan Reid -- 4/10/2025

giaf105_Reviewer_3_Report_Original_Submission Xiaoli Liu -- 4/21/2025

giaf105_Reviewer_3_Report_Revision_1 Xiaoli Liu -- 4/21/2025

## Abbreviations

BUSCO: Benchmarking Universal Single Copy Orthologs; CLR: continuous long reads; ENA: European Nucleotide Archive; FROH: fraction of genome in runs of homozygosity; GPCR: G-protein coupled receptor; gVCF: genomic variant call format; HiFi: Hi-fidelity circular consensus sequence; HPD: highest posterior density; IUCN: International Union for Conservation of Nature; kb: kilobase; kya: thousand years ago; Mb: megabase; MHC: major histocompatibility complex; MSMC: multiple sequentially Markovian coalescent; mya: million years ago; NCBI: National Center for Biotechnology Information; N_e_: effective population size; NuMT: nuclear mitochondrial DNA; ONT: Oxford Nanopore Technologies; OR: olfactory receptor; PacBio: Pacific Biosciences; PSMC: pairwise sequentially Markovian coalescent; QV: quality value; RMU: regional management unit; ROH: run of homozygosity; SMRT: sequencing molecule, real-time; SNP: single-nucleotide polymorphism.

## Declarations

*N. depressus* blood was collected under permit (TFA 2019-0174-2), obtained animal ethics committee approval (2019-12B), and was shipped under CITES permit (AU94). *L. kempii* blood and tissue samples were collected under USFWS permit ES69328D. *E. imbricata* blood was collected under USFWS permit TE-72088A-3. *C. caretta* and *L. olivacea* blood was collected under USFWS permit TE86356B-2 (to Sea World), and *C. caretta* embryo tissue samples were collected under Florida Fish and Wildlife Conservation Commission Marine Turtle Permit 073 (FWC-MTP-073).

## Competing Interests

The authors declare no competing interests.

## Data Availability

Sequencing data, genome assemblies, and annotations are available via the European Nucleotide Archive (ENA) or National Center for Biotechnology Information (NCBI) under the following umbrella BioProjects: *Caretta caretta* PRJNA1212178, *Eretmochelys imbricata* PRJNA1212183, *Lepidochelys kempii* PRJNA1212180, *Lepidochelys olivacea* PRJNA1212179, and *Natator depressus* PRJNA1212185. Workflows used to generate genome assemblies are published on WorkflowHub under the following collection: [121–127] and including the BioNano scaffolding workflow from the Vertebrate Genomes Project [128]. To perform read mapping and SNP calling as well as calculating runs of homozygosity, we used the jATG pipeline available on GitHub: [104]. Other data further supporting this work are openly available in the *GigaScience* repository, GigaDB [129]. Data supporting *Caretta caretta* are available at [130], data supporting *Eretmochelys imbricata* are available at [131], data supporting *Lepidochelys kempii* are available at [132], data supporting *Lepidochelys olivacea* are available at [133], and data supporting *Natator depressus* are available at [134].
