# Supplementary material for: Haplotype-resolved reference genomes of the sea turtle clade unveil ultra-syntenic genomes with hotspots of divergence
Source: Gigascience. 2025 Sep 18;14:giaf105. doi: 10.1093/gigascience/giaf105 (PMC12448945; doi:10.1093/gigascience/giaf105)
Supplement: giaf105_giaf105_Supplemental_Files [file giaf105_giaf105_supplemental_files.zip › Turtle_SuppMat - Revision 1.pdf]

Supplementary Material

Haplotype-resolved reference genomes of the sea turtle clade unveil ultra-syntenic genomes with hotspots of divergence

Supplementary Figures

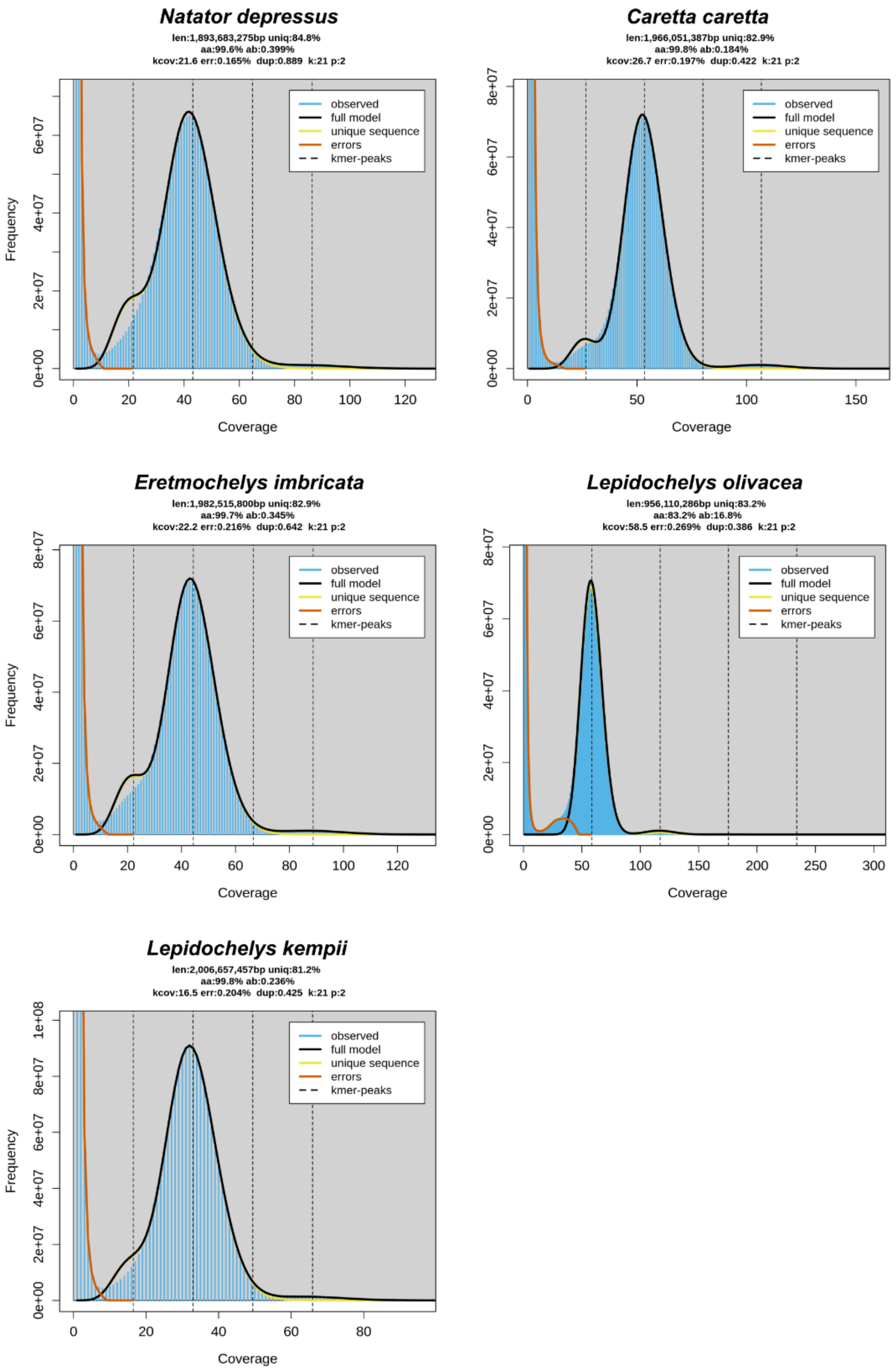

Natator depressus
len:1,893,683,275bp uniq:84.8%
aa:99.6% ab:0.399%
kcov:21.6 err:0.165% dup:0.889 k:21 p:2
Caretta caretta
len:1,966,051,387bp uniq:82.9%
aa:99.8% ab:0.184%
kcov:26.7 err:0.197% dup:0.422 k:21 p:2
Eretmochelys imbricata
len:1,982,515,800bp uniq:82.9%
aa:99.7% ab:0.345%
kcov:22.2 err:0.216% dup:0.642 k:21 p:2
Lepidochelys olivacea
len:956,110,286bp uniq:83.2%
aa:83.2% ab:16.8%
kcov:58.5 err:0.269% dup:0.386 k:21 p:2
Lepidochelys kempii
len:2,006,657,457bp uniq:81.2%
aa:99.8% ab:0.236%
kcov:16.5 err:0.204% dup:0.425 k:21 p:2
observed
full model
unique sequence
errors
kmer-peaks
Frequency
Coverage

Sup. Fig. 1 K-mer based genome profiling for the sequences sea-turtles. Shown are the distributions of 31-mers in each PacBio HiFi dataset for the sequenced individuals. Overlaid are the genome profile models as calculated by GenomeScope, giving indications of genome size, heterozygosity (ab), non-repetitive content of the genome (uniq) as well as estimates of the heterozygous genome coverage (kcov), read error (err) and duplication rate (dup)

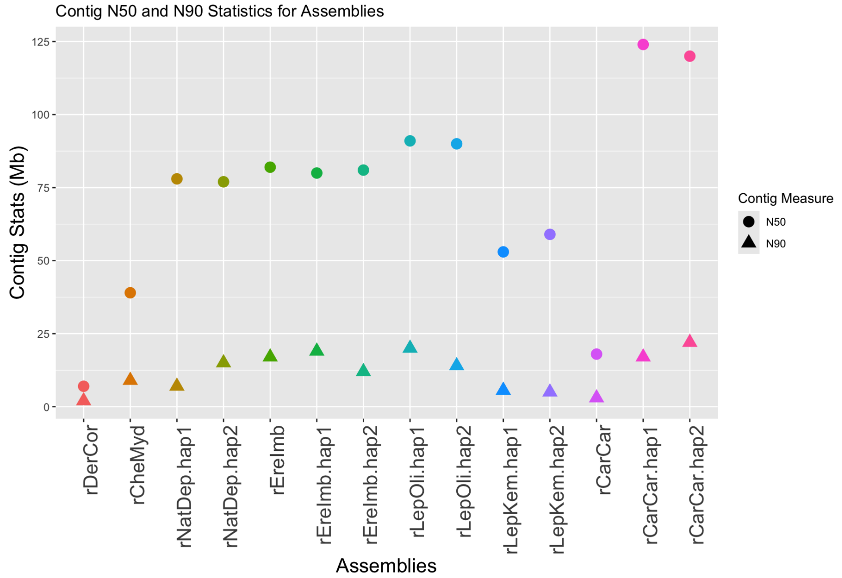

Sup. Fig. 2 Genome contiguity statistics of sea turtle genomes. Shown are the Contig N50 and N90 values for all available sea-turtle genomes. N50 values are shown as circles and N90 values as triangles. Included are the 10 haplotype-separated assemblies from this study, PacBio CLR-based assemblies for *Dermochelys* and *Chelonia*, an ONT-based assembly for *Caretta* and one PacBio HiFi assembly for *Eretmochelys*.

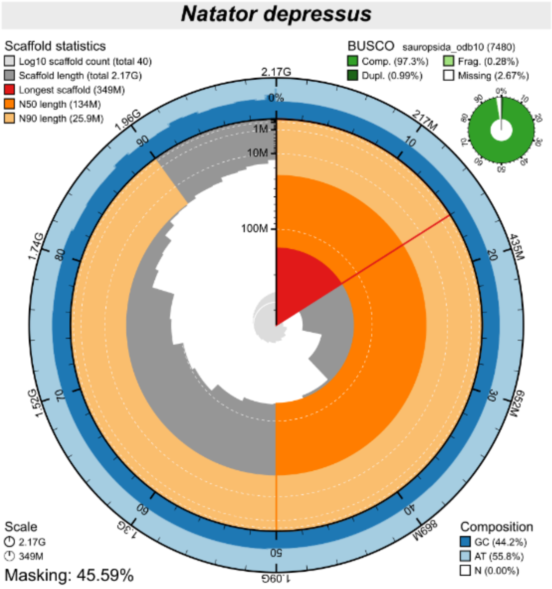
Natator depressus
Scaffold statistics
Log10 scaffold count (total 40)
Scaffold length (total 2.17G)
Longest scaffold (349M)
N50 length (134M)
N90 length (25.9M)
BUSCO sauropsida_odb10 (7480)
Comp. (97.3%)
Frag. (0.28%)
Dupl. (0.99%)
Missing (2.67%)
Scale
2.17G
349M
Composition
GC (44.2%)
AT (55.8%)
N (0.00%)
Masking: 45.59%

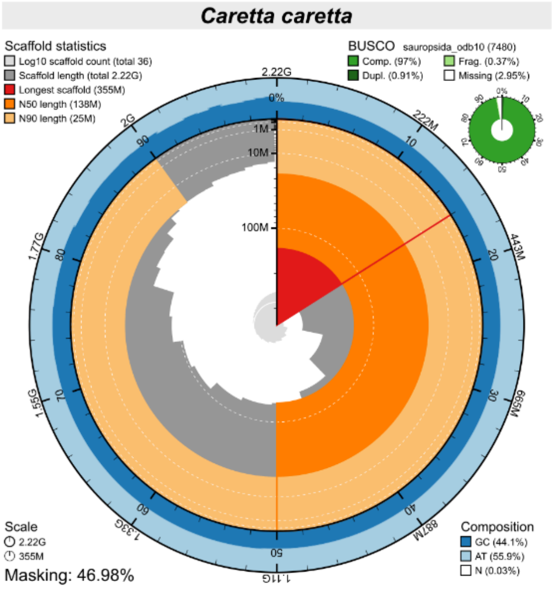
Caretta caretta
Scaffold statistics
Log10 scaffold count (total 36)
Scaffold length (total 2.22G)
Longest scaffold (355M)
N50 length (138M)
N90 length (25M)
BUSCO sauropsida_odb10 (7480)
Comp. (97%)
Frag. (0.37%)
Dupl. (0.91%)
Missing (2.95%)
Scale
2.22G
355M
Composition
GC (44.1%)
AT (55.9%)
N (0.03%)
Masking: 46.98%

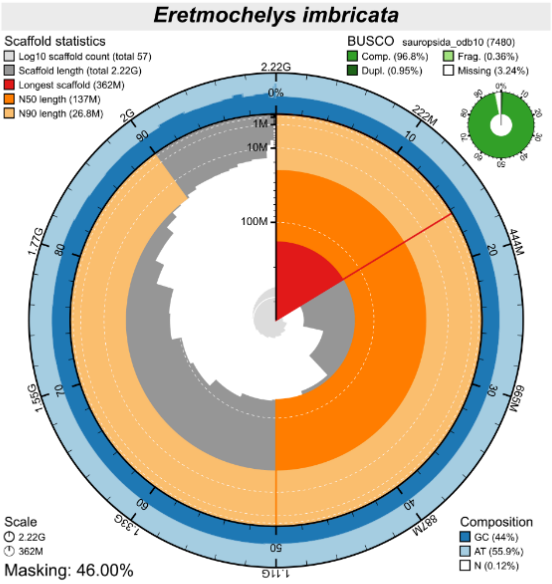
Eretmochelys imbricata
Scaffold statistics
Log10 scaffold count (total 57)
Scaffold length (total 2.22G)
Longest scaffold (362M)
N50 length (137M)
N90 length (26.8M)
BUSCO sauropsida_odb10 (7480)
Comp. (96.8%)
Frag. (0.36%)
Dupl. (0.95%)
Missing (3.24%)
Scale
2.22G
362M
Composition
GC (44%)
AT (55.9%)
N (0.12%)
Masking: 46.00%

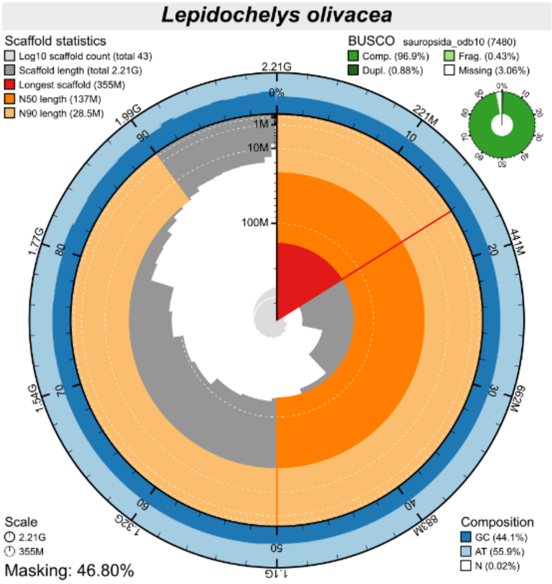
Lepidochelys olivacea
Scaffold statistics
Log10 scaffold count (total 43)
Scaffold length (total 2.21G)
Longest scaffold (355M)
N50 length (137M)
N90 length (28.5M)
BUSCO sauropsida_odb10 (7480)
Comp. (96.9%)
Frag. (0.43%)
Dupl. (0.88%)
Missing (3.06%)
Scale
2.21G
355M
Composition
GC (44.1%)
AT (55.9%)
N (0.02%)
Masking: 46.80%

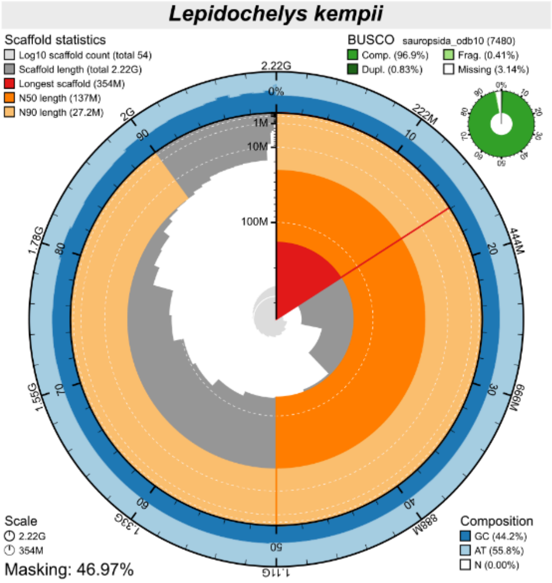
Lepidochelys kempii
Scaffold statistics
Log10 scaffold count (total 54)
Scaffold length (total 2.22G)
Longest scaffold (354M)
N50 length (137M)
N90 length (27.2M)
BUSCO sauropsida_odb10 (7480)
Comp. (96.9%)
Frag. (0.41%)
Dupl. (0.83%)
Missing (3.14%)
Scale
2.22G
354M
Composition
GC (44.2%)
AT (55.8%)
N (0.00%)
Masking: 46.97%

Sup. Fig. 3 Snail plots summary of assembly statistics. The main plot is divided into 1,000 size-ordered bins around the circumference, with each bin representing 0.1% of the total assembly. The distribution of sequence lengths is shown in dark grey, with the plot radius scaled to the longest sequence present in the assembly (shown in red). Orange and pale-orange arcs show the scaffold N50 and N90 sequence lengths, respectively. The pale grey spiral shows the cumulative sequence count on a log-scale, with white scale lines showing successive orders of magnitude. The blue and pale-blue area around the outside of the plot shows the distribution of GC, AT, and N percentages in the same bins as the inner plot. A summary of complete, fragmented, duplicated, and missing BUSCO genes found in the assembled genome from the Sauropsida database (odb10) is shown in the top right.

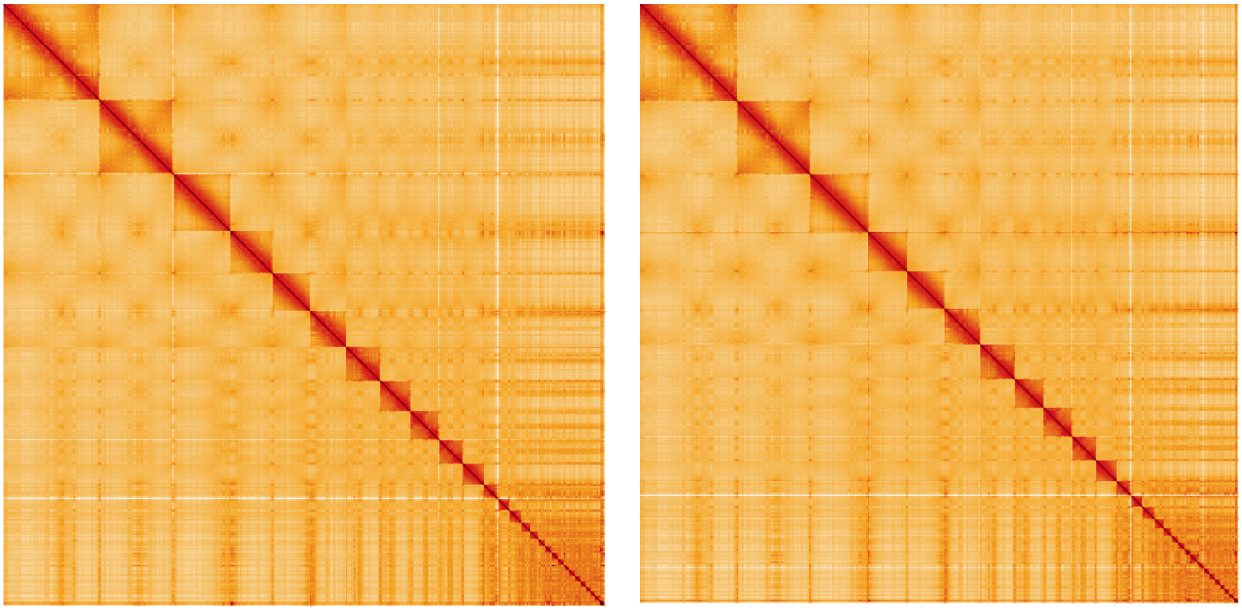

Caretta caretta Hi-C interactions

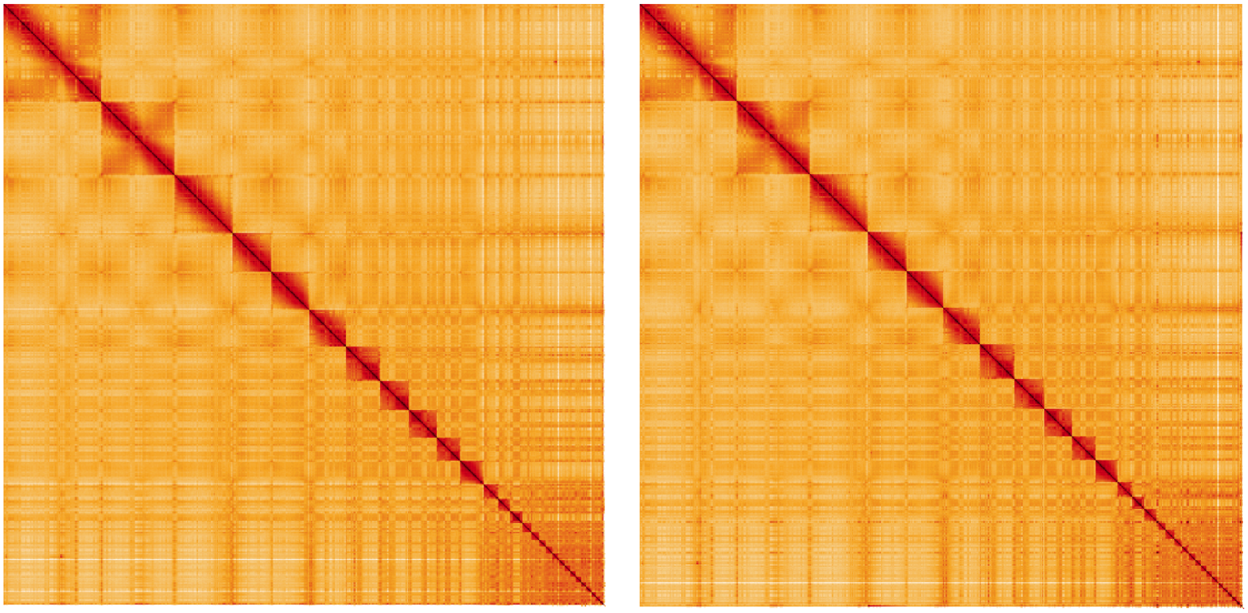

Eretmochelys imbricata Hi-C interactions

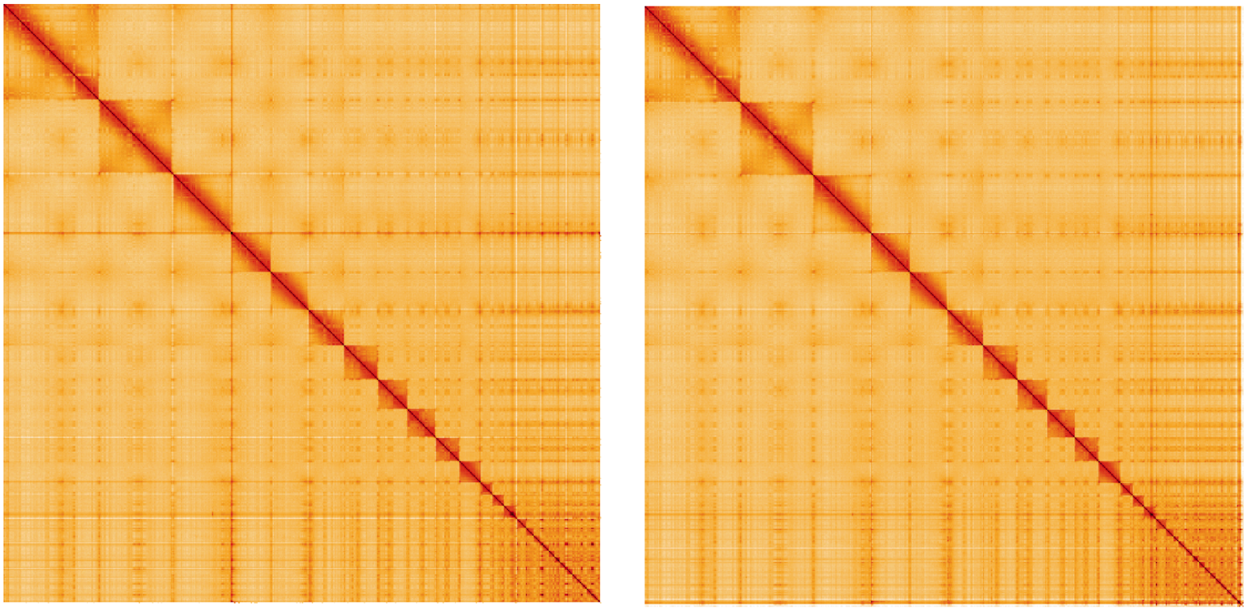

Lepidochelys olivacea Hi-C interactions

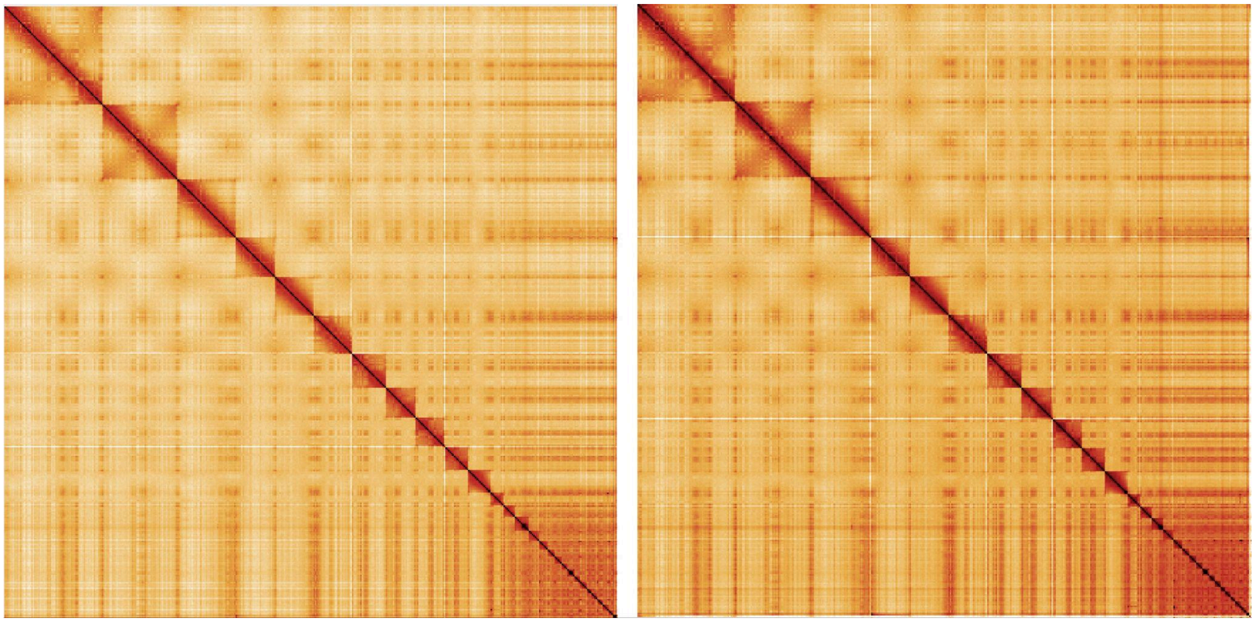

Lepidochelys kempii Hi-C interactions

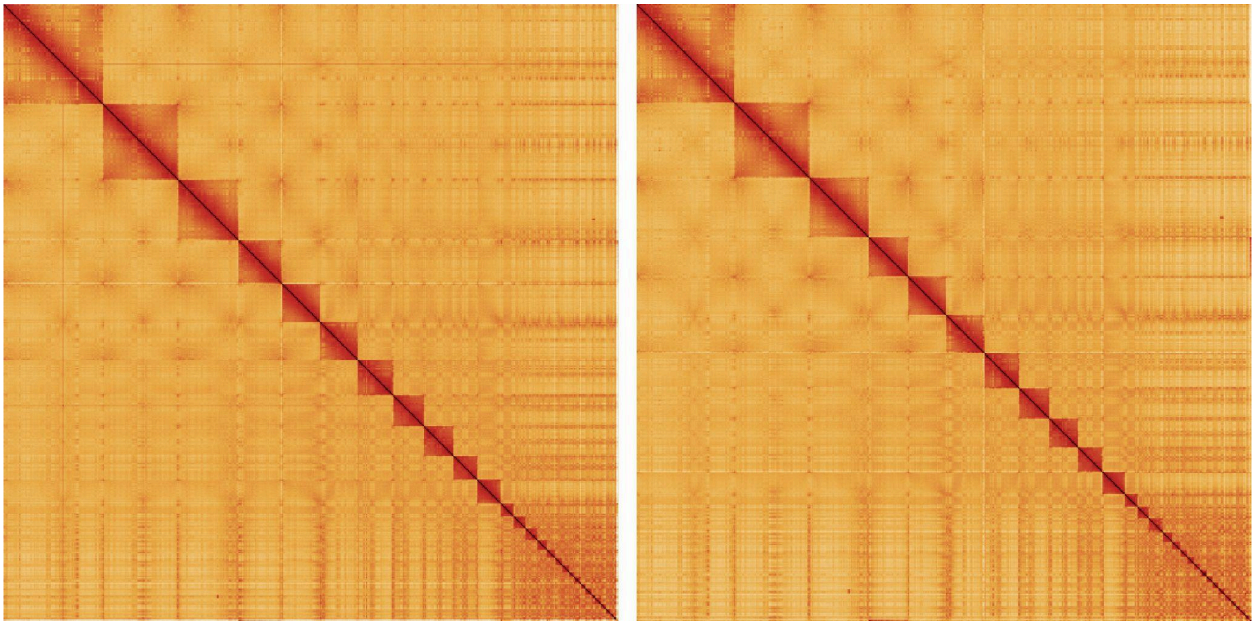

Natator depressus Hi-C interactions

Sup. Fig. 4 3-dimensional conformational arrangement of the sea-turtle genomes as evaluated by Hi-C. The x- and y-axes show the coordinates of the respective genome and each detected contact in the genome is coloured with increasing intensity from white to red. The red diagonal shows the self-interactions of each position with itself and close vicinity.

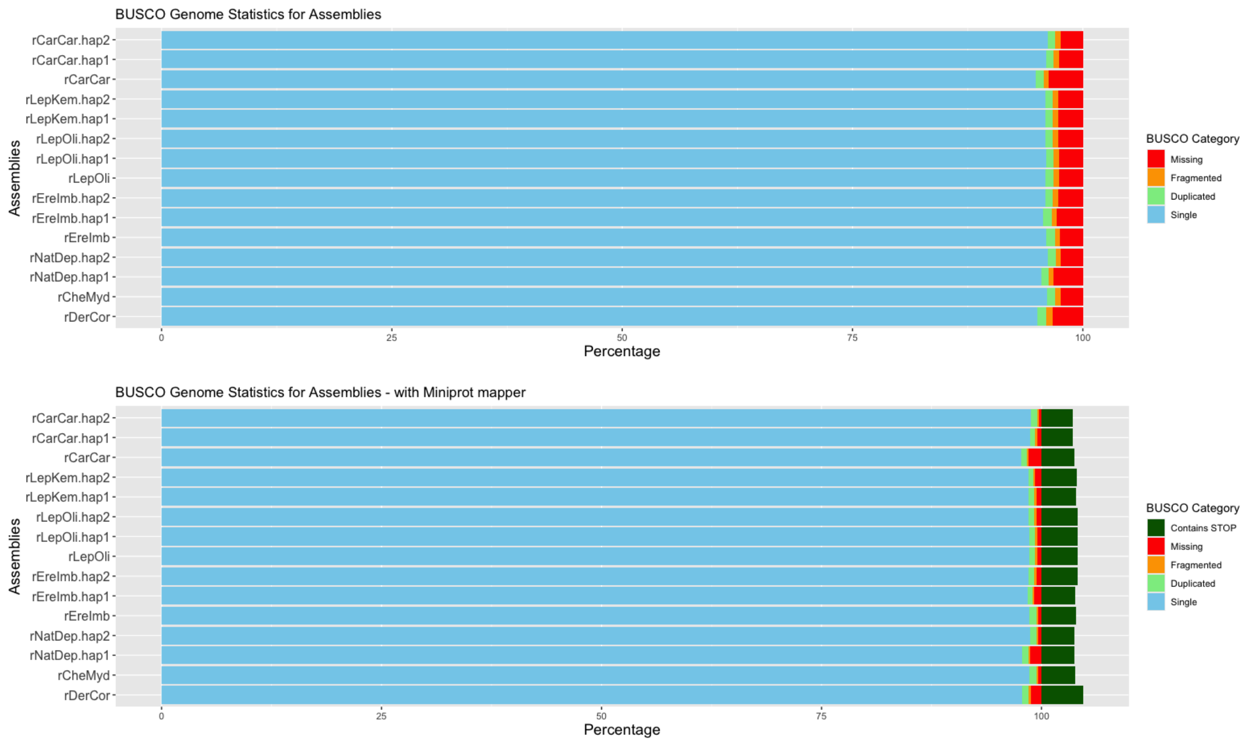

Sup. Fig. 5 Gene completeness of sea-turtle genomes. Shown are the percentage detected single copy orthologs calculated via BUSCO using the Sauropsida database. Scores are calculated based on detected mappings of ortholog sequences using Metaeuk (above) and Miniprot (below). Note the Miniprot mode of BUSCO also gives estimates of BUSCO genes potentially containing early STOP codons.

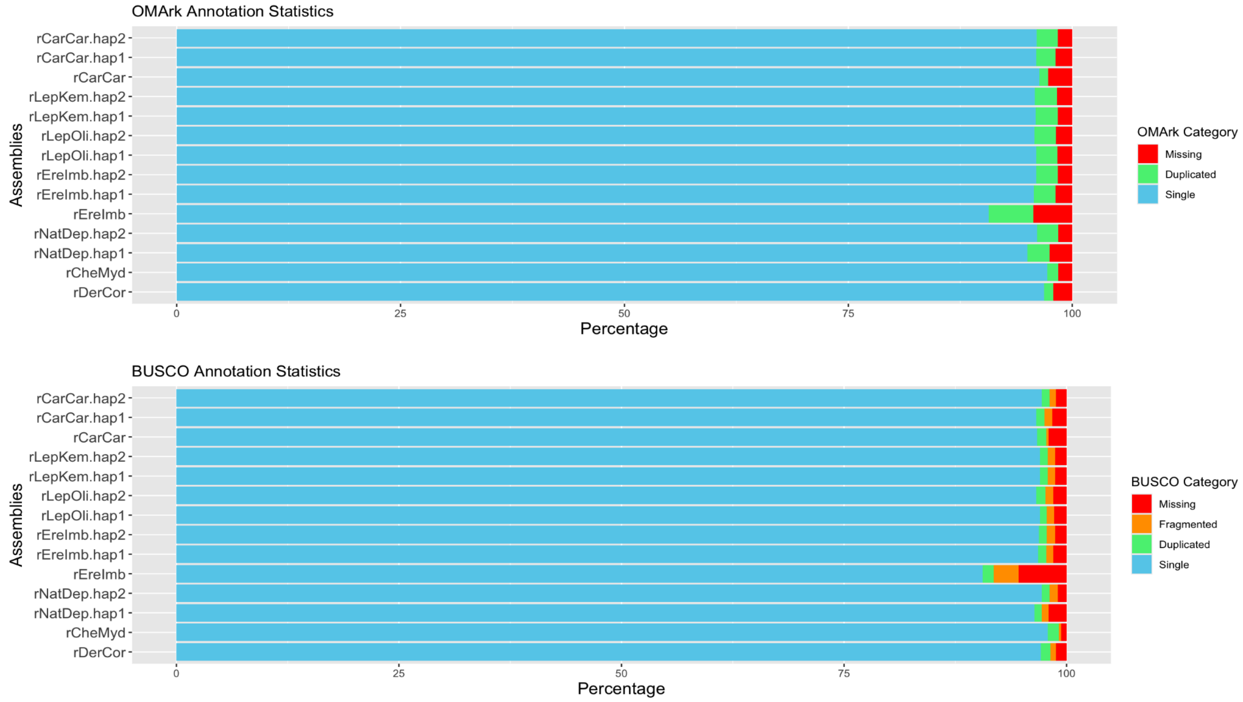

Sup. Fig. 6 Gene completeness of sea-turtle protein-coding annotations. Shown are the

percentage Sauropsida Hierarchical Orthology Groups (above) and single copy orthologs (below) as calculated by OMArk and BUSCO, respectively, from the annotated protein sequences in each sea-turtle genome.

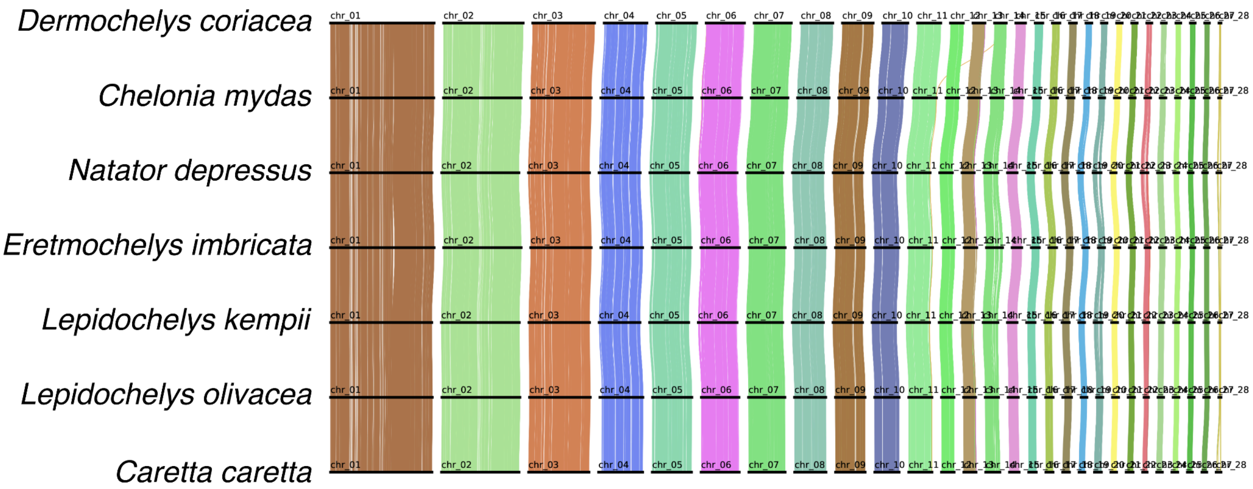

Sup. Fig. 7 Genome-wide gene-synteny plots for sea turtles. Each line represents a best-reciprocal-hit protein match between annotated genes in each consecutive genome. Lines are coloured based on co-localisation across all 7 genomes determined by Fisher's Exact Test. Chromosomes are ordered based on synteny to *Dermochelys coriacea* genome.

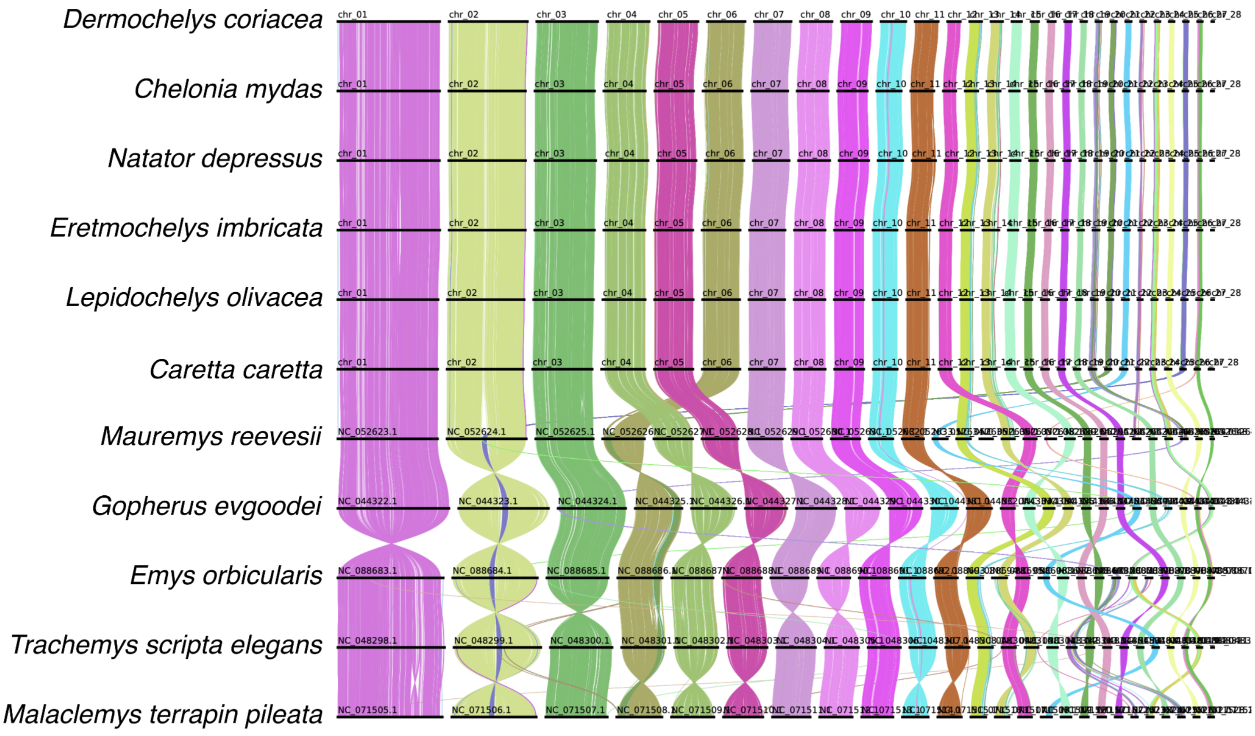

Sup. Fig. 8 Genome-wide gene-synteny plots across testudines. Each line represents a best-reciprocal-hit protein match between annotated genes in each consecutive genome. Lines are coloured based on co-localisation across all 11 genomes determined by Fisher's Exact Test. Chromosomes are ordered based on the assigned chromosome name in GenBank.

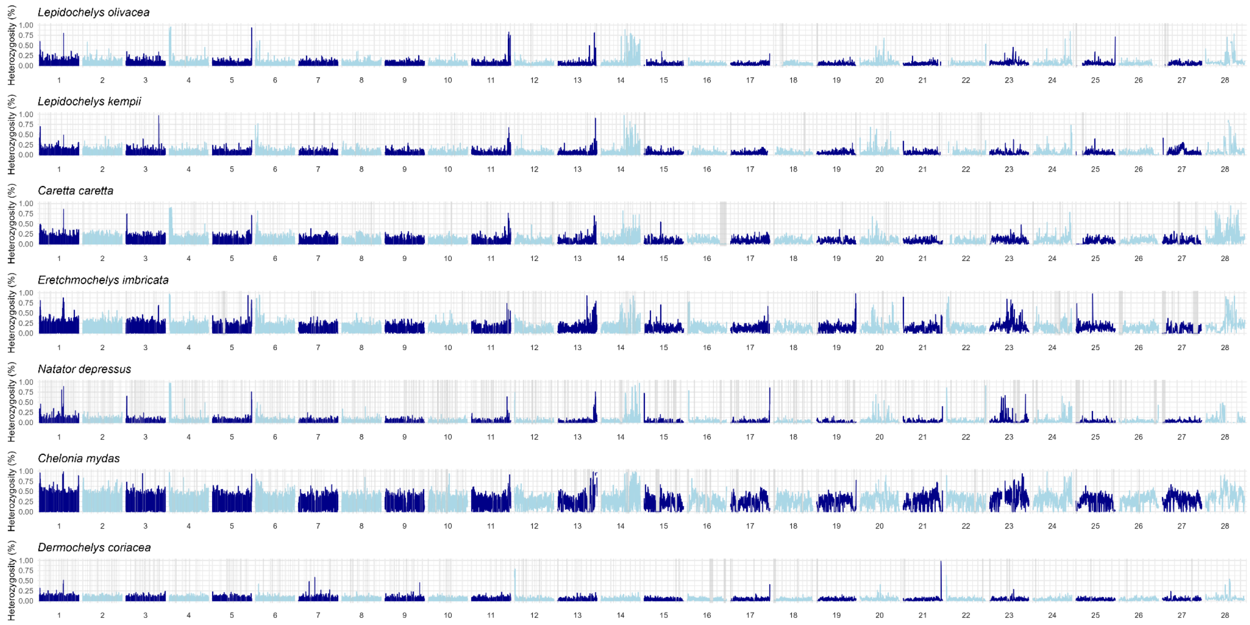

Sup. Fig. 9 Variation of genome-wide heterozygosity (He) across the 28 chromosomes for the seven sea turtle species, depicting He of non-overlapping 50 kb windows with RoH segments highlighted in gray. Chromosomes are represented with equal widths, therefore RoHs sizes are not proportional among them as depicted in the figure.

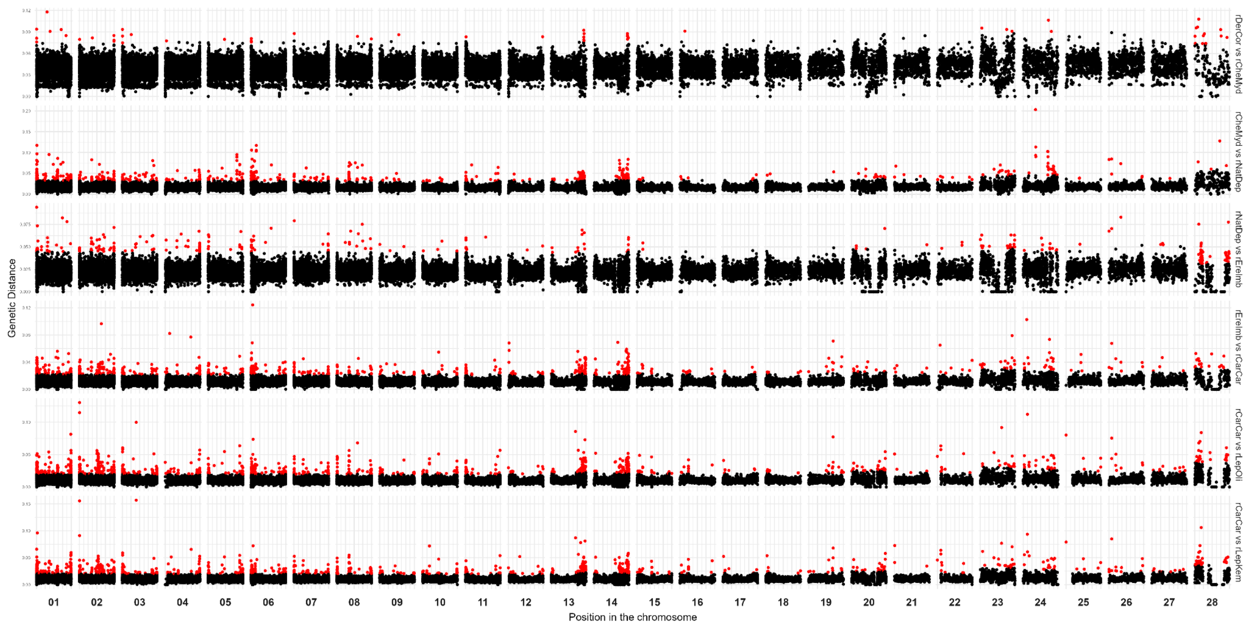

Sup. Fig. 10 Pairwise genetic distances between different sea turtle species along the 28 chromosomes. The first species listed in each comparison refers to the genome considered as the reference in the Cactus alignment. Genetic distance was calculated as the ratio of interspecific single variants per 30 kb window. Red dots represent windows with genetic distance greater than 2x the average. Chromosomes are shown with equal widths.

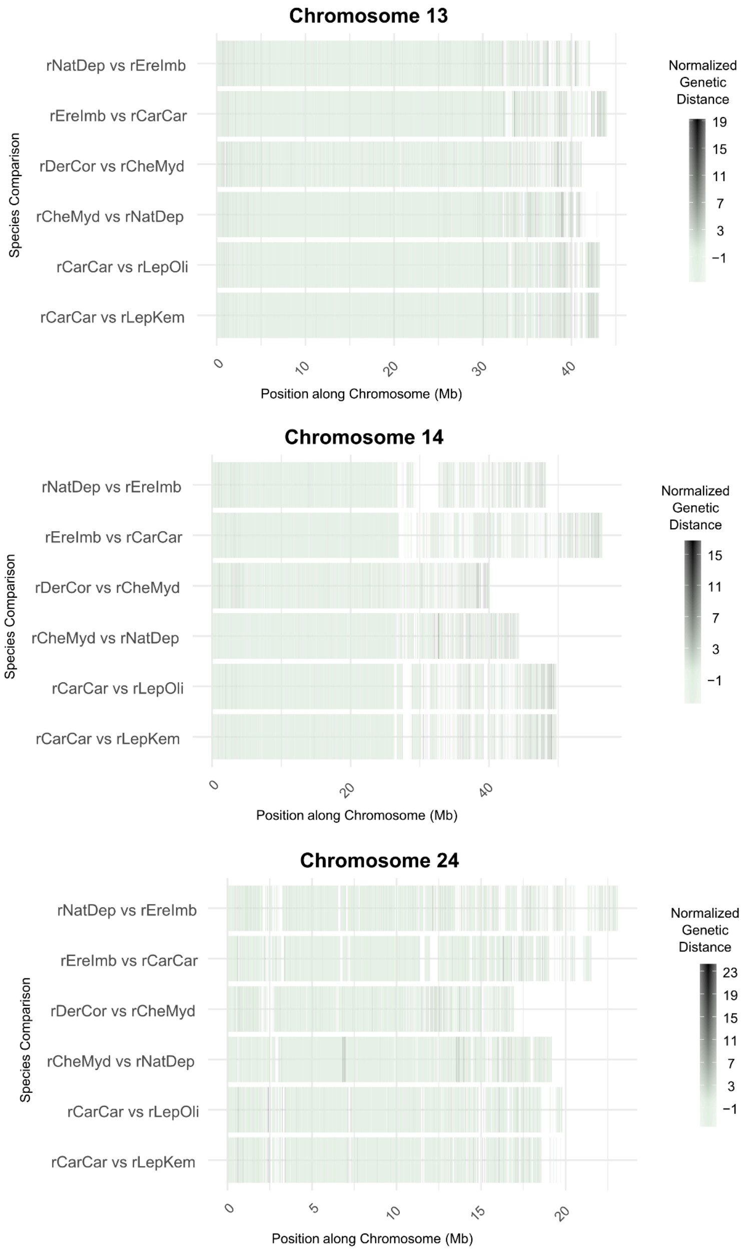

Sup. Fig. 11 Pairwise genetic distances between different sea turtle species along the chromosomes 13, 14 and 24. The first species listed in each comparison refers to the genome considered as the reference in the Cactus alignment. Genetic distance was calculated as the ratio of interspecific single variants per 10 kb window, with normalization applied to highlight chromosomal hotspots rather than overall species divergence.

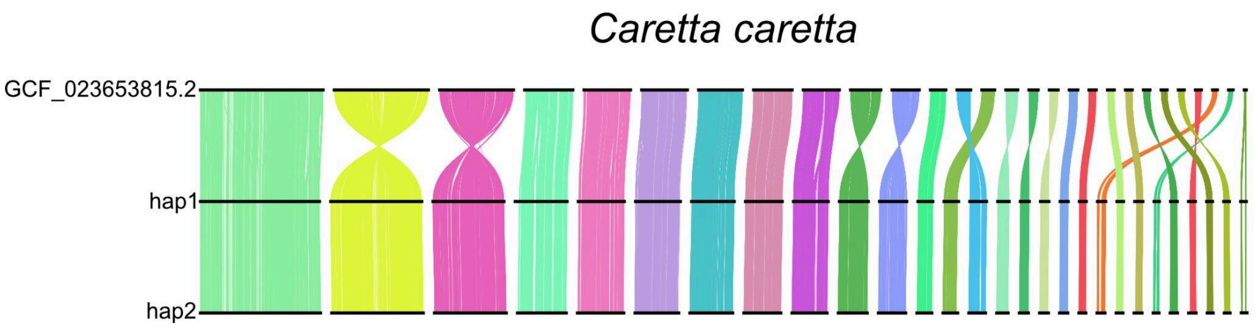

Sup. Fig. 12 Ribbon plot showing the locations of syntenic genes between the previous available genome from *Caretta caretta* (top) and the two haplotype assemblies presented in this study

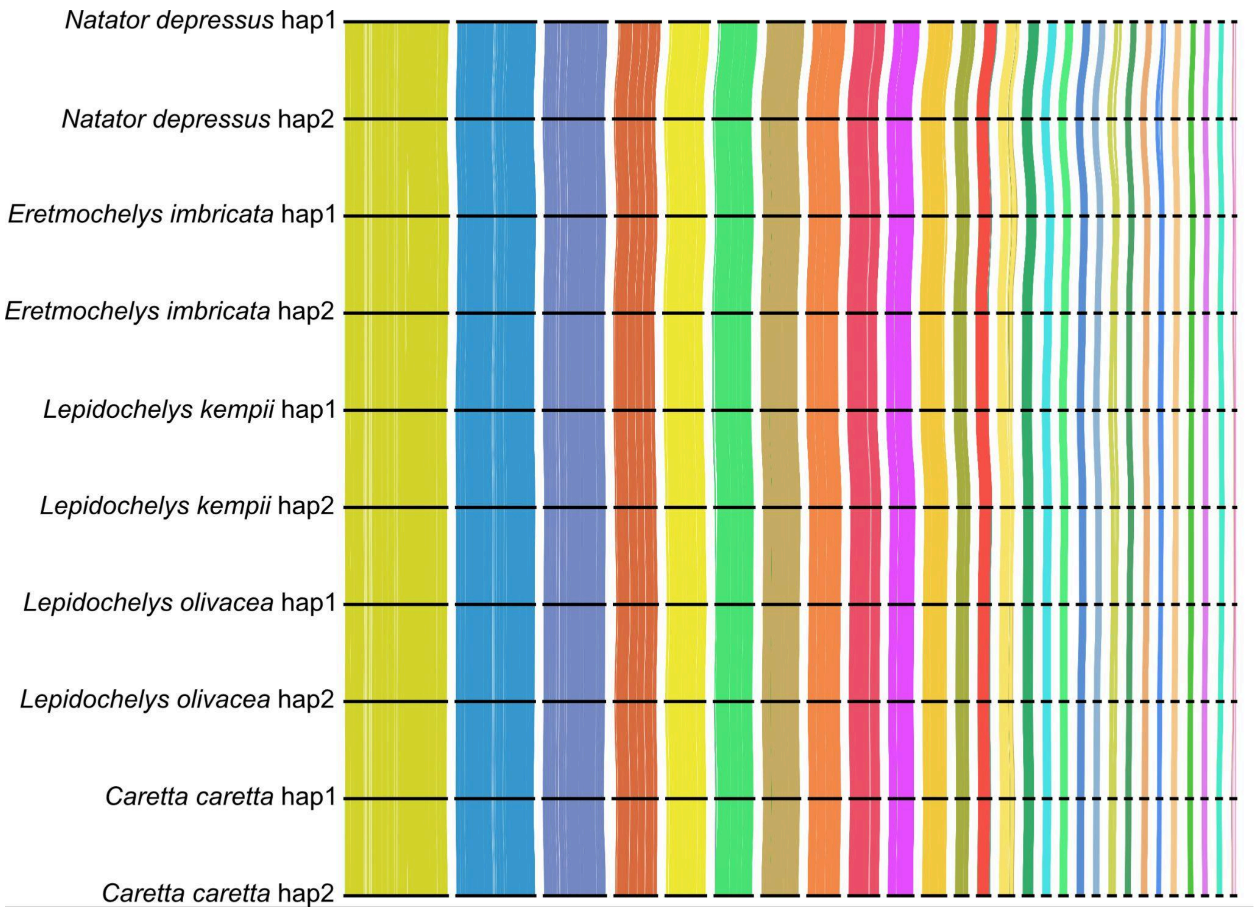

Sup. Fig. 13 Ribbon plot showing the locations of syntenic genes between all as assemblies presented in this study.

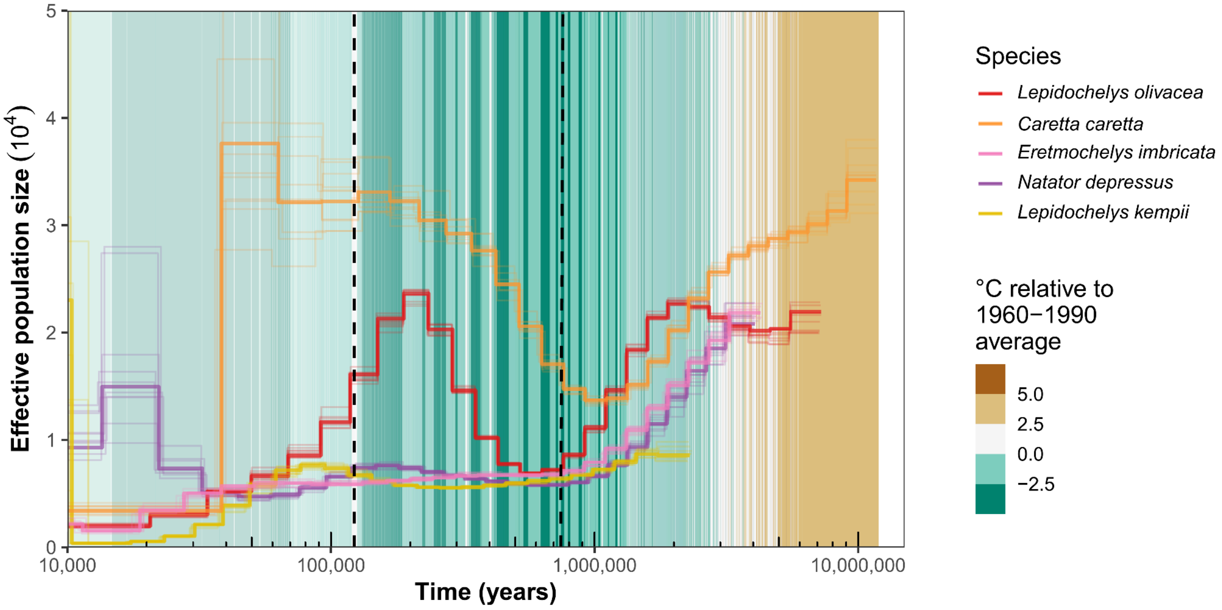

Sup. Fig. 14 Ancient demographic history for the five sea turtle genomes assembled in this study reconstructed with Pairwise Sequential Markovian Coalescent (PSMC). This analysis focused exclusively on the 11 macrochromosomes, excluding regions of high genetic diversity (Table S9). Dashed lines indicate the middle points of the Last Interglacial (Eemian Period, 130,000 to 115,000 years ago) and mid-Pleistocene transition (1.2-0.5 million years ago). Bootstrap replicates (10 for each lineage) are plotted in lighter lines. Inferred fluctuations in effective population size (Ne) were rescaled assuming 30-year generation time and 1.2×10^{-8} per generation mutation rate.

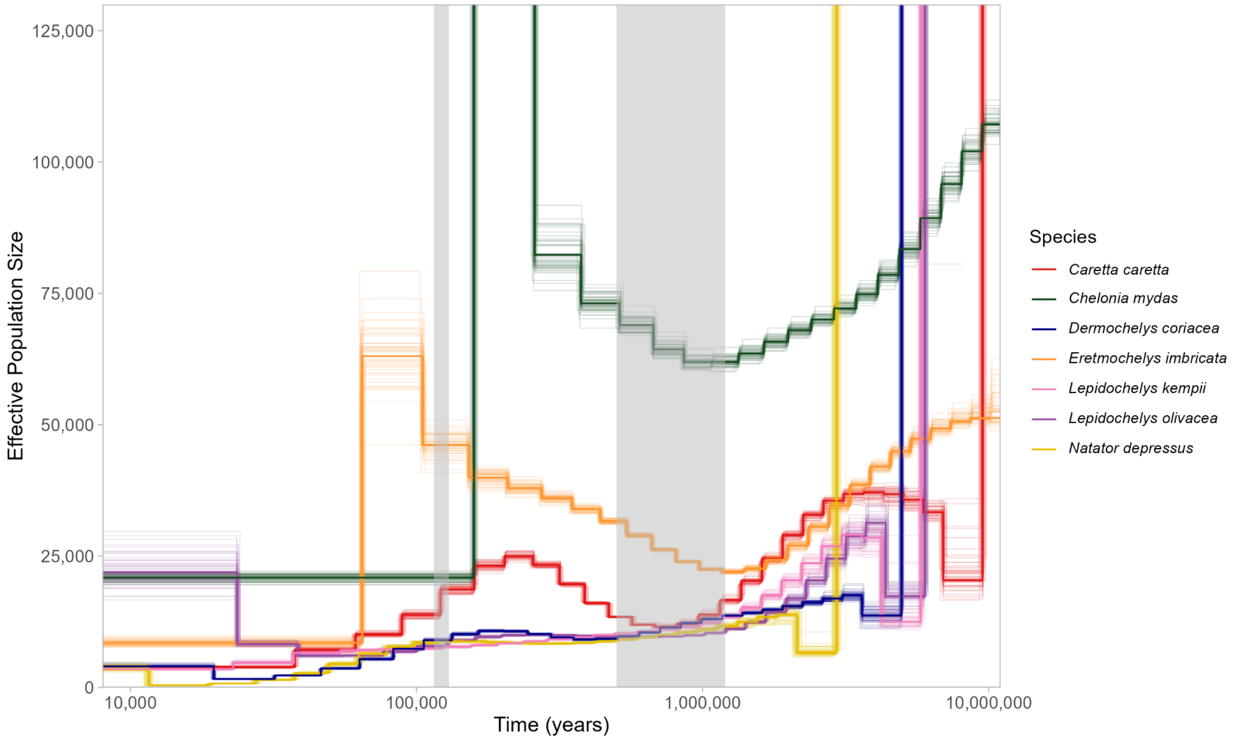

Sup. Fig. 15 Ancient demographic history for the seven sea turtle reference genomes inferred using MSMC2. Shades indicate the Last Interglacial (Eemian Period, 0.13-0.115 million years ago) and mid-Pleistocene transition (1.2-0.5 million years ago). Bootstrap replicates (50 for each species) are plotted in lighter lines. Both axes were constrained for clear visualization. Inferred fluctuations in effective population size (Ne) were rescaled assuming 30-year generation time and 1.2 × 10−8 per generation mutation rate.

Supplementary Tables

Table S1 PacBio HiFi and OmniC Hi-C sequencing coverage after adapter and quality trimming.

	HiFi		Hi-C		Bionano	
Asm ID	bp	x	bp	x	Gbp (≥150kbp)	x
rCarCar	121,592,450,120	55	264,606,173,591	120	507.12	231
rEreImb	101,064,681,912	46	283,870,332,269	129	109.12	50
rLepKem	76,007,057,258	35	102,633,790,121	47	-	-
rLepOli	132,137,130,189	60	188,733,679,226	86	521.74	237
rNatDep	91,008,047,949	41	166,770,516,289	76	-	-

Table S2 Assembly metrics for all the available sea turtle assemblies.
Excel format.

Table S3 Statistics from protein-coding annotations.

Genome	No. genes	Mean gene length (bp)	Mean transcript length (bp)	Mean no. exons	No. single-exon genes
**rCarCar.hap1**	24,378	40,283	1,663	8.8	4,669
**rCarCar.hap2**	26,029	37,848	1,683	8.3	5,985
**rEreImb.hap1**	25,692	38,220	1,585	8.3	5,682
**rEreImb.hap2**	25,792	38,033	1,589	8.4	5,485
**rLepKem.hap1**	26,436	37,158	1,620	8.2	6,372
**rLepKem.hap2**	25,992	37,419	1,622	8.2	6,360
**rLepOli.hap1**	21,475	44,628	1,715	9.7	2,432
**rLepOli.hap2**	24,071	40,107	1,671	8.9	4,446
**rNatDep.hap1**	25,069	37,948	1,618	8.2	5,969
**rNatDep.hap2**	26,687	36,581	1,626	8.2	6,158

Table S4 Divergence time estimates for all nodes of the phylogenetic tree shown in figure 3.

Node	Mean divergence time (MYA)	95% highest posterior density (HPD) interval (MYA)
1. Cryptodira	164	147, 182
2. Durocryptodira	104	81.9, 122

3. (Geoemydidae + Testudinidae) / (Platysternidae + Emydidae)	79.6	65.4, 94.5
4. Geoemydidae / Testudinidae	57.1	52.2, 61.3
5. Platysternidae / Emydidae	55	29.5, 83.6
6. *Chelonoidis abingdonii* / *Gopherus* sp.	48.4	39.0, 58.1
7. Emydidae	39.7	23.4, 56.8
8. *Gopherus flavomarginatus* / *Gopherus evgoodei*	24.1	0.00087, 46.9
9. *Chrysemys picta* / (*Malaclemys terrapin* + *Trachemys scripta*)	23.7	12.6, 34.4
10. *Terrapene triunguis* / *Emys orbicularis*	23.2	11.8, 36.1
11. *Mauremys reevesii* / *Mauremys mutica*	21.7	15.6, 27.9
12. *Malaclemys terrapin* / *Trachemys scripta*	14.6	8.48, 21.8
13. Dermochelyidae / Cheloniidae	75.4	49.4, 104
14. (*Natator depressus* + *Chelonia mydas*) / (*Eretmochelys imbricata* + *Caretta caretta* + *Lepidochelys* sp.)	48	33.7, 61.8
15. *Natator depressus* / *Chelonia mydas*	33.6	33.5, 33.8
16. *Eretmochelys imbricata* / (*Caretta caretta* + *Lepidochelys* sp.)	25.4	17.4, 31.9
17. *Caretta caretta* / *Lepidochelys* sp.	18.6	13.3, 24.7
18. *Lepidochelys olivacea* / *Lepidochelys kempii*	7.72	2.99,12.4

Table S5 - Heterozygosity, inbreeding level (FRoH) and gene density per chromosome for the seven sea turtle reference genomes. This data is provided in a separate file in Excel format.

Table S6 Enrichment p-values for multi-copy gene families in hotspots of genetic diversity

	Chr 13	Chr 14	Chr 24
MHC	0.3785960	8.542079×10^{-26}	1

Immunology	4.126994×10^{-44}	3.892572×10^{-7}	7.524291×10^{-5}
GPCR	2.215344×10^{-49}	1.645942×10^{-3}	5.917677×10^{-5}
Olfactory	2.320270×10^{-81}	2.233347×10^{-10}	0.1254281
Zinc-finger	5.914689×10^{-3}	1.836372×10^{-54}	0.8770295

Table S7 Benjamini-Hochberg-corrected p-values for multi-copy gene families in hotspots of genetic diversity

	Chr 13	Chr 14	Chr 24
MHC	0.4368415	2.562624×10^{-25}	1
Immunology	1.547623×10^{-43}	8.341225×10^{-7}	1.254048×10^{-4}
GPCR	1.107672×10^{-48}	2.468912×10^{-3}	1.109564×10^{-4}
Olfactory	3.480405×10^{-80}	5.583367×10^{-10}	0.1567851
Zinc-finger	8.065485×10^{-3}	1.377279×10^{-53}	0.9396744

Table S8 Weights used to combine transcriptome evidences into a single gene-model set

**Type**	**Evidence**	**Weight**
ABINITIO_PREDICTION	Helixer	1
OTHER_PREDICTION	stringtie_rnaseq	2
OTHER_PREDICTION	stringtie_isoseq	3
OTHER_PREDICTION	toga_rMalTer	4
OTHER_PREDICTION	toga_rCheMyd	5
PROTEIN	rDerCor_miniprot	6
PROTEIN	rCheMyd_miniprot	7

Table S9 Chromosomes and regions included in the PSMC analysis for each sea turtle species. These regions excluded the identified high-diversity regions that may be subject to balancing or diversifying selection, which can bias the demographic inferences.

	Eretmochelys imbricata		*Lepidochelys olivacea*		*Caretta caretta*		*Lepidochelys kempii*		*Natator depressus*	
**Chromosome**	**Start position**	**End position**	**Start position**	**End position**	**Start position**	**End position**	**Start position**	**End position**	**Start position**	**End position**
chr_01	8,975,000	215,025,000	12,175,000	217,225,000	8,975,000	212,725,000	8,025,000	216,125,000	9,075,000	211,075,000
chr_01	223,525,000	361,961,700	218,925,000	355,274,122	218,075,000	355,489,124	217,225,000	354,026,373	213,135,000	349,163,694
chr_02	1	270,895,284	1	272,255,920	1	271,822,458	1	272,641,500	1	265,429,908
chr_03	1	175,725,000	1	212,433,421	6,225,000	212,067,700	1	176,725,000	5,475,000	210,117,355
chr_03	176,325,000	211,952,746	-	-	-	-	177,875,000	212,347,347	-	-
chr_04	4,275,000	146,281,337	7,075,000	150,161,704	11,275,000	154,647,711	1	142,923,576	7,275,000	141,638,353
chr_05	1	135,525,000	1	135,425,000	1	136,775,000	1	136,025,000	1	131,975,000
chr_06	14,775,000	134,836,623	14,475,000	131,075,015	7,825,000	133,850,159	38,025,000	135,739,771	7,025,000	132,879,417
chr_07	1	126,219,764	1	126,826,780	1	127,105,400	1	126,179,207	1	124,409,456
chr_08	1	108,768,616	1	109,072,606	1	109,725,890	1	108,565,403	1	107,029,079
chr_09	1	103,306,587	1	103,789,949	1	103,737,380	1	103,356,104	1	101,916,756
chr_10	1	85,406,469	1	85,838,826	1	85,877,168	1	85,722,145	1	84,161,023
chr_11	1	75,325,000	1	75,675,000	1	75,925,000	1	75,275,000	2,125,000	81,471,429

Table S10 Database and terminology used for classifying genes based on their functional annotation.

**Database**	**Terms used for classifying genes as "MHC"**
Pfam	"MHC class II, alpha chain, N-terminal"
PRINTS	"MHC class I alpha chain, alpha1 alpha2 domains"
PANTHER	"Major Histocompatibility Complex/Immunoglobulin" & "Antigen-presenting and immune regulatory MHC class I-related"
Gene3D	"MHC class II, alpha/beta chain, N-terminal"
FunFam	"Major histocompatibility complex, class I-related protein", "H-2 class I histocompatibility antigen, alpha chain" & "HLA class II histocompatibility antigen, DRB1-1 beta chain"
SMART	"MHC class II, alpha chain, N-terminal"
**Database**	**Terms used for classifying genes as "Immunology-related"**

Pfam	“CD80-like C2-set immunoglobulin domain”, “Immunoglobulin I-set domain”, “Immunoglobulin V-set domain”, “Immunoglobulin domain”, “Immunoreceptor tyrosine-based activation motif”, “Immunoglobulin-like beta-sandwich domain” & “Phosphorylated immunoreceptor signalling ITAM”
SUPERFAMILY	“Immunoglobulin-like domain superfamily”
PANTHER	“Immunoglobulin superfamily BTN/MOG domain-containing protein”, “Immunoglobulin superfamily CEA-related”, “Immunoglobulin-like domain-containing protein LISCH7”, “SIALIC ACID BINDING IMMUNOGLOBULIN-LIKE LECTIN” & “TRANSMEMBRANE AND IMMUNOGLOBULIN DOMAIN-CONTAINING PROTEIN”
Gene3D	“Immunoglobulin-like fold”
SMART	“Immunoglobulin domain subtype”, “Immunoglobulin subtype 2”, “Immunoglobulin V-set domain” & “ Phosphorylated immunoreceptor signalling ITAM”
**Database**	**Terms used for classifying genes as “G-Protein Coupled Receptor (GPCR)”**
Pfam	“G protein-coupled receptor, rhodopsin-like”
PRINTS	“G protein-coupled receptor 40-related receptor” & “G protein-coupled receptor, rhodopsin-like”
SUPERFAMILY	“Family A G protein-coupled receptor-like”
FunFam	“G-protein coupled receptor”
**Database**	**Terms used for classifying genes as “Olfactory Receptor”**
Pfam	“Olfactory receptor”
PRINTS	“Olfactory receptor”
PANTHER	“Human Olfactory Receptors”, “Human and Mouse Olfactory Receptor”, “Olfactory G-protein coupled receptor”, “Olfactory receptor subfamily 6C-like” & “OLFACTORY RECEPTOR”
FunFam	“Olfactory receptor”

Database	Terms used for classifying genes as "Zinc-Finger"
Pfam	"B-box-type zinc finger", "Zinc finger C2H2-type", "Zinc finger, BED-type", "Zinc finger, C3HC4 RING-type", "Zinc finger, CCCH-type", "Zinc finger, PHD-finger", "Zinc finger, RING-type", "Zinc finger, RING-type, eukaryotic", "Zinc finger, TFIIS-type", "Transcription factor S-II (TFIIS) Zinc finger", "Zinc-finger double domain", "zinc finger of C3HC4-type, RING" & "C2H2-type zinc finger"
PRINTS	"Zinc finger, B-box, chordata"
SUPERFAMILY	"B-box zinc-binding domain", "CCCH zinc finger", "FYVE/PHD zinc finger", "beta-beta-alpha zinc fingers"
PANTHER	"ZINC FINGER PROTEIN", "ZINC FINGER AND BTB DOMAIN-CONTAINING", "ZINC FINGER AND SCAN DOMAIN-CONTAINING", "ZNF44 PROTEIN", "Zinc finger BED domain-containing protein", "C2H2-type zinc-finger domain-containing protein" & "Krueppel C2H2-type Zinc Finger"
Gene3D	"Zinc finger, RING/FYVE/PHD-type" & "Classic Zinc Finger"
FunFam	"zinc finger protein", "zinc finger and SCAN domain-containing protein", "Zinc finger with KRAB and SCAN domains", "zinc finger family member", "KRAB zinc finger", "Zinc finger 45-like"
SMART	"B-box-type zinc finger", "Zinc finger C2H2-type", "Zinc finger, CCCH-type", "Zinc finger, PHD-type", "Zinc finger, RING-type" & "Zinc finger, TFIIS-type"
