# Supplementary material for: Haplotype-resolved reference genomes of the sea turtle clade unveil ultra-syntenic genomes with hotspots of divergence
Source: Gigascience. 2025 Sep 18;14:giaf105. doi: 10.1093/gigascience/giaf105 (PMC12448945; doi:10.1093/gigascience/giaf105)
Supplement: giaf105_GIGA-D-25-00103_Revision_1 [file giaf105_giga-d-25-00103_revision_1.pdf]

GigaScience

Haplotype-resolved reference genomes of the sea turtle clade unveil ultra-syntenic genomes with hotspots of divergence

--Manuscript Draft--

Manuscript Number:	GIGA-D-25-00103R1
Full Title:	Haplotype-resolved reference genomes of the sea turtle clade unveil ultra-syntenic genomes with hotspots of divergence
Article Type:	Research
Funding Information:	Revive & Restore (WGM_2021-026) — Dr Camila J Mazzoni<br>NSF-IOS (1904439) — Dr Lisa M Komoroske<br>NOAA Fisheries — Dr Peter H Dutton<br>University of Massachusetts Amherst — Dr Lisa M Komoroske<br>CSIRO's Environomics Future Science Platfor — Dr Oliver Berry
Abstract:	Background: Reference genomes for the entire sea turtle clade have the potential to reveal the genetic basis of traits driving the ecological and phenotypic diversity in these ancient and iconic marine species. Furthermore, these genomic resources can support conservation efforts and deepen our understanding of their unique evolution.<br>Results: We present haplotype-resolved, chromosome-level reference genomes and high-quality gene annotations for five sea turtle species. This completes the catalog of reference genomes of the entire sea turtle clade when combined with our previously published reference genomes. Our analysis reveals remarkable genome synteny and collinearity across all species, despite the clade's origin dating back more than 60 million years. Regions of high interspecific genetic distance and intraspecific genetic diversity are consistently clustered in genomic hotspots, which are enriched with genes coding for immune response proteins, olfactory receptors, zinc fingers, and G-protein-coupled receptors. These hotspot regions may offer insights into the genetic mechanisms driving phenotypic divergence among species, and represent areas of significant adaptive potential. Ancient demographic analysis revealed a synchronous population expansion among sea turtle species during the Pleistocene, with varying magnitudes of demographic change, likely shaped by their diverse ecological adaptations, and biogeographic contexts.<br>Conclusions: Our work provides genomic resources for exploring genetic diversity, evolutionary adaptations, and demographic histories of sea turtles. We outline genomic regions with increased diversity, linked to immune response, sensory evolution, and adaptation to varying environments that have historically been subject to strong diversifying selection, and likely will underpin sea turtle's responses to future environmental change. These reference genomes can assist conservation by providing insights into the demographic and evolutionary processes that sustain and threaten these iconic species.
Corresponding Author:	Camila Mazzoni<br>Leibniz Institute for Zoo and Wildlife Research (IZW) in the Forschungsverbund Berlin eV: Leibniz-Institut fur Zoo- und Wildtierforschung (IZW) im Forschungsverbund Berlin eV<br>GERMANY
Corresponding Author Secondary Information:	
Corresponding Author's Institution:	Leibniz Institute for Zoo and Wildlife Research (IZW) in the Forschungsverbund Berlin eV: Leibniz-Institut fur Zoo- und Wildtierforschung (IZW) im Forschungsverbund Berlin eV
Corresponding Author's Secondary Institution:	
First Author:	Larissa S Arantes

Powered by Editorial Manager® and ProduXion Manager® from Aries Systems Corporation

**First Author Secondary Information:**	
**Order of Authors:**	Larissa S Arantes
	Tom Brown
	Diego De Panis
	Scott D. Whiting
	Erina J. Young
	Erin L. LaCasella
	Gabriella A. Carvajal
	Adam Kennedy
	Deana Edmunds
	Blair P. Bentley
	Jennifer Balacco
	Conor Whelan
	Nivesh Jain
	Tatiana Tilley
	Brian O'Toole
	Patrick Traore
	Erich D. Jarvis
	Oliver Berry
	Peter H Dutton
	Lisa M Komoroske
	Camila J Mazzoni
**Order of Authors Secondary Information:**	
**Response to Reviewers:**	Reviewer #1:<br>7. Conclusion<br>The inferential statistics for the objective "Investigation of multi-copy gene family enrichment in genomic hotspots of sea turtles" were successfully reproduced using the original analysis code provided by the authors. The input data needed to run the code were initially unavailable but were subsequently shared through the Git repository. An inconsistency was noted in the text of the manuscript reporting a threshold for Olfactory Receptors, where the stated 10^-10 should be revised to 10^-9 based on the observed p-value (5.583367e-10).<br>Thank you for the time taken to thoroughly review our scripts and test their reproducibility. We have updated our human-based rounding error for the Olfactory Receptors in the manuscript (line 234) and incorporated the comments below into both the README file and R script. Particularly, we have made clearer in the README which files or results are used as the input for each script and detailed which Figure was generated using each script.<br><br>- Recommendations for authors<br>While the original analysis code was successfully used to reproduce the results, we recommend improving the documentation to enhance clarity and reproducibility. In particular:<br>-- Code annotation: The scripts would benefit from more detailed comments within the code to clarify the logic of each step. This would greatly help users follow the analyses more easily and understand the purpose of specific commands or operations.<br>-- README file: The current README provides only a general overview. We suggest expanding it to include:<br>--- A brief description of each script or analysis pipeline.<br>--- An indication of which figure, table, or result in the manuscript each script

Powered by Editorial Manager® and ProduXion Manager® from Aries Systems Corporation

	corresponds to.<br>--- Clear instructions on how to execute the analyses in the correct order, if applicable.<br>-- Metadata: For the datasets used or generated by the scripts, it would be helpful to include accompanying metadata files that explain:<br>--- The definition of each variable name.<br>--- The origin of each dataset (raw, processed, etc).<br>--- Any preprocessing steps applied before analysis.<br>-- Data availability: At this stage, we have only verified the reproducibility of one part of the study. To facilitate full reproducibility of the entire study, we recommend sharing all necessary data files required to run every script present in the repository.<br><br>We have incorporated the above comments into the gitlab repository https://git.imp.fu-berlin.de/begendiv/sea_turtlegenomes including the edits made directly by the reviewer. Many thanks again.<br><br>These improvements would make the repository significantly more user-friendly and would strengthen the reproducibility of the study.<br><br>Reviewer #2: The authors of this work provide a fantastic addition to the genomic resources currently available for marine turtles with five new, apparently high-quality reference genomes. These new resources enable a number of interesting cross-species analyses in this group, including phylogenetic reconstruction, inference of demographic history, and identification of hotspots of diversity and divergence. I though this paper was quite clearly written and easy to read overall, and I have one major and a few more minor comments/suggestions.<br>We appreciate the reviewer's positive feedback and thoughtful comments.<br><br>Major comment: there is an extensive literature on hybridization among marine turtle lineages (see Vilaca et al. 2021, https://doi.org/10.1111/mec.16113, for a recent genomic example), with lots of evidence for ancient gene flow after initial lineage divergence as well as recent hybridization. The authors do not really mention this phenomenon at all, and since I think it has a lot of bearing on all of the results it would make sense to re-think your findings in light of the fact that some level of gene flow has occurred. Would extensive synteny/lack of genomic rearrangements potentially enable hybridization? Is overall low divergence among lineages potentially a function of gene flow? Are regions of high divergence the result of selection (as you suggest), or could these regions potentially be resistant to gene flow? I believe that IQtree assumes a strictly bifurcating tree, and gene flow can influence PSMC inferences (see Mazet et al. 2016, https://doi.org/10.1038/hdy.2015.104) - how would gene flow among lineages affect your inference of divergence dates and demographic histories?<br>We thank the reviewer for this insightful comment. Indeed, hybridization has played a significant role in the evolutionary history of sea turtles, with extensive evidence for both ancient gene flow following lineage divergence and ongoing hybridization events. As the reviewer correctly points out, such gene flow can influence divergence time estimates derived from nuclear genomes, often resulting in estimates that appear more recent due to post-divergence gene flow. Despite potential bias, the bifurcating tree model used by MCMCtree provides a widely accepted approximation of the major split events among sea turtle lineages. In response to this suggestion, we have incorporated a comment of this phenomenon into the revised manuscript, specifically in the context of divergence time estimation. We have also added a paragraph speculating how the conservation of gene order may have allowed hybridisation to happen along the evolution of sea turtle lineages. We have added a mitigating statement to this effect in lines 217-221<br><br>MInor commentsL [note - line numbers would have been helpful for providing comments on specific items! I will refer to the lower-left page numbers and paragraph instead]:<br>Apologies for the oversight, we have added line numbers to this submission and referenced the lines in our responses.<br><br>page 3, paragraph 2: Some of the applications you refer to here don't seem terribly germane to the relevance of "genomic resources" in management and conservation per se, and several are just methods using some kind of genetic data ... e.g., "abundance"/close-kin mark recapture doesn't require full genomes (and the reference

Powered by Editorial Manager® and ProduXion Manager® from Aries Systems Corporation

	you cite used microsat data), and the "community"/eDNA applications don't generally rely on genomes but instead on databases of a few (usually mitochondrial) genes. Either include methods that truly benefit from the development of high-quality reference genomes or broaden this to something like "growth in molecular ecology techniques". We appreciate the feedback that we have not highlighted the necessity of highly-accurate, contiguous, chromosome-level assemblies in conservation management, as was also highlighted by review 3 below. We have tried to better highlight how the increased contiguity and scaffolding in particular helps accurate inference of features of the genome architecture itself, such as detecting Runs of Homozygosity, necessary for much of our analysis here. The main strength lies in the “catch-all” nature of these assemblies, allowing not only investigation of the chromosome structure, but also those features possible with fragmented, or draft, assemblies such as gene evolution and overall heterozygosity. We have added sentences to this effect in the introduction at lines 81-91.<br><br>page 4, paragraph 2: last sentence is a bit of a run-on, could break this up a bit. We have split and shortened this sentence in lines 114-115 to hopefully make this section more readable<br><br>page 10, paragraph 3: for me, the ROH methods need some additional explanation and interpretation. The more detailed methods indicate that the ROH were identified on the basis of lower-than-average heterozygosity rather than true homozygosity - I can understand why this might have been done (since the baseline level of heterozygosity varies across species) but it still seems a bit arbitrary and could risk mistaking stretches with simply low variation for IBD tracts. I wonder if a ROH-detection method like ROHan that explicitly incorporates baseline genomic heterozygosity into its model would be more appropriate for comparing results across species and could give different results.<br><br>We appreciate the reviewer's comments on ROH detection methods. We chose Darwindow specifically because it can perform the analysis using a single individual. While ROHan would indeed explicitly incorporate baseline heterozygosity, it cannot process long-read data, making it unsuitable for our dataset.<br>To address concerns about method consistency, we show here a validation using a sea turtle species (Chelonia mydas) for which we have both a high-quality genome assembly and short-read data (GCA_015237465.2; SRS18701202). We ran both ROHan and Darwindow on this dataset using comparable parameters (50 Kbp windows) and found similar ROH patterns between the two methods (see below), suggesting that Darwindow's approach of identifying regions with lower-than-average heterozygosity produces comparable results to ROHan's model-based method .<br><br>In the above comparative analysis of the C. mydas reference genome (GCA_015237465.2), using chromosome 3 (NC_057851.1) as an example, we show that depressions in heterozygosity detected by ROHan generally coincide with ROH segments identified by Darwindow. In panel (A), ROHan results are visualized: green represents point estimates, while magenta and red indicate the upper and lower bounds of local heterozygosity rates, respectively. In panel (B), ROH segments identified by Darwindow are shown as red boxes overlaid on heterozygosity levels (black lines).<br>Genome-wide, the heterozygosity estimate from ROHan is 0.28% (mid-value), with 12.93% of the genome identified as ROH. For Darwindow, these values are 0.21% and 18.63%, respectively. These results align with those reported by Bentley et al. (2023) for the same individual and dataset, but using PLINK, which estimated a ROH% of 17.7.<br>To allow a better comparison with the previous PLINK result, ROHan was run with parameters --size 50000 and --rohmu 2e-5. Darwindow was run with WIN_SIZE="10000", NUM_WIN="5", and MAX_MISS="0.4". Bentley et al. (2023) ran PLINK with the --homozyg option using the parameters: --homozyg-kb 50, --homozyg-snp 20, --homozyg-window-missing 5, and --homozyg-window-het 1.<br><br>I also question a bit the interpretation of these low-diversity tracts as evidence of

Powered by Editorial Manager® and ProduXion Manager® from Aries Systems Corporation

inbreeding per se. The authors do not comment much on the length distributions of these ROH - given that many of them are quite short I would expect that if there was mating between close kin it probably happened
far back in the past and the IBD tracts have been broken up by recombination.
The reviewer makes an important point and is correct that the length distribution of ROH is crucial for distinguishing between ancient demographic processes and recent population restrictions. Although our analysis already included ROH length distributions (Fig 5a), we had not properly incorporated these results into our manuscript. We have now revised the text to better describe the ROH length patterns and provide more nuanced interpretations. We thank the reviewer for this comment as it helps improve our manuscript. We have extensively revised the text in section 3.4 to make it clearer the implications of the lengths of the detected ROHs, lines 254-267

page 11, paragraph 2: for PSMC analyses it is important to note the method assumes that differences in coalescence time/Ne across the genome result from demography alone. If portions of the genome are under balancing/diversifying selection (such as the areas of high diversity that you detect in this study), the local Ne for inferred these regions would be expected to be larger than the rest of the genome, which could lead to the spurious detection of population expansion or contraction (more likely a contraction for balancing selection). See Boitard et al. 2022 (https://doi.org/10.1093/genetics/iyac008) for a more detailed treatment. I would try excluding the regions putatively under diversifying selection and re-run PSMC to see if your inferences change.
We agree that regions under balancing or diversifying selection can bias demographic inferences based on PSMC. To address this concern, we conducted an additional PSMC analysis using data exclusively from the 11 macrochromosomes, excluding the identified high-diversity regions that may be subject to balancing or diversifying selection. The resulting demographic trajectory was highly consistent with our original findings. This likely reflects the fact that many of the high diversity regions are actually repetitive elements that were already masked during the genome preprocessing, and thus excluded from the original PSMC analysis. We have now included this additional analysis and its implications in the revised manuscript, which can be found in Figure S14 (lines 281-286 and 622-625).

Reviewer #3: (1)It is recommended to add keywords such as "conservation genomics" or "adaptive evolution" to better align with the content.
Thank you for your suggestion. We have added both keywords.

(2)In the background section, after discussing the current status of sea turtles and existing genomic research, the study's content is introduced directly without adequately explaining why it is necessary to sequence the genomes of the remaining five species of sea turtles on top of the existing partial genomic data. The introduction of the research objectives appears somewhat abrupt.
Thank you for highlighting our oversight, which was also raised by reviewer 2. As above, we have added sentences to highlight the improved usefulness of highly contiguous, accurate and chromosome-level assemblies for conservation and other management strategies in lines 81-91.

(3)Last line of page four"……within this ancient clade [34,38]"：When introducing the broad context of genomics and biodiversity conservation, it is important to provide detailed explanations for key concepts such as 'genomic synteny' and 'colinearity'. Although these concepts are covered later in the analysis of the turtle genome, providing initial elaboration can help readers better understand subsequent content.
Thank you for the suggestion. We have added a short definition covering synteny and collinearity to the introduction (Lines 121-123) that we hope will make these concepts clearer to the reader.

(4)Page 6 Section 2.2:The range of this quality value, 38.7, is incorrect. Please verify carefully.
Apologies for the confusion in listing this value. We meant to only highlight the “primary” assemblies and have made this clearer in the text, noting the species from which each value derives and that we restrict this to the primary assembly in each case in lines 163-165.

Powered by Editorial Manager® and ProduXion Manager® from Aries Systems Corporation

	(5)Result 3.1：High conservation at the chromosomal level is supported, but repetitive sequences must be excluded from synteny analysis.<br>We agree entirely with this statement and have tried to ensure this is clearer in our methods and results sections. Syntenic comparisons in section 3.1 were performed based on identified protein orthologs, so would not include repetitive sequences. We have added a statement to this effect in section 3.1 and made this clearer in the methods section 6.5. We have also made it clearer in methods section 6.7 that these repetitive regions were also masked before alignments were performed using cactus in order to calculate genetic distances between species.<br><br>(6)Section 3.4, Second Paragraph：The reliability of PSMC in low-diversity species, such as N. depressus, may be limited; it is recommended to validate findings with other methods, such as MSMC2.<br>We agree that validating demographic reconstructions through multiple complementary approaches enhances their robustness. To this end, we applied MSMC2 to the seven sea turtle reference genomes. The resulting effective population size trajectories closely mirrored those inferred from PSMC analyses, providing strong cross-validation of the demographic patterns observed (Figure S15).<br>While PSMC and MSMC2 relies on the distribution of heterozygous sites across the genome to reconstruct historical effective population size trajectories, to our knowledge, no study has formally evaluated or systematically quantified how reduced heterozygosity in low-diversity species impacts the resolution and reliability of demographic inferences.<br>To mitigate our demographic analysis limitations, we have taken careful steps to assess the reliability of our PSMC and MSMC2 inferences by (i) evaluating the robustness of their results through bootstrap analyses, and (ii) excluding microchromosomes and regions of high genetic diversity in the 11 macrochromosomes (Figure S14), which are potentially under selection, from the PSMC analysis. Additionally, we have discussed the limitations of generalizing the demographic history of an entire species based on a single genome (Lines 357-363).<br><br>(7)It is recommended to include a detailed description of sample selection in the methods section, covering aspects such as geographic distribution, population size, and sample collection methods, to demonstrate the representativeness and reliability of the selected samples.<br>We have added further details to the manuscript in section 6.1 on the origin of these individuals and the current Regional Management Units responsible in each case.
**Additional Information:**	
**Question**	**Response**
Are you submitting this manuscript to a special series or article collection?	No
**Experimental design and statistics**<br><br>Full details of the experimental design and statistical methods used should be given in the Methods section, as detailed in our Minimum Standards Reporting Checklist. Information essential to interpreting the data presented should be made available in the figure legends.<br><br>Have you included all the information requested in your manuscript?	Yes
**Resources**	Yes

Powered by Editorial Manager® and ProduXion Manager® from Aries Systems Corporation

A description of all resources used, including antibodies, cell lines, animals and software tools, with enough information to allow them to be uniquely identified, should be included in the Methods section. Authors are strongly encouraged to cite Research Resource Identifiers (RRIDs) for antibodies, model organisms and tools, where possible.<br><br>Have you included the information requested as detailed in our Minimum Standards Reporting Checklist?	
**Availability of data and materials**<br><br>All datasets and code on which the conclusions of the paper rely must be either included in your submission or deposited in publicly available repositories (where available and ethically appropriate), referencing such data using a unique identifier in the references and in the "Availability of Data and Materials" section of your manuscript.<br><br>Have you have met the above requirement as detailed in our Minimum Standards Reporting Checklist?	Yes
GigaScience has policies and guidelines in place for the use of generative AI-writing tools such as ChatGPT. If you have used such writing tools to assist with writing the manuscript this must be declared and cited in the text. Authors should not list AI-writing tools and other AI-assisted technologies as an author or co-author and should acknowledge that they are fully responsible for text generated or refined by AI-writing tools.<p><br><br>A summary of use (particularly in the introduction or among methods) needs to be included at the end of the paper, and the outputs should also be included as a supplementary file hosted in GigaDB or other open repositories. Please <a href=https://academic.oup.com/gigascienc	Yes

e/pages/editorial_policies_and_reporting_standards target="_new" > read our guidelines for more information. <p><br><br>By submitting to GigaScience, you are aware of the journal's AI-writing tools policy, and if you have declared use of such tools below, you have acknowledged this where appropriate in your manuscript and have made a summary of use and outputs available. <p><br><b>AI-assisted writing tools have been used in the preparation of this manuscript?	

Manuscript Click here to access/download;Manuscript;Turtle text - Revision 1.pdf

Haplotype-resolved reference genomes of the sea turtle clade unveil ultra-syntenic genomes with hotspots of divergence

Larissa S. Arantes[1,2]*, Tom Brown[1,2]*, Diego De Panis[1,2]*, Scott D. Whiting[3], Erina J. Young[4], Erin L. LaCasella[5], Gabriella A. Carvajal[6], Adam Kennedy[7], Deana Edmunds[8], Blair P. Bentley[9], Jennifer Balacco[10], Conor Whelan[10], Nivesh Jain[10], Tatiana Tilley[10], Brian O'Toole[10], Patrick Traore[10], Erich D. Jarvis[10], Oliver Berry[11], Peter H. Dutton[5], Lisa M. Komoroske[12], Camila J. Mazzoni[1,2]✉

[1] Department of Evolutionary Genetics, Leibniz Institute for Zoo- and Wildlife Research (IZW), Berlin, Germany
[2] Berlin Center for Genomics in Biodiversity Research (BeGenDiv), Berlin, Germany
[3] Marine Science Program, Department of Biodiversity, Conservation and Attractions, Kensington, WA 6151, Australia
[4] Conservation Medicine Program, School of Veterinary Medicine, Murdoch University, Murdoch, WA 6150, Australia
[5] Marine Mammal and Turtle Division, Southwest Fisheries Science Center, National Marine Fisheries Service, National Oceanic and Atmospheric Administration, La Jolla, CA, United States
[6] Department of Biological Sciences, Florida Atlantic University, FL 33431, Florida, USA
[7] New England Aquarium Rescue and Rehabilitation Department, Quincy, MA, USA
[8] New England Aquarium Animal Health Department, Quincy, MA, USA
[9] Department of Biological Sciences, Smith College, Northampton MA 01060 USA
[10] Vertebrate Genome Laboratory, The Rockefeller University, NY, USA
[11] CSIRO Environomics Future Science Platform, Indian Ocean Marine Research Centre, Crawley, Western Australia, 6009, Australia
[12] University of Massachusetts Amherst, Department of Environmental Conservation, Amherst, MA, USA

* These authors contributed equally to this work.
✉ Corresponding author.

Keywords

Cheloniidae, Reference Genomes, Conservation Genomics, Adaptive Evolution, Genetic Diversity, Demography, Synteny

Abstract

Background: Reference genomes for the entire sea turtle clade have the potential to reveal the genetic basis of traits driving the ecological and phenotypic diversity in these ancient and iconic marine species. Furthermore, these genomic resources can support conservation efforts and deepen our understanding of their unique evolution.

Results: We present haplotype-resolved, chromosome-level reference genomes and high-quality gene annotations for five sea turtle species. This completes the catalog of reference genomes of the entire sea turtle clade when combined with our previously published reference genomes. Our analysis reveals remarkable genome synteny and collinearity across all species, despite the clade's origin dating back more than 60 million years. Regions of high interspecific genetic distance and intraspecific genetic diversity are consistently clustered in genomic hotspots, which are enriched with genes coding for immune response proteins, olfactory receptors, zinc fingers, and G-protein-coupled receptors. These hotspot regions may offer insights into the genetic mechanisms driving phenotypic divergence among species, and represent areas of significant adaptive potential. Ancient demographic analysis revealed a synchronous population expansion among sea turtle species during the Pleistocene, with varying magnitudes of demographic change, likely shaped by their diverse ecological adaptations, and biogeographic contexts.

Conclusions: Our work provides genomic resources for exploring genetic diversity, evolutionary adaptations, and demographic histories of sea turtles. We outline genomic regions with increased diversity, linked to immune response, sensory evolution, and

adaptation to varying environments that have historically been subject to strong diversifying selection, and likely will underpin sea turtle's responses to future environmental change. These reference genomes can assist conservation by providing insights into the demographic and evolutionary processes that sustain and threaten these iconic species.

1. Background

The rapid loss of biodiversity, driven by erosion and destruction of habitats globally, underscores the urgent need to develop strategies to mitigate this crisis and safeguard the planet's ecological balance, reversing declines in biodiversity. One of the fastest growing technologies for understanding biodiversity and supporting its management is genomics [1]. Recent advances in high-quality genomic resources have facilitated our abilities to explore the genetic underpinnings of Earth's biodiversity, enabling a deeper understanding of the evolutionary and functional complexities of life. Initiatives such as the Earth Biogenome Project [2], European Reference Genome Atlas [3], Darwin Tree of Life [4] and Vertebrate Genomes Project [5] have driven standards and recommendations for the production of high-quality reference genomes for conservation of biodiversity. These initiatives have resulted in an ever growing database of high-quality reference genomes, which is expanding rapidly as technologies evolve.

This growth in genomic resources has allowed researchers to investigate the genetic bases of a number of features key to assisting in species management and conservation such as age and lifespan [6,7], sex [8], abundance [9] and community composition [10] among others. Focussing on the evolutionary adaptations of iconic or umbrella species

within ecosystems gives us the opportunity to efficiently monitor biodiversity and assess the health of these ecosystems [11,12]. Anchoring such analyses to high-quality, chromosome-level reference genomes allows for a much more comprehensive interrogation of genomic architecture. This is largely due to the improved contiguity of these assemblies, which facilitates the resolution of complex genomic features such as multigenic regions potentially under selection, repeat-rich areas, and large-scale structural variants [13]. Transitioning from fragmented draft assemblies to highly contiguous genomes also enhances the detection of long runs of homozygosity (ROHs), offering critical insights into recent inbreeding and population history [14]. Finally, contiguous, accurate, chromosome-level assemblies such as those presented here allows us to investigate all of these features using one reference, which is not possible with fragmented or scaffold-level assemblies.

Sea turtles have existed since non-bird dinosaurs were roaming the Earth [15] and hold critical ecological roles in both oceanic and coastal environments, but are at threat globally due to anthropogenic activities such as direct harvest, fisheries bycatch, habitat loss and climate change, among other risks [16–18]. At present, three of the seven extant sea turtle species have been classified under IUCN criteria as ‘endangered’ (*Chelonia mydas* [19]) or ‘critically endangered’ (*Eretmochelys imbricata* and *Lepidochelys kempii* [20,21]) and a further three (*Lepidochelys olivacea*, *Caretta caretta* and *Dermochelys coriacea* [22–24]) have been classified as ‘vulnerable’. Finally, while listed as ‘data deficient’ under the IUCN Red List, *Natator depressus* [25] has been classified as ‘vulnerable’ by the Australian government [26]. Extensive conservation efforts have led

to positive outcomes for many populations [27], however effort and success have not been universal, with some populations still in decline [28].

Sea turtle species exist around the globe, inhabiting a remarkable diversity of ecological niches [29], spanning from deep cold-water oceanic divers like *D. coriacea* to range-restricted endemic species, such as *N. depressus* and *L. kempii* [30]. For other species, their habitats span the tropics and sub-tropics (*L. olivacea)* and broader, temperate and tropical ranges, such as *C. mydas*, *E. imbricata*, and *C. caretta*. Some sea turtles demonstrate dietary specializations (*D. coriacea* and *E. imbricata*), while others (e.g. *C. mydas* and *C. caretta)* display generalist omnivorous feeding habits [31]. The genomic bases for these traits remain unclear, however having access to high-quality genomic resources would allow more fine-level investigation into genetic drivers behind the capabilities of sea turtles to live in varying habitats and adapt to changing conditions in the Anthropocene [32–34]. Annotated, high-quality reference genomes for each species allow for investigation into areas such as identifying genes under selection, or areas of adaptive potential [13].

At present, genomes are available for five of the seven extant sea turtle species, namely from *C. mydas* and *D. coriacea* [14], *C. caretta* [35]*, E. imbricata* [36] and *L. olivacea* [37]. Previous analyses in particular of the genomes of *C. mydas* and *D. coriacea* that represent the two extant sea turtle families *(Dermochelyidae and Cheloniidae)* have revealed a high degree of synteny and collinearity, defined as blocks of the genome with shared arrangement and orientation of genomic features, such as genes or other aligned elements, within this ancient clade [14,38]. Alongside this high level of apparent genomic conservation, small highly divergent genomic regions have also been observed between

these two species, in particular in areas containing multi-copy gene families such as the Major Histocompatibility Complex (MHC) and olfactory receptors [14] as well as some rearrangement of genes potentially involved in temperature-dependent sex determination [38]. While these are clearly important genomic regions for understanding sea turtle adaptation and evolution, it is not clear if the differences between *C. mydas* and *D. coriacea* are species specific, or how well they characterize comparative patterns within the entire sea turtle clade.

In this study, we add to our previous reference genomes for *C. mydas* and *D. coriacea* [14], by producing high-quality genomes for the remaining five extant sea turtle species. Our genomes are assembled using highly accurate PacBio HiFi (High-Fidelity) and Chromatin-Conformation-Capture (Hi-C) sequencing, producing genomes with chromosomes phased into both parental haplotypes. This first full catalogue of sea turtle genomes now provides a unique opportunity to understand and investigate the evolution of sea turtles and contextualise their evolution among other turtles and tortoises, spanning hundreds of millions of years of evolution. We uncover high levels of genome-wide synteny across all Testudine genomes, with a notable pattern of genetic diversity and divergence within the sea turtle clade, intricately clustered within specific regions of specific chromosomes. These regions are enriched in immune-related genes, suggesting a role in the adaptive capabilities of these species. Furthermore, we performed demographic analysis, calculated genetic diversity, and identified ROHs in the genome to provide deeper insights for conservation efforts. Thus, we demonstrate the power of high-quality genomes to uncover complex patterns of genetic diversity and adaptation that are vital for understanding species evolution and guiding conservation strategies.

2. Data Description

2.1. Sequencing

For the five turtle species (*C. caretta*, *E. imbricata*, *L. olivacea*, *L. kempii*, *N. depressus*), we sequenced PacBio HiFi reads ranging from 35x to 60x coverage for each genome (Fig S1) and Hi-C sequences ranging from 47x to 129x coverage. We sequenced optical maps with N50 values ranging from 222 to 266 kbp and total DNA yields from 103 to 498 Gbp for long-range molecules of higher quality for 3 of the five species (*L. olivacea*, *C. caretta* and *E. imbricata* Table S1). These datasets are available via the European Nucleotide Archive (ENA) and National Centre for Biotechnology Information (see Data Availability).

2.2. Genome Assembly

Our haplotype-separated chromosome-scale assemblies are highly contiguous, and in particular, are significantly more contiguous than the previously published *D. coriacea* assembly based on PacBio CLR data and *C. caretta* assembly based on Oxford Nanopore Technology (ONT) reads (Figs 1a, S1 & S2, Table S2). Moreover, the new haplotype assemblies show exceptional base accuracy, with a Quality Value (QV) ranging from 65.2 to 70.4 (Table S2). For comparison, the older CLR-based primary assemblies display QVs in the range of 38.7 (*D. coriacea)* to 47.6 (*C. mydas*), while the ONT-based *C. caretta assembly* is much lower (in part due to a different sample used for analysis, Table S2). All genomes were scaffolded into complete chromosome molecules, with between 99.1% and 99.9% of the assembled sequences assigned to the 28 chromosomes (Figs 1b, S3 & S4, Table S2). The assembled genomes also show high

gene completeness, with between 98.5% and 99.5% single-copy orthologs from the Sauropsida lineage identified by BUSCO (Figs 1c & S5, Table S2).

2.3. Genome Annotation

Using a combination of approaches based on transcriptomic data, protein sequences from *C. mydas* and *D. coriacea*, liftover annotations from *C. mydas* and *Malaclemys terrapin pileata,* as well as *de-novo* gene predictions, we created a set of protein-coding gene predictions for each of our assembled genomes (Table S3). The annotations themselves are highly complete when evaluated based on single-copy orthologs from Sauropsida via BUSCO (Fig 1d) and hierarchical orthology groups from Archelosauria via OMArk (Fig S6), reaching comparable completeness to previous annotations generated by RefSeq, with BUSCO scores between 97.2% and 98.1% and OMArk completeness scores between 97.46% and 98.47%, furthermore capturing many BUSCO genes missing in the annotation provided for the existing *E. imbricata* reference genome [36].

3 Analyses

3.1 Genome Synteny

Based on identification of orthologous genes and their locations in Testudine genomes, we uncovered remarkably high synteny across the clade, encompassing over 100 million years of evolution with only a small number of hotspots of variation identified among the unique-sequence regions of the genomes. Particularly among the sea turtles, all 28 chromosomes were highly collinear and syntenic (Figs 2a & S7) with complete one-to-one synteny demonstrated at the chromosome level, with the exception of one region at

the end of chromosome 14 in *D. coriacea*, found in chromosome 11 in the six *Cheloniidae* turtle species (Fig S7).

Across Testudine genomes (i.e. including terrestrial and freshwater turtle and tortoise families; Fig 2), we found that the macrochromosomes (>50Mb in length) exhibited high synteny across all turtle genomes and among the microchromosomes (<50Mb in length), with only chromosomes 21 and 26 from sea turtles rearranged in other turtle genomes. In these instances, chromosomes 21 and 26 from the sea turtle genomes were found in the arm of chromosome 4 (which is syntenic to chromosome 6 in the sea turtle genomes), and the central region of chromosome 2, respectively, in the genome of the Chinese pond turtle (*Mauremys reevesii)* with this positioning conserved across all other Testudine genomes (Figs 2a, S8, S12 & S13).

3.2 Phylogenomic analysis

Phylogenetic analysis using coding-protein sequences for all turtle species with annotation available provided insights into evolutionary relationships and speciation events within suborder Cryptodira, which includes most living turtles and tortoises (Fig 2b). The topology and divergence time support the findings of previous studies based on a few nuclear markers or mitochondrial DNA [39–41] (Table S4). Our genome-wide analysis indicates that the sea turtle clade diverged from other Durocryptodira species 104 million years ago (mya) [95% highest posterior density (HPD) = 81.9 to 122 mya]. Dermochelyidae (including *D. coriacea*) separated from the Cheloniidae family approximately 75.4 mya (95% HPD = 49.4, 104). Within the Cheloniidae family, the divergence of *C. mydas* and *N. depressus* occurred approximately 33.6 mya (95% HPD

= 33.5, 33.8), while the other species diverged around 25.4 mya (95% HPD = 17.4, 31.9). *Lepidochelys kempii* and *L. olivacea* were the most recently diverged lineages, having split around 7.72 mya (95% HPD = 2.99, 12.4), a time period associated with significant environmental changes such as the closure of the Tethys Sea and cooling of the southern oceans, which likely disrupted gene flow and contributed to the speciation of these two *Lepidochelys* species [40,42]. We acknowledge that the MCMCtree method used to estimate divergence times assumes a strictly bifurcating tree and does not account for post-divergence gene flow. This limitation is particularly relevant given the well-documented history of hybridization among sea turtle species [43], which can lead to underestimated divergence times when gene flow occurs after initial lineage splitting.

3.3 Genome-wide patterns of diversity and divergence

Comparisons of within-individual genetic diversity, measured by average heterozygosity per chromosome, revealed consistent variation across chromosomes in the individuals sequenced, with each species showing distinct magnitude of variation (Figs 3a & S9). Notably, chromosomes with high intraspecific diversity also exhibited higher gene density (Fig 3b) and increased interspecific genetic distance (Fig 3c). Consistent with findings in sea turtles and other species with microchromosomes [44], we found that the average heterozygosity, as well as the gene density and interspecific genetic distance, were higher for microchromosomes (12-28) than macrochromosomes (1-11) ($p<0.05$) (Figs 3a-c). In particular, chromosomes 13, 14, 20, 23, 24, and 28 exhibited heightened genetic diversity, interspecific divergence, and gene density (Figs 3a, 3c, S9 & S10, Table S5).

Furthermore, increased levels of heterozygosity and interspecific genetic distance were concentrated at particular hotspot regions, defined as regions with heterozygosity exceeding four times the chromosomal mean and genetic distance double that of the chromosomal mean, rather than being uniformly distributed across an entire chromosome (Fig S9). Thus, we identified the three regions located in chromosomes 13, 14 and 24 exhibiting colocalised elevations in heterozygosity and genetic distance across sea turtles (Fig 4). While we identified these hotspot regions by calculating genetic distances from all species in relation to *D. coriacea* (Fig 4b), this pattern is consistent across pairwise comparisons between all species (Figs S10 & S11).

Following functional annotation of the genes found in these hotspots, we found enrichment for multi-copy gene families coding for proteins with functions in immune response, olfactory receptors (ORs), zinc fingers, and G-protein-coupled receptors (GPCRs) (Fig 4c, Tables S6 & S7). This included enrichment of immunology-related genes, GPCRs, ORs, and Zinc-finger genes in chromosome 13 (adjusted $p < 10^{-42}$, 10^{-47}, 10^{-79}, 0.01, respectively), MHC genes, Immunology-related genes, GPCRs, ORs, and Zinc-finger genes in chromosome 14 (adjusted $p < 10^{-24}$, 10^{-6}, 10^{-2}, 10^{-9}, $10^{-52,}$ respectively) and Immunology-related genes and GPCRs in chromosome 24 (adjusted $p < 10^{-3}$ and 10^{-3}, respectively). A particular concentration of olfactory receptors - known for their role in odor perception and detection of chemical cues, was identified in the hotspot region of chromosome 13 (Fig 4) and Major histocompatibility complex (MHC) genes were concentrated within the identified hotspot on chromosome 14.

3.4 Homozygosity Patterns and Historical Demography

We analysed the proportion of the genome in ROHs (FROH) for each species and categorised segments by length to distinguish between ancient demographic events that resulted in background relatedness (short ROH) and recent consanguinity (long ROH) [45]. The *N. depressus* individual had the highest overall FROH (0.227), predominantly comprising short (0.5-1 Mb) segments but with substantial representation in longer categories (1-2 and 2-5 Mb. Fig 5a). This distribution suggests its elevated homozygosity results from a combination of ancient demographic processes and more recent population declines. In contrast, *L. olivacea* individual showed the lowest FROH (0.0149), consisting mainly of short ROH, indicating a historically larger and more stable population. The reference *C. mydas* individual, despite moderate total FROH, showed a higher proportion of long ROH segments (Fig 5a, Fig S9). This pattern is consistent with Bentley et al. [14], as this individual originates from a small breeding population in the Mediterranean sea where recent shared ancestry between maternal and paternal lineages is more likely [45].

Using Pairwise Sequentially Markovian Coalescent (PSMC) models [46], we reconstructed the demographic histories of the seven extant sea turtle species, revealing consistent patterns of population declines beginning approximately 1–9 mya, likely driven by cooler sea temperatures (Fig 5b). During the Mid-Pleistocene Transition, between 500 kya and 1.2 mya, population decline ceased and all species, except *L. kempii*, began to experience synchronous population expansions, with the growth particularly pronounced in *C. mydas*, *C. caretta* and *E. imbricata*, while *L. kempii* maintained a relatively stable population size. The population peak occurred between 300 kya and the Last Interglacial in the Eemian period (130-115 kya). This period of growth was followed by a second

population decline across all species, starting roughly 100 kya until recently around 50 kya. The three species with relatively stable historical population sizes - *N. depressus*, *L. kempii*, and *L. olivacea* - differ significantly in their levels of genetic diversity. *N. depressus* exhibits the lowest heterozygosity, while the two *Lepidochelys* species display relatively high heterozygosity. These results were found to be robust when considering only the largest 11 chromosomes (macrochromosomes, Fig S14), removing their regions with elevated levels of heterozygosity and genetic diversity (Fig 3, Table S9) to minimize potential confounding effects of selection. Demographic trajectories were further inferred using an independent method, MSMC2, which produced broadly consistent demographic trajectories and effective population sizes as those obtained via PSMC (Fig S15).

4. Discussion

Our chromosome-scale, annotated genomes across the sea turtle clade revealed remarkable genetic synteny across this slowly-evolving group of animals, while also revealing hotspot regions of the genome consistently undergoing accelerated evolution and divergence that likely play important roles in the morphological and ecological diversity exhibited among these species. These regions contained genes important for immune responses, the ability to sense and respond to the environment and regulate gene expression under fluctuating environmental conditions [47]. This builds on previous results comparing genomes of *C. mydas* and *D. coriacea* [14], demonstrating that sea turtle genomes have remained highly syntenic since their split from freshwater turtles and tortoises around 100 million years ago.

Our findings of ultra-synteny across the high-quality genomes of all seven extant sea turtle species reveals a striking conservation of chromosomal architecture that may underlie the known hybridisation among sea turtles observed between ancient [43] and recent [48] species divergence. This structural stability may have supported ancient hybridisation events via preserved gene order and structure, facilitating chromosome-pairing during meiosis, reducing incompatibilities, and enabling the formation of viable and fertile hybrids [49].

The availability of high-quality reference genomes for all sea turtles opens new avenues to explore fundamental questions about their adaptation, immunity, and sensory evolution. Sea turtles exhibit remarkable adaptations to marine environments, including extreme migratory behaviors [50], saltwater tolerance [51], natal homing [52,53], and temperature-dependent sex determination [54], yet the genetic basis of these traits remains poorly understood. Our results highlight microchromosomes and specific regions of reduced relative synteny in macrochromosomes as key loci enriched in gene density and genetic variation across the sea turtle clade. This pattern is also observed in birds and other reptiles, with the high GC content and high recombination rate of the microchromosomes potentially playing a significant role in promoting diversification [44].

The highlighted hotspots of evolutionary diversification harbor multicopy gene families, such as olfactory receptors involved in detecting odorants and adapting to the chemical complexity of habitats [55,56], as well as MHC genes, central to the immune response to diseases [57,58]. These multicopy gene families found within divergent hotspots may represent adaptation mechanisms that maintain flexibility in response to dynamic or disruptive selective pressures, potentially aiding immune variability, environmental

sensing, and essential survival responses across the diverse habitats these turtles inhabit. Sea turtles are known to inhabit a vast proportion of the globe's seas, found in both deep and shallow waters [47,48], migrating long distances across highly variable temperatures, currents and salinity [37], as well as possessing an immune system highly influenced by these changing environments [49,50]. As enhanced MHC variation is associated with lower disease susceptibility [59], the MHC gene copy numbers and heterozygosity in sea turtles have been previously proposed to vary among species based on their habitats, with those exposed to higher pathogen loads and diversity in neritic environments exhibiting greater MHC gene copy numbers than species inhabiting pelagic habitats, an area which would require manual validation in future studies [14]. Thus, these genome hotspots of increased diversity and divergence may hold the key to understanding chemosensory evolution, disease resistance, and phenotypic diversity in sea turtles. Further exploration of these regions could shed light on adaptive forces that have influenced the evolutionary trajectory of sea turtle species.

From a conservation perspective, genomic resources offer powerful tools to help understand sea turtle population viability and resilience to anthropogenic threats. Genomic diversity, inbreeding levels, effective population sizes, and demographic histories are critical metrics for assessing extinction risk and adaptive potential [60]. Our results indicate that *N. depressus* has maintained a long-term low population size and genetic diversity, similar to the demographic trajectory observed for *D. coriacea* [14], rather than a sharp loss due to recent declines, highlighting the need to distinguish historical demographic patterns from contemporary inbreeding. While reduced diversity may have been sustainable in the past, potentially leading to some degree of purging of

deleterious alleles, it could still limit adaptive capacity in the face of rapid environmental change [61]. In contrast, *Lepidochelys* species exhibit comparatively high genetic diversity despite their historically small population sizes, with *L. kempii* retaining higher genetic variation even with its restricted distribution in the Gulf of Mexico (Table S5). This contrasts with the other range-restricted sea turtle species, *N. depressus*, suggesting that endemism alone does not consistently predict genetic diversity in sea turtles. We acknowledge that our results are based on a single individual, and the individual's origin should be considered, as previous studies have highlighted different demographic histories and genetic diversity between ocean basins [62]. However we have previously shown demographic histories of *C. mydas* and *D. coriacea* to be consistent even from individuals in different populations [14]. In the case of range-restricted species such as *N. depressus* and *L. kempii*, we anticipate that the demographic histories likely reflect range-wide patterns.

The demographic histories of sea turtle species reveal broadly similar trajectories of expanding and contracting effective population size over the past ten million years, though with the magnitude of N_e varying between the species. These unique patterns likely reflect the intersections of species-specific life histories and changing environments such as fluctuations in ocean temperature, sea level, and connectivity [63,64]. Species inhabiting shallow coastal habitats, such as *N. depressus*, were likely particularly affected by the dynamic coastal environment [65]. Specifically, the low and stable population size of *N. depressus* may reflect its restricted neritic distribution and tendency to disperse over smaller distances compared to other sea turtle species, potentially limiting foraging opportunities, preventing the species from achieving the global distribution exhibited at

some other turtles [66]. On the other hand, the historically low population size of *D. coriacea* may be attributed to its specialized open-ocean cold-water lifestyle and high trophic position, primarily consuming gelatinous zooplankton, along with behavioral constraints tied to its large size and the challenges of terrestrial nesting [29]. The early pleistocene glaciation appears to have impacted dermochelyids more severely than the chelonids, resulting in the extinction of all but one of the dermochelyid species [64], which subsequently entered the Pleistocene expansion as a severely bottlenecked remnant population [67]. Conversely, *E. imbricata*, *C. caretta*, and *C. mydas*, fared better during the glacial contraction [68] and experienced more pronounced population expansions during the Pleistocene. The ability of *E. imbricata* to exploit diverse habitats and food sources, with a diet centered on coral reef organisms, likely favored its population expansion by reducing interspecies competition [69]. Similarly, *C. caretta* and *C. mydas* may have benefited from their broad dietary flexibility and ability to thrive in diverse temperate and tropical environments [29].

We observed a strong synchrony in population expansions, with population peaks between 300,000 and the Eemian period (130,000-115,000 years ago), although the magnitude of demographic changes varied among lineages. Reid et al. (2019) [62] also reported a synchronized demographic response after the Last Glacial Maximum across most sea turtle lineages. These population expansions most likely helped maintain genetic diversity in these species. Expanding these comparisons to include individuals from additional populations might further corroborate the links between demographic history and ecological factors such as habitat specificity, feeding habits, thermal preference, developmental and adult foraging stages (oceanic vs. neritic), and

environmental conditions. This will be particularly valuable for estimating recent changes in population size, which rely on population-level genomic resources [70], and for understanding their connection to human-mediated environmental disturbances.

5. Potential Implications

High-quality reference genomes are important building blocks for creating genomic toolkits for species conservation and management. One exciting consequence of discovering the levels of genome-wide synteny exhibited between sea turtles is that genetic markers identified for determining features such as sex and adaptive traits in one species may also be directly applicable to other species without the need for new rounds of research and development. Having complete, annotated, chromosome-level genomes for all sea turtles means that such markers or genetic regions can be quickly verified between the species and turned into practical conservation toolkits. While they may not be required for individual studies with a scope of a single or few populations, they are critical for anchoring markers and comparing across studies and species. For example, the advancement from mitochondrial to whole-genome markers help alleviate conflicting signals that can arise from nuclear integrations of mitochondrial sequences (NuMTs), recently misinterpreted as evidence of a new species of *D. coriacea* [71,72], giving better resolution to future genomic studies with potential conservation implications. In this case the identified NuMT contained a portion of the mtDNA Control Region, commonly used for population structure analysis in all the sea turtle species, however we did not find such NuMTs in the other genomes reported here.

We believe these reference genomes will also be valuable for measuring and predicting the impact of climate change on sea turtles. For ectotherms like reptiles, climate impacts may be particularly pronounced due to their sensitivity to thermal fluctuations [73]. For sea turtles, these effects have potential to be even greater due to their temperature-dependent sex determination, where changes to nest temperature can disrupt sex ratios and reproductive success [54,74]. As these effects may be best evidenced via the epigenome, having access to complete, annotated reference genomes increases the predictive power of markers based on measuring levels of DNA or chromatin modifications.

Our findings highlight how different sea turtle species have responded to ancient climate changes, reflecting a range of adaptive strategies and unique biogeographic scenarios. Understanding how species have historically responded to changes in climate offers insights into their potential reactions to current and future anthropogenic disturbances, helping to inform conservation strategies and predict the long-term impacts of climate shifts on sea turtle populations.

6. Methods

6.1. Sampling

Whole blood samples were collected from juvenile *C. caretta, L. kempii, L. olivacea* and *E. imbricata,* and immediately flash-frozen at -80ºC. A blood sample from a female *N. depressus* was collected as described in Young et al. [75] and subsequently stored in ice for 24 hours before being frozen at -80ºC. Additionally, organ tissue samples were collected opportunistically from *L. kempii* (brain, kidney, and ovary) and *C. caretta*

(thymus, ovary, brain, liver, heart, spleen, testes, kidney, and lung) and flash frozen at -80ºC for long and short read transcriptomic sequencing for genome annotation. We shipped the samples on dry ice or in liquid nitrogen dry shipper, ensuring that they remained consistently frozen throughout transit.

The sampled *C. caretta* and *L. olivacea* individuals were originally stranded on the coast of Oregon, USA in 2021 and 2022 at 44.9426 N, 124.024 W and 44.5455 N, 124.0751 W, respectively and are part of the loggerhead North Pacific Regional Management Unit (RMU), including nesting beaches in Japan and foraging and migration through the North Pacific, and the olive ridley East Pacific RMU, including nesting beaches in Mexico and North America and foraging and migration throughout the Pacific, respectively. The sampled *E. imbricata* individual was stranded in Hawaii, USA (20.0334 N, 155.8264 W) and belongs to the relatively small population part of the hawksbill North Central Pacific RMU [76]. The sampled *N. depressus* individual comes from a summer nesting population close to the centre of the range and from within the largest and most genetically diverse Western and Northern Australian stocks (https://www.wamsi.org.au/kmrp/kimberley-marine-research-node-projects). The *L. kempii* individual was sampled at the New England Aquarium as a rehabilitated cold-stun animal from Cape Cod Bay, MA, USA, belonging to the Northwest Atlantic RMU, which constitutes the single population of this species.

6.2. Sample Processing and Sequencing

We extracted and purified DNA using a Bionano SP DNA kit (PN 80042) for *C. caretta*, *E. imbricata*, and *L. kempii*. We used a MagAttract HMW DNA Kit (Qiagen 67563) for *N.*

depressus and *L. kempii*. We measured DNA quantity using triplicate measures and Qubit 3 fluorometer (Invitrogen Qubit dsDNA Broad Range Assay cat no. Q32850) and measured DNA size with an Agilent Femto Pulse. We fragmented the DNA to 15 – 20 kb length prior to library preparation using a Megaruptor 3 (Diagenode, Denville, NJ, USA) and standard hydropores (Cat. No. E07010003).

We prepared the PacBio HiFi libraries using a SMRTbell prep kit 3.0 (Pacific Biosciences PN 102-182-700) and PacBio barcoded primers. We size-selected the libraries to remove DNA under 10kb using a Pippin HT instrument (Sage Science, Beverly, MA, USA). We then quantified the size-selected HiFi libraries with a Qubit 3 Fluorometer (Qubit dsDNA HS Assay Kit), and assessed the average size with an Agilent Femto Pulse.

For *C. caretta*, *E. imbricata*, and *L. kempii* we sequenced HiFi libraries with a PacBio Sequel IIe instrument on 8M SMRT cells (101-389-001) using Binding kit 3.2 (102-333-300) and Sequel II sequencing kit 2.0 (101-820-200), and 40-hour movie time with 2-hour pre-extension. For *N. depressus* and *L. olivacea* we sequenced HiFi libraries with a PacBio Revio instrument using a Revio polymerase kit (102-817-600), Revio sequencing plate (102-587-400), and 24-hour movie with 1.6-hour pre-extension.

For *C. caretta*, *E. imbricata*, *L. kempii* and *L. olivacea*, we prepared Omni-C libraries using the Dovetail Omni-C Kit (Dovetail Genomics, CA) according to the manufacturer's protocol. We then sequenced the Omni-C libraries with the Illumina NovaSeq 6000 platform with 2x150 bp read length. For *N. depressus* we prepared the Hi-C library using the Arima-HiC 2.0 kit (Arima Genomics, Carlsbad, CA, USA) following the manufacturer's

protocol. We then sequenced the Hi-C libraries with the Illumina NovaSeq 6000 platform with 2x150 bp read length.

For Bionano optical mapping, we labelled 750 ng DNA using direct labeling enzyme (DLE1) and the Bionano Prep Direct Label and Stain (DLS) protocol (document number 30206) and then imaged the DNA on the Bionano Saphyr instrument.

To prepare RNA for sequencing, we extracted and purified total RNA using a QIAGEN RNAeasy kit (cat. 74104). We determined the RNA quantity using a Qubit 3 fluorometer (Invitrogen Qubit RNA High Sensitivity (HS) Kit (cat. no. Q32852)) and measured the RNA integrity (RIN) score using an Agilent Fragment Analyzer. We prepared the RNA-Seq libraries using the Illumina Stranded mRNA Prep kit and sequenced the libraries with the Illumina NovaSeq 6000 platform with 2x100bp read length. We generated Iso-Seq cDNA libraries using the NEBNext Single Cell/Low Input cDNA Synthesis & Amplification Module in combination with PacBio's SMRTbell Prep Kit 3.0. We then sequenced the Iso-Seq libraries on a PacBio Sequel IIe machine using a Sequel II 8M SMRTcell.

6.3. Genome Assembly

We performed the assemblies of each genome following the best-practices established by the Vertebrate Genomes Project [5,77]. In particular, we trimmed the raw sequencing reads for adapters using cutadapt v4.9 to remove any remaining PacBio adapter sequences from the PacBio HiFi reads and Illumina adapters from the Hi-C reads. We assembled initial contig sets for each species using hifiasm [78], v0.19.4-9, l2-l3, Hi-C phasing mode, using both PacBio HiFi and Illumina Hi-C reads as input to generate two haplotype-phased sets of contigs. We then removed retained haplotigs from each

assembly with purge-dups [79] v1.2.6, -e. To scaffold the assembled contigs into chromosomes, we used the hybrid-scaffold tool from the Bionano Solve suite (v3.7.0, VGP mode) to scaffold with optical maps and then mapped the Hi-C reads to the set of initial scaffolds using bwa-mem [80] v2.2.1, -5SP -T0 and scaffolded into pseudo-chromosomal units using yahs [81] v1.2a.1. Finally, we performed rounds of manual curation following the Sanger rapid-curation pipeline [82], joining any missed-scaffolds and removing any false joins in the assembly. We screened for any retained adapter or vector sequences using ncbi's FCS-adapter and for foreign contaminant sequences using ncbi's FCS-GX [83] v0.5.4.

6.4. Genome Annotation

To generate a set of protein-coding annotations for each genome, we incorporated evidence from transcript data, protein sequences, *ab-initio* machine-learning approaches and homology to genomes of related species. To create *ab-initio* predictions, we ran Helixer [84] vv0.3.3_cuda_11.8.0 using argument *–lineage vertebrate*. To generate protein-based gene model predictions, we mapped protein sequences from existing *C. mydas* and *D. coriacea* assemblies (GCF_015237465.2 and GCF_009764565.3, respectively) using miniprot [85] v0.13-r248. To create transcript-based gene model predictions, we mapped paired-end RNA-seq data to each genome using hisat2 [86] v2.2.1 using argument *–dta* and filtered the alignments using samtools [87] v1.19.2 with argument *-F 3840*. We then generated a *de-novo* transcript assembly using stringtie [88] v2.2.1 and predicted coding sequences using TransDecoder [89] v5.7.1 and included only those gene models with a TransDecoder score greater than 20. Similarly, we mapped PacBio Iso-seq data to the genome using minimap2 [90] v2.28-r1209 with argument *-x*

splice:hq and filtered the alignments using samtools with argument *-F 3840* and built gene models using stringtie with argument *-L* and predicted CDS using TransDecoder as above. To generate homology-based predictions, we created lastz-alignment chains from *C. mydas* and *Malaclemys terrapin pileata* genomes (GCF_015237465.2 and GCF_027887155.1) using the *make_lastz_chains* (v2.0.8) tools from TOGA [91] and we generated the set of homology gene predictions using TOGA (v1.1.6).

To generate a set of best gene models, we used EvidenceModeler [92] v2.1.0 to combine all of the above evidences using the weights defined in Table S8.

To create functional annotations, we mapped the amino acid sequences from each gene model against the swissprot database [93], release 2023_03 using the diamond [94] v2.1.8 blastp search and we identified Pfam, PROSITE and SUPERFAMILY homology using Interproscan [95,96] v5.59-91.0. Finally, we filtered gene models which had no identified swissprot or Pfam homology and were over 50% masked, or missing start and/or stop codons.

6.5. Genome synteny

To determine the number and sizes of syntenic regions within turtle and tortoise genomes, we made use of the annotated protein sequences to find orthologous genes within the genomes and uncover regions of local syntenic inheritance. By identifying synteny based on orthologous protein sequences, we relied on the unique elements of the genome, ignoring repetitive or other non-coding areas of the genome. We used Oxford Dot Plot [97] v0.3.3 to identify orthologous genes and plot synteny via ribbon plots, particularly the https://github.com/conchoecia/odp/blob/main/scripts/odp_nway_rbh pipeline. We

extracted protein sequences from the annotated chromosomes of each Testudine assembly using the AGAT [98] v1.0.0 command *agat_sp_extract_sequences.pl* and mapped the sequences against each other using the diamond [94] v2.1.9 blastp command with e-value cutoff of 1e-5. Syntenic protein alignments were only included in the next step if the same hit was found to be the best for each pairwise comparison (best reciprocal hits). To determine syntenic blocks, permutation tests were performed, with 10,000 bootstraps and only those syntenic blocks with FDR less than 0.05 included and plotted as distinct colours in the ribbon diagrams. We performed this analysis once using the sea-turtle genomes as input and once with one species per genus for all currently available chromosome-scale reference genomes with annotations on GenBank alongside those from this study (GCF_016161935.1, GCF_007399415.2, GCF_028017835.1, GCF_013100865.1, GCF_027887155.1, GCF_009764565.3 and GCF_015237465.2).

6.6. Phylogenetic analysis

To reconstruct the phylogenetic history of the Testudine clade, we built a tree based on the protein sequences of all reference genomes submitted to GenBank with a protein-coding annotation. For each genome, we reduced the gff files to contain only the longest isoform per gene using the AGAT [98], v1.0.0 command *agat_sp_keep_longest_isoform.pl* and then extracted the protein sequences for each gene using the command *agat_sp_extract_sequences.pl*. To find the single-copy orthologs, we used OrthoFinder [99] v2.5.5.2 using the amino-acid files as input. We then aligned the single-copy orthologs using MAFFT [100] v7.475, trimmed the resulting multi-alignment files using trimAL [101] v1.4.1 with argument -automated1, concatenated the trimmed alignments into supermatrix containing all aligned sequences and constructed a

phylogenetic tree using IQtree [102] v2.2.5 with 1,000 bootstraps (-B 1000). To further estimate the branching points in the tree, we took upper- and lower-bound divergence time estimates from timetree.org for all internal nodes and used these as calibration times for MCMCtree [103] paml v4.10.7 using the JC69 model. A full list of commands can be found in the script “create_tree.sh”.

6.7. Genome-wide diversity and divergence

Aiming to explore the genome-wide patterns of genetic diversity in the sea turtle clade, we performed the SNP calling for the seven sea turtle species using the jATG pipeline (https://github.com/diegomics/jATG/tree/devel). First, we mapped PacBio HiFi reads for the five genomes generated in this work against its own reference genome using minimap2 [90], v2.26, and mapped Illumina 10x reads for *D. coriacea* and *C. mydas* using bwa-mem2 [104] v2.2.1. Following the mapping, we removed PCR duplicates from BAM files using MarkDuplicates from GATK [105] v4.6. We performed variant calling using GATK v4.6 HaplotypeCaller and GenotypeGVCF. We then filtered the resulting GVCF using BCFtools [106] following GATK’s recommended parameter thresholds [105], removing low mapping quality positions (MQ>30), SNPs with depth lower than 8 and greater than 2x the average coverage, and keeping only biallelic positions. We also filtered small scaffolds, keeping only the 28 chromosomes for the subsequent analysis. Additionally, we excluded SNPs located in masked regions from subsequent analyses, identified by masking the genome with Dfam TE Tools v1.85 using RepeatModeler [107] and RepeatMasker [108]. We converted all filtered genotypes to missing data, producing a base-pair resolution gVCF file.

This filtered gVCF was used for genome-wide heterozygosity assessment and runs of homozygosity (ROH) analysis using Darwindow [109]. This tool enables the visualisation of heterozygosity and ROH along the scaffolds, providing a clear visual assessment of the accuracy of the ROH calls. We calculated heterozygosity based on a sliding-window approach with non-overlapping windows of 50 kb, without applying a filter for missing data. We identified ROHs using a heterozygosity threshold calculated from the average genome-wide heterozygosity of each species. A window was considered to have low heterozygosity if its value fell below one-fifth of the mean heterozygosity. The minimum length of a ROH was set to 500 kb, composed of at least 10 adjacent windows of 50 kb. The maximum proportion of missing data per window was 0.7. The inbreeding level was calculated as the proportion of the genome marked as ROH (FROH).

We calculated gene density using a custom Python script (GeneDensityCalculation.py) that counts the number of genes per Mb across the genome. We estimated pairwise genetic distances using a window-based approach, leveraging genome alignments generated with Progressive Cactus [110] v2.9. The repeat-masked genomes were aligned to ensure that unique regions of the genome were correctly aligned, while repetitive regions were excluded from the mapped regions. We used the halSnps pipeline to identify interspecific single variants, and the halAlignmentDepth pipeline to define 10 kb windows of aligned regions across the genome. We defined genetic distance in each window as the ratio of interspecific single variants per 10 kb. We then identified hotspots of genetic divergence, diversity and gene density by screening these metrics along the chromosomes and targeting windows where heterozygosity was higher than four times the chromosome mean and genetic distance exceeded twice the chromosome mean.

6.8. Demographic analysis

We inferred the demographic histories of the five sea turtle species whose genomes were assembled in this study using the Pairwise Sequentially Markovian Coalescent (PSMC) model [46]. We first extracted the consensus sequence from the filtered gVCF files generated above with BCFtools, then converted the resulting consensus fasta file into the PSMC input format using fq2psmcfa. We ran PSMC using default parameters: -N25 -t15 -r5 -p "4+25*2+4+6", scaling the output assuming a mutation rate (μ) of 1.2×10^{-8} per site per generation and a generation time of 30 years. Given the uncertainty in generation time estimates across species and the variability reported in the literature for each, we selected a generation time of 30 years as an approximate midpoint of reported values. This choice provides a reasonable and biologically plausible basis for our analyses, as previously tested by Bentley et al. [14]. We conducted an additional PSMC analysis using data exclusively from the 11 macrochromosomes, excluding their identified high-diversity regions (Table S9), which may be subject to balancing or diversifying selection, potentially biasing the demographic inferences [111].

Demographic history was further reconstructed using the Multiple Sequentially Markovian Coalescent model (MSMC2; [112]). Input files were prepared with utilities from the MSMC toolkit (https://github.com/stschiff/msmc-tools). Initially, filtered gVCF files, restricted to the 28 autosomes, were processed using the VCFAllSiteParser.py script to create individual masking files. To account for genome regions with reliable read mapping, a mappability mask was generated using SNPable (https://lh3lh3.users.sourceforge.net/snpable.shtml), identifying uniquely mappable loci in the reference genome. The formatted input for MSMC2 was then created using the

generate_multihetsep.py script. For robustness, we produced 50 bootstrap replicates with multihetsep_bootstrap.py (parameters: -n 50 -s 20000000 --chunks_per_chromosome 10). MSMC2 analyses were executed using the default time segment pattern (1*2+25*1+1*2+1*3), incorporating both the individual masks and the mappability mask for each chromosome. Effective population size estimates were scaled based on the previously specified per-generation mutation rate and generation time.

6.9 Hotspot gene family annotation

To further refine the annotation of gene families in the identified hotspot regions, we widened the search to include more functional databases available in InterProScan. Using the *D. coriacea* RefSeq annotation, we extracted the amino-acid sequence of the longest isoform for each gene using *agat_sp_keep_longest_isoform.pl* and the protein sequence using *agat_sp_extract_sequences.pl* as above and used these protein sequences as input to InterProScan (InterPro v102.0), using the Pfam [113], PRINTS [114], SUPERFAMILY [115], PANTHER [116], Gene3D [117], FunFam [118] and SMART [119] databases. To annotate genes belonging to multi-copy gene families, we classified genes as “MHC”, “Immunology-related”, “G-Protein Coupled Receptor” (GPCR), “Olfactory Receptor” or “Zinc-Finger” following terminology described in Table S10. Genes that did not fall into any of these multi-copy gene families were classified as "Other". We then tested the enrichment of these multi-copy gene families against all other protein-coding genes annotated via Fisher’s exact test, followed by Benjamini-Hochberg correction of p-values to account for multiple testing. Full R script is available in the file “enrichment_test.R”.

7. Data Availability

Sequencing data, genome assemblies and annotations are available via the European Nucleotide Archive (ENA) or National Centre for Biotechnology Information (NCBI) under the following umbrella BioProjects: *Caretta caretta* PRJNA1212178, *Eretmochelys imbricata* PRJNA1212183, *Lepidochelys kempii* PRJNA1212180, *Lepidochelys olivacea* PRJNA1212179 and *Natator depressus* PRJNA1212185. Workflows used to generate genome assemblies are published on WorkflowHub under the following collection: https://workflowhub.eu/collections/10 [120–126] and including the BioNano scaffolding workflow from the Vertebrate Genomes Project https://workflowhub.eu/workflows/643. To perform read mapping and SNP calling as well as calculating runs of homozygosity, we used the jATG pipeline available on GitLab: https://github.com/diegomics/jATG/tree/devel. Custom scripts written are published in gitlab: https://git.imp.fu-berlin.de/begendiv/sea_turtlegenomes. Commands run to generate protein-coding annotations are available in the script "annotation_commands.sh", custom script to calculate gene density is in the python file "geneDensityCalculation.py".

References

1. Hogg CJ. Translating genomic advances into biodiversity conservation. *Nat Rev Genet*. Springer Science and Business Media LLC; 25:362–732024;

2. Lewin HA, Robinson GE, Kress WJ, Baker WJ, Coddington J, Crandall KA, et al.. Earth BioGenome Project: Sequencing life for the future of life. *Proc Natl Acad Sci U S A*. 115:4325–332018;

3. Mc Cartney AM, Formenti G, Mouton A, De Panis D, Marins LS, Leitão HG, et al.. The European Reference Genome Atlas: piloting a decentralised approach to equitable biodiversity genomics. *NPJ Biodivers*. Springer Science and Business Media LLC; 3:282024;

4. Darwin Tree of Life Project Consortium. Sequence locally, think globally: The Darwin Tree of

Life Project. *Proc Natl Acad Sci U S A*. Proceedings of the National Academy of Sciences; 119:e21156421182022;

5. Rhie A, McCarthy SA, Fedrigo O, Damas J, Formenti G, Koren S, et al.. Towards complete and error-free genome assemblies of all vertebrate species. *Nature*. 592:737–462021;

6. Budd AM, Mayne B, Berry O, Jarman S. Fish species lifespan prediction from promoter cytosine-phosphate-guanine density. *Mol Ecol Resour*. Wiley; 2023; doi: 10.1111/1755-0998.13774.

7. Mayne B, Mustin W, Baboolal V, Casella F, Ballorain K, Barret M, et al.. Age prediction of green turtles with an epigenetic clock. *Mol Ecol Resour*. Wiley; 22:2275–842022;

8. Holleley CE, Whiteley SL, Devloo-Delva F, Bachler A, Llinas J, Georges A. 8 Molecular sex identification for applications in conservation, industry and veterinary medicine. *Applied Environmental Genomics*. 742023;

9. Bravington MV, Grewe PM, Davies CR. Absolute abundance of southern bluefin tuna estimated by close-kin mark-recapture. *Nat Commun*. 7:131622016;

10. Takahashi M, Saccò M, Kestel JH, Nester G, Campbell MA, van der Heyde M, et al.. Aquatic environmental DNA: A review of the macro-organismal biomonitoring revolution. *Sci Total Environ*. Elsevier BV; 873:1623222023;

11. Breed MF, Harrison PA, Blyth C, Byrne M, Gaget V, Gellie NJC, et al.. The potential of genomics for restoring ecosystems and biodiversity. *Nat Rev Genet*. Springer Science and Business Media LLC; 20:615–282019;

12. Bernatchez L, Ferchaud A-L, Berger CS, Venney CJ, Xuereb A. Genomics for monitoring and understanding species responses to global climate change. *Nat Rev Genet*. Springer Science and Business Media LLC; 25:165–832024;

13. Theissinger K, Fernandes C, Formenti G, Bista I, Berg PR, Bleidorn C, et al.. How genomics can help biodiversity conservation. *Trends Genet*. Elsevier BV; 39:545–592023;

14. Bentley BP, Carrasco-Valenzuela T, Ramos EKS, Pawar H, Souza Arantes L, Alexander A, et al.. Divergent sensory and immune gene evolution in sea turtles with contrasting demographic and life histories. *Proc Natl Acad Sci U S A*. 120:e22010761202023;

15. Shaffer HB, McCartney-Melstad E, Near TJ, Mount GG, Spinks PQ. Phylogenomic analyses of 539 highly informative loci dates a fully resolved time tree for the major clades of living turtles (Testudines). *Mol Phylogenet Evol*. 115:7–152017;

16. Wallace BP, DiMatteo AD, Bolten AB, Chaloupka MY, Hutchinson BJ, Abreu-Grobois FA, et al.. Global conservation priorities for marine turtles. *PLoS One*. Public Library of Science (PLoS); 6:e245102011;

17. Fossette S, Witt MJ, Miller P, Nalovic MA, Albareda D, Almeida AP, et al.. Pan-atlantic analysis of the overlap of a highly migratory species, the leatherback turtle, with pelagic longline fisheries. *Proc Biol Sci*. The Royal Society; 281:201330652014;

18. Simantiris N. The impact of climate change on sea turtles: Current knowledge, scientometrics, and mitigation strategies. *Sci Total Environ*. Elsevier BV; 923:1713542024;

19. IUCN: Chelonia mydas: Seminoff, J.a. (southwest fisheries science center, U.s.). IUCN Red List of Threatened Species. IUCN; http://dx.doi.org/10.2305/iucn.uk.2023-1.rlts.t4615a247654386.en (2004). Accessed 2024 Dec 13.

20. IUCN: Eretmochelys imbricata: Mortimer, J.A & Donnelly, M. (IUCN SSC Marine Turtle Specialist Group). IUCN Red List of Threatened Species. IUCN; http://dx.doi.org/10.2305/iucn.uk.2008.rlts.t8005a12881238.en (2008). Accessed 2024 Dec 13.

21. IUCN: Lepidochelys kempii: Wibbels, T. & Bevan, E. IUCN Red List of Threatened Species. IUCN; http://dx.doi.org/10.2305/iucn.uk.2019-2.rlts.t11533a155057916.en (2019). Accessed 2024 Dec 13.

22. IUCN: Lepidochelys olivacea: Abreu-Grobois, A & Plotkin, P. (IUCN SSC Marine Turtle Specialist Group). IUCN Red List of Threatened Species. IUCN; http://dx.doi.org/10.2305/iucn.uk.2008.rlts.t11534a3292503.en (2008). Accessed 2024 Dec 13.

23. IUCN: Caretta caretta: Casale, P. & Tucker, A.D. IUCN Red List of Threatened Species. IUCN; http://dx.doi.org/10.2305/iucn.uk.2017-2.rlts.t3897a119333622.en (2015). Accessed 2024 Dec 13.

24. IUCN: Dermochelys coriacea: Wallace, B.P., Tiwari, M. & Girondot, M. IUCN Red List of Threatened Species. IUCN; http://dx.doi.org/10.2305/iucn.uk.2013-2.rlts.t6494a43526147.en (2013). Accessed 2024 Dec 13.

25. Standards RL, Petitions Subcommittee: IUCN Red List of Threatened Species: Natator depressus. https://www.iucnredlist.org/species/14363/210612474 (1996). Accessed 2024 Dec 13.

26. : Flatback turtle - Natator depressus. https://www.dcceew.gov.au/environment/biodiversity/threatened/publications/flatback-turtle-natator-depressus-2008#dcceew-main Accessed 2025 Jan 20.

27. Mazaris AD, Schofield G, Gkazinou C, Almpanidou V, Hays GC. Global sea turtle conservation successes. *Sci Adv*. American Association for the Advancement of Science (AAAS); 3:e16007302017;

28. Laúd OPO Network. Enhanced, coordinated conservation efforts required to avoid extinction of critically endangered Eastern Pacific leatherback turtles. *Sci Rep*. Springer Science and Business Media LLC; 10:47722020;

29. Hendrickson JR. The ecological strategies of sea turtles. *Am Zool*. Oxford University Press (OUP); 20:597–6081980;

30. Pritchard PCH. Evolution, Phylogeny, and Current Status. In: Musick PL, Lutz J, editors. *The Biology of Sea Turtles*. CRC Press; p. 28.

31. Bjorndal KA. Foraging ecology and nutrition of sea turtles. *The biology of sea turtles, volume I*. CRC Press; p. 199.

32. Jebb D, Huang Z, Pippel M, Hughes GM, Lavrichenko K, Devanna P, et al.. Six reference-quality genomes reveal evolution of bat adaptations. *Nature*. Springer Science and Business Media LLC; 583:578–842020;

33. Willoughby JR, Harder AM, Tennessen JA, Scribner KT, Christie MR. Rapid genetic adaptation to a novel environment despite a genome-wide reduction in genetic diversity. *Mol Ecol*. 27:4041–512018;

34. Feng S, Fang Q, Barnett R, Li C, Han S, Kuhlwilm M, et al.. The Genomic Footprints of the Fall and Recovery of the Crested Ibis. *Curr Biol*. 29:340–9.e72019;

35. Chang G, Jones S, Leelakumari S, Ashkani J, Culibrk L, O'Neill K, et al.. The genome sequence of the Loggerhead sea turtle, Caretta caretta Linnaeus 1758. *F1000Res*. 12:3362023;

36. Guo Y, Tang J, Zhuo Z, Huang J, Fu Z, Song J, et al.. The first high-quality chromosome-level genome of Eretmochelys imbricata using HiFi and Hi-C data. *Sci Data*. Springer Science and Business Media LLC; 10:6042023;

37. Yang L, Chen Y, Wang S, Zhang C, Huang X, Du X, et al.. Genomic insights into marine environment adaptation and conservation of the threatened olive ridley turtle (Lepidochelys olivacea). *iScience*. Elsevier BV; 28:1117762025;

38. Yen EC, Gilbert JD, Balard A, Taxonera A, Fairweather K, Ford HL, et al.. Chromosome-level genome assembly and methylome profile enables insights for the conservation of endangered loggerhead sea turtles. bioRxiv.

39. Duchene S, Frey A, Alfaro-Núñez A, Dutton PH, Thomas P Gilbert M, Morin PA. Marine turtle mitogenome phylogenetics and evolution. *Mol Phylogenet Evol*. Elsevier BV; 65:241–502012;

40. Naro-Maciel E, Le M, FitzSimmons NN, Amato G. Evolutionary relationships of marine turtles: A molecular phylogeny based on nuclear and mitochondrial genes. *Mol Phylogenet Evol*. Elsevier BV; 49:659–622008;

41. Thomson RC, Spinks PQ, Shaffer HB. A global phylogeny of turtles reveals a burst of climate-associated diversification on continental margins. *Proc Natl Acad Sci U S A*. Proceedings of the National Academy of Sciences; 118:e20122151182021;

42. Vilaça ST, Hahn AT, Naro-Maciel E, Abreu-Grobois FA, Bowen BW, Castilhos JC, et al.. Global phylogeography of ridley sea turtles (Lepidochelys spp.): evolution, demography, connectivity, and conservation. *Conserv Genet*. Springer Science and Business Media LLC; 23:995–10102022;

43. Vilaça ST, Piccinno R, Rota-Stabelli O, Gabrielli M, Benazzo A, Matschiner M, et al.. Divergence and hybridization in sea turtles: Inferences from genome data show evidence of ancient gene flow between species. *Mol Ecol*. Wiley; 30:6178–922021;

44. Waters PD, Patel HR, Ruiz-Herrera A, Álvarez-González L, Lister NC, Simakov O, et al.. Microchromosomes are building blocks of bird, reptile, and mammal chromosomes. *Proc Natl Acad Sci U S A*. Proceedings of the National Academy of Sciences; 2021; doi: 10.1073/pnas.2112494118.

45. Ceballos FC, Joshi PK, Clark DW, Ramsay M, Wilson JF. Runs of homozygosity: windows into population history and trait architecture. *Nat Rev Genet*. 19:220–342018;

46. Li H, Durbin R. Inference of human population history from individual whole-genome

sequences. *Nature*. Springer Science and Business Media LLC; 475:493–62011;

47. Niimura Y, Nei M. Extensive gains and losses of olfactory receptor genes in mammalian evolution. *PLoS One*. Public Library of Science (PLoS); 2:e7082007;

48. Arantes LS, Vilaça ST, Mazzoni CJ, Santos FR. New genetic insights about hybridization and population structure of hawksbill and loggerhead turtles from Brazil. *J Hered*. Oxford University Press (OUP); 111:444–562020;

49. Bhattacharyya T, Gregorova S, Mihola O, Anger M, Sebestova J, Denny P, et al.. Mechanistic basis of infertility of mouse intersubspecific hybrids. *Proc Natl Acad Sci U S A*. Proceedings of the National Academy of Sciences; 110:E468–772013;

50. Luschi P, Hays GC, Papi F. A review of long-distance movements by marine turtles, and the possible role of ocean currents. *Oikos*. Wiley; 103:293–3022003;

51. Reina RD, Jones TT, Spotila JR. Salt and water regulation by the leatherback sea turtle Dermochelys coriacea. *J Exp Biol*. 205:1853–602002;

52. Lohmann K, Lohmann C, Brothers J, Putman N. Natal homing and imprinting in sea turtles. *The Biology of Sea Turtles, Volume III*. CRC Press; p. 59–78.

53. Lohmann KJ, Putman NF, Lohmann CMF. Geomagnetic imprinting: A unifying hypothesis of long-distance natal homing in salmon and sea turtles. *Proc Natl Acad Sci U S A*. Proceedings of the National Academy of Sciences; 105:19096–1012008;

54. Santidrián Tomillo P, Spotila JR. Temperature-dependent sex determination in sea turtles in the context of climate change: Uncovering the adaptive significance. *Bioessays*. Wiley; 42:e20001462020;

55. Yohe LR, Fabbri M, Hanson M, Bhullar B-AS. Olfactory receptor gene evolution is unusually rapid across Tetrapoda and outpaces chemosensory phenotypic change. *Curr Zool*. Oxford University Press (OUP); 66:505–142020;

56. Niimura Y, Nei M. Evolutionary dynamics of olfactory and other chemosensory receptor genes in vertebrates. *J Hum Genet*. Springer Science and Business Media LLC; 51:505–172006;

57. Sommer S. The importance of immune gene variability (MHC) in evolutionary ecology and conservation. *Front Zool*. Springer Nature; 2:162005;

58. Elbers J. P. Taylor SS. Major histocompatibility complex polymorphism in reptile conservation. *Herpetological Conservation and Biology*. 11:1–122016;

59. Siddle HV, Marzec J, Cheng Y, Jones M, Belov K. MHC gene copy number variation in Tasmanian devils: implications for the spread of a contagious cancer. *Proc Biol Sci*. The Royal Society; 277:2001–62010;

60. van Oosterhout C, Speak SA, Birley T, Bortoluzzi C, Percival-Alwyn L, Urban LH, et al.. Genomic erosion in the assessment of species extinction risk and recovery potential. *bioRxiv*. Cold Spring Harbor Laboratory; 2022; doi: 10.1101/2022.09.13.507768.

61. Kardos M, Armstrong EE, Fitzpatrick SW, Hauser S, Hedrick PW, Miller JM, et al.. The

crucial role of genome-wide genetic variation in conservation. *Proc Natl Acad Sci U S A*. Proceedings of the National Academy of Sciences; 118:e21046421182021;

62. Reid BN, Naro-Maciel E, Hahn AT, FitzSimmons NN, Gehara M. Geography best explains global patterns of genetic diversity and postglacial co-expansion in marine turtles. *Mol Ecol*. Wiley; 28:3358–702019;

63. Pike DA. Climate influences the global distribution of sea turtle nesting. *Glob Ecol Biogeogr*. Wiley; 22:555–662013;

64. Chen I-C, Hill JK, Ohlemüller R, Roy DB, Thomas CD. Rapid range shifts of species associated with high levels of climate warming. *Science*. American Association for the Advancement of Science (AAAS); 333:1024–62011;

65. Williams AN, Ulm S, Sapienza T, Lewis S, Turney CSM. Sea-level change and demography during the last glacial termination and early Holocene across the Australian continent. *Quat Sci Rev*. Elsevier BV; 182:144–542018;

66. Bolten A. Variation in sea turtle life history patterns. *Marine Biology*. CRC Press; p. 243–57.

67. Dutton PH, Bowen BW, Owens DW, Barragan A, Davis SK. Global phylogeography of the leatherback turtle (Dermochelys coriacea). *J Zool (1987)*. Wiley; 248:397–4091999;

68. Jensen MP, FitzSimmons NN, Bourjea J, Hamabata T, Reece J, Dutton PH. The evolutionary history and global phylogeography of the green turtle (*Chelonia mydas*). *J Biogeogr*. Wiley; 46:860–702019;

69. Moorehouse MA, Baldwin JD, Hart KM. Hawksbill and green turtle niche overlap in a marine protected area, US Virgin Islands. *Endanger Species Res*. Inter-Research Science Center; 52:265–832023;

70. Santiago E, Novo I, Pardiñas AF, Saura M, Wang J, Caballero A. Recent Demographic History Inferred by High-Resolution Analysis of Linkage Disequilibrium. *Mol Biol Evol*. 37:3642–532020;

71. Colombo WD, de Freitas Justino J, Barcelos AC, Vilaça ST, Pavanelli L, Vargas SM. Reassessing leatherback turtle lineages and unveiling the first evidence of nuclear mitochondrial DNA in sea turtles. *Sci Rep*. Springer Science and Business Media LLC; 14:313132024;

72. Castillo-Morales CA, Sáenz-Arroyo A, Castellanos-Morales G, Ruíz-Montoya L. Mitochondrial DNA and local ecological knowledge reveal two lineages of leatherback turtle on the beaches of Oaxaca, Mexico. *Sci Rep*. Springer Science and Business Media LLC; 13:88362023;

73. Hayden Bofill SI, Blom MPK. Climate change from an ectotherm perspective: evolutionary consequences and demographic change in amphibian and reptilian populations. *Biodivers Conserv*. Springer Science and Business Media LLC; 33:905–272024;

74. Maurer AS, Seminoff JA, Layman CA, Stapleton SP, Godfrey MH, Reiskind MOB. Population viability of sea turtles in the context of global warming. *Bioscience*. Oxford University Press (OUP); 71:790–8042021;

75. Young EJ, Vaughan-Higgins R, Warren KS, Whiting SD, Rossi G, Stephens NS, et al.. Novel Haemocystidium sp. Intraerythrocytic Parasite in the Flatback (Natator depressus) and Green (Chelonia mydas) Turtle in Western Australia. *Pathogens*. 2024; doi: 10.3390/pathogens13121112.

76. Wallace BP, Posnik ZA, Hurley BJ, DiMatteo AD, Bandimere A, Rodriguez I, et al.. Marine turtle regional management units 2.0: an updated framework for conservation and research of wide-ranging megafauna species. *Endanger Species Res*. Inter-Research Science Center; 52:209–232023;

77. Larivière D, Abueg L, Brajuka N, Gallardo-Alba C, Grüning B, Ko BJ, et al.. Scalable, accessible and reproducible reference genome assembly and evaluation in Galaxy. *Nat Biotechnol*. 42:367–702024;

78. Cheng H, Concepcion GT, Feng X, Zhang H, Li H. Haplotype-resolved de novo assembly using phased assembly graphs with hifiasm. *Nat Methods*. Springer Science and Business Media LLC; 18:170–52021;

79. Guan D, McCarthy SA, Wood J, Howe K, Wang Y, Durbin R. Identifying and removing haplotypic duplication in primary genome assemblies. *Bioinformatics*. Oxford University Press (OUP); 36:2896–82020;

80. Li H. Aligning sequence reads, clone sequences and assembly contigs with BWA-MEM. arXiv [q-bio.GN].

81. Zhou C, McCarthy SA, Durbin R. YaHS: yet another Hi-C scaffolding tool. *Bioinformatics*. Oxford University Press (OUP); 2023; doi: 10.1093/bioinformatics/btac808.

82. Howe K, Chow W, Collins J, Pelan S, Pointon D-L, Sims Y, et al.. Significantly improving the quality of genome assemblies through curation. *Gigascience*. Oxford University Press (OUP); 2021; doi: 10.1093/gigascience/giaa153.

83. Astashyn A, Tvedte ES, Sweeney D, Sapojnikov V, Bouk N, Joukov V, et al.. Rapid and sensitive detection of genome contamination at scale with FCS-GX. *Genome Biol*. Springer Science and Business Media LLC; 25:602024;

84. Stiehler F, Steinborn M, Scholz S, Dey D, Weber APM, Denton AK. Helixer: cross-species gene annotation of large eukaryotic genomes using deep learning. *Bioinformatics*. Oxford University Press (OUP); 36:5291–82021;

85. Li H. Protein-to-genome alignment with miniprot. *Bioinformatics*. Oxford University Press (OUP); 2023; doi: 10.1093/bioinformatics/btad014.

86. Kim D, Paggi JM, Park C, Bennett C, Salzberg SL. Graph-based genome alignment and genotyping with HISAT2 and HISAT-genotype. *Nat Biotechnol*. Springer Science and Business Media LLC; 37:907–152019;

87. Li H, Handsaker B, Wysoker A, Fennell T, Ruan J, Homer N, et al.. The Sequence Alignment/Map format and SAMtools. *Bioinformatics*. Oxford University Press (OUP); 25:2078–92009;

88. Pertea M, Pertea GM, Antonescu CM, Chang T-C, Mendell JT, Salzberg SL. StringTie

enables improved reconstruction of a transcriptome from RNA-seq reads. *Nat Biotechnol*. Springer Science and Business Media LLC; 33:290–52015;

89. Haas BJ, Papanicolaou A, Yassour M, Grabherr M, Blood PD, Bowden J, et al.. De novo transcript sequence reconstruction from RNA-seq using the Trinity platform for reference generation and analysis. *Nat Protoc*. Springer Science and Business Media LLC; 8:1494–5122013;

90. Li H. Minimap2: pairwise alignment for nucleotide sequences. *Bioinformatics*. Oxford University Press (OUP); 34:3094–1002018;

91. Kirilenko BM, Munegowda C, Osipova E, Jebb D, Sharma V, Blumer M, et al.. Integrating gene annotation with orthology inference at scale. *Science*. 380:eabn31072023;

92. Haas BJ, Salzberg SL, Zhu W, Pertea M, Allen JE, Orvis J, et al.. Automated eukaryotic gene structure annotation using EVidenceModeler and the Program to Assemble Spliced Alignments. *Genome Biol*. Springer Nature; 9:R72008;

93. Bairoch A, Apweiler R. The SWISS-PROT protein sequence database and its supplement TrEMBL in 2000. *Nucleic Acids Res*. Oxford University Press (OUP); 28:45–82000;

94. Buchfink B, Reuter K, Drost H-G. Sensitive protein alignments at tree-of-life scale using DIAMOND. *Nat Methods*. Springer Science and Business Media LLC; 18:366–82021;

95. Jones P, Binns D, Chang H-Y, Fraser M, Li W, McAnulla C, et al.. InterProScan 5: genome-scale protein function classification. *Bioinformatics*. 30:1236–402014;

96. Blum M, Chang H-Y, Chuguransky S, Grego T, Kandasaamy S, Mitchell A, et al.. The InterPro protein families and domains database: 20 years on. *Nucleic Acids Res*. Oxford University Press (OUP); 49:D344–542021;

97. Schultz DT, Haddock SHD, Bredeson JV, Green RE, Simakov O, Rokhsar DS. Ancient gene linkages support ctenophores as sister to other animals. *Nature*. Nature Publishing Group; 618:110–72023;

98. Dainat J, Hereñú D, Davis E, Crouch K, LucileSol, Agostinho N, et al.. NBISweden/AGAT: AGAT-v1.0.0. Zenodo;

99. Emms DM, Kelly S. OrthoFinder: phylogenetic orthology inference for comparative genomics. *Genome Biol*. Springer Science and Business Media LLC; 20:2382019;

100. Katoh K, Standley DM. MAFFT multiple sequence alignment software version 7: improvements in performance and usability. *Mol Biol Evol*. Oxford University Press (OUP); 30:772–802013;

101. Capella-Gutiérrez S, Silla-Martínez JM, Gabaldón T. trimAl: a tool for automated alignment trimming in large-scale phylogenetic analyses. *Bioinformatics*. Oxford University Press (OUP); 25:1972–32009;

102. Minh BQ, Schmidt HA, Chernomor O, Schrempf D, Woodhams MD, von Haeseler A, et al.. IQ-TREE 2: New models and efficient methods for phylogenetic inference in the genomic era. *Mol Biol Evol*. Oxford University Press (OUP); 37:1530–42020;

103. Rannala B, Yang Z. Inferring speciation times under an episodic molecular clock. *Syst Biol*. Oxford University Press (OUP); 56:453–662007;

104. Vasimuddin M, Misra S, Li H, Aluru S. Efficient Architecture-Aware Acceleration of BWA-MEM for Multicore Systems. *2019 IEEE International Parallel and Distributed Processing Symposium (IPDPS)*. IEEE; p. 314–24.

105. Van der Auwera GA, O'Connor BD. Genomics in the Cloud: Using Docker, GATK, and WDL in Terra. "O'Reilly Media, Inc.";

106. Danecek P, Bonfield JK, Liddle J, Marshall J, Ohan V, Pollard MO, et al.. Twelve years of SAMtools and BCFtools. *Gigascience*. 2021; doi: 10.1093/gigascience/giab008.

107. Flynn JM, Hubley R, Goubert C, Rosen J, Clark AG, Feschotte C, et al.. RepeatModeler2 for automated genomic discovery of transposable element families. *Proc Natl Acad Sci U S A*. 117:9451–72020;

108. Smit AFA, Hubley R, Green P: RepeatMasker Open-4.0. http://www.repeatmasker.org (2013-2015).

109. de Jong MJ, Niamir A, Wolf M, Kitchener AC, Lecomte N, Seryodkin IV, et al.. Range-wide whole-genome resequencing of the brown bear reveals drivers of intraspecies divergence. *Commun Biol*. 6:1532023;

110. Armstrong J, Hickey G, Diekhans M, Fiddes IT, Novak AM, Deran A, et al.. Progressive Cactus is a multiple-genome aligner for the thousand-genome era. *Nature*. Springer Science and Business Media LLC; 587:246–512020;

111. Boitard S, Arredondo A, Chikhi L, Mazet O. Heterogeneity in effective size across the genome: effects on the inverse instantaneous coalescence rate (IICR) and implications for demographic inference under linked selection. *Genetics*. Oxford University Press (OUP); 2022; doi: 10.1093/genetics/iyac008.

112. Schiffels S, Wang K. MSMC and MSMC2: The Multiple Sequentially Markovian Coalescent. *Methods Mol Biol*. 2090:147–662020;

113. Mistry J, Chuguransky S, Williams L, Qureshi M, Salazar GA, Sonnhammer ELL, et al.. Pfam: The protein families database in 2021. *Nucleic Acids Res*. Oxford University Press (OUP); 49:D412–92021;

114. Attwood TK, Coletta A, Muirhead G, Pavlopoulou A, Philippou PB, Popov I, et al.. The PRINTS database: a fine-grained protein sequence annotation and analysis resource--its status in 2012. *Database (Oxford)*. Oxford University Press (OUP); 2012:bas0192012;

115. Pandurangan AP, Stahlhacke J, Oates ME, Smithers B, Gough J. The SUPERFAMILY 2.0 database: a significant proteome update and a new webserver. *Nucleic Acids Res*. Oxford University Press (OUP); 47:D490–42019;

116. Thomas PD, Ebert D, Muruganujan A, Mushayahama T, Albou L-P, Mi H. PANTHER: Making genome-scale phylogenetics accessible to all. *Protein Sci*. Wiley; 31:8–222022;

117. Lewis TE, Sillitoe I, Dawson N, Lam SD, Clarke T, Lee D, et al.. Gene3D: Extensive prediction of globular domains in proteins. *Nucleic Acids Res*. Oxford University Press (OUP);

46:D1282–D12822018;

118. Scheibenreif L, Littmann M, Orengo C, Rost B. FunFam protein families improve residue level molecular function prediction. *BMC Bioinformatics*. Springer Science and Business Media LLC; 20:4002019;

119. Letunic I, Bork P. 20 years of the SMART protein domain annotation resource. *Nucleic Acids Res*. Oxford University Press (OUP); 46:D493–62018;

120. De Panis D. ERGA HiC Hap1Hap2 Scaffolding+QC YaHS v2309 (WF4).

121. De Panis D. ERGA HiFi Hap1Hap2 Purge+QC v2309 (WF3).

122. De Panis D. ERGA HiFi+HiC Assembly+QC Hifiasm v2309 (WF2).

123. De Panis D. ERGA DataQC Illumina v2309 (WF0).

124. De Panis D. ERGA Profiling HiFi v2309 (WF1).

125. De Panis D. ERGA DataQC HiFi v2309 (WF0).

126. Gustafsson OJR, Wilkinson SR, Bacall F, Soiland-Reyes S, Leo S, Pireddu L, et al.. WorkflowHub: a registry for computational workflows. *Sci Data*. 12:8372025;

127. Bennett L, Melchers B, Proppe B. Curta: A General-purpose High-Performance Computer at ZEDAT, Freie Universität Berlin. Freie Universität Berlin; 2020; doi: 10.17169/REFUBIUM-26754.

Declarations

N. depressus blood was collected under permit (TFA 2019-0174-2), animal ethics committee approval (2019-12B), and shipped under CITES permit (AU94). *L. kempii* blood and tissue samples were collected under USFWS Permit ES69328D. *E. imbricata* blood was collected under USFWS Permit TE-72088A-3. *C. caretta* and *L. olivacea* blood was collected under USFWS Permit TE86356B-2 (to Sea World), and *C. caretta* embryo tissue samples were collected under Florida Fish and Wildlife Conservation Commission Marine Turtle Permit 073 (FWC-MTP-073).

Competing interests

The authors declare no competing interests.

Funding

The production of sequencing data was funded by a Wild Genomes grant from Revive & Restore via a Catalyst Science Fund (WGM_2021-026) to C.J.M. L.M.K was supported by an NSF-IOS grant (#1904439) and the University of Massachusetts Amherst. P.H.D is supported by NOAA Fisheries. Funding was also provided by CSIRO’s Environomics Future Science Platform.

Authors' contributions

Conceptualisation:CJM, LMK, OB, PHD, BPB

Data Curation: DDP, TB, JB

Formal Analysis: DDP, LSA, TB

Funding Acquisition: CJM, LMK, PHD, BPB, OB

Investigation: DDP, LSA, TB

Methodology: DDP, LSA, TB, CJM, LMK, PHD, BPB, OB, JB, CW, NJ, TT, BO’T, PT

Project Administration: CJM, LMK

Software: DDP, LSA, TB

Resources: SDW, EJY, GC, AK, DE, ELC, OB, PHD, CJM, LMK

Supervision: CJM, LMK, PHD, OB, EDJ

Validation: DDP, LSA, TB, CJM, LMK, PHD, BPB, OB

Visualisation: DDP, LSA, TB

Writing - Original Draft: LSA, TB

Writing - Review & Editing: All

Acknowledgements

The authors would like to thank the HPC Service of FUB-IT, Freie Universität Berlin, for computing time [127], Camryn Allen, Shreya Banerjee, Jamie Adkins, Alexandria Mena, Andra Kurtz, Claudia Cedillo, Itzel Sifuentes-Romero, Jeanette Wyneken and The Rescue and Rehabilitation Department and Animal Health Department of New England Aquarium, especially Charlie Innis, for assistance with sample collection, and the Revive & Restore team for support with project planning. The authors also recognise the contribution of the late Tony Tucker to the collection of flatback turtle samples, and for his lifetime of passionate commitment to sea turtle science.

Figure Legends

Figure 1 A) Lengths of the assembled contigs for each species sorted by length (y-axis) and scaled to total length of each genome (x-axis). Genomes from this study are shown as complete lines with names in bold, previously published assemblies are shown as dashed lines. B) 3-dimensional conformational arrangement of one assembled *Caretta caretta* haplotype genome assembly as evaluated by Hi-C. The x- and y-axes show the coordinates of the respective genome and each detected contact in the genome is coloured with increasing intensity from white to red. The red diagonal shows the self-interactions of each position with itself and close vicinity, squares show the high self-interaction of chromosomes. C) Percentage detected single copy orthologs identified in the genome assemblies calculated via BUSCO using the Sauropsida database. Scores are calculated based on detected mappings of ortholog sequences using the Miniprot mapper. D) Gene completeness of sea-turtle protein-coding annotations based on BUSCO genes identified in each annotated protein set.

Figure 2 A) Genome-wide gene-synteny plots across Testudines. Each line represents a best-reciprocal-hit protein match between annotated genes in each consecutive genome. Lines are coloured based on co-localisation across all 11 genomes determined by Fisher's Exact Test. Chromosomes are ordered based on synteny to Dermochelys coriacea. B) Divergence times of species within the suborder Cryptodira based on protein-coding genome annotations. The bars on each node represent the 95% highest posterior density (HPD) intervals for node age estimates. Divergence times and confidence intervals for each numbered node are detailed in Table S4.

Figure 3 A) Heterozygosity, B) gene density (per Mb) and C) pairwise genetic distance per chromosome for the seven sea turtle reference genomes. Chromosomes longer or shorter than 50 Mb are highlighted as macrochromosomes or microchromosomes, respectively.

Figure 4 Genetic diversity and divergence hotspots contain genes associated with immune response, olfactory receptors, zinc fingers, and G-protein-coupled receptors. A) The heatmap illustrates normalised heterozygosity (He) across chromosomes 13, 14, and 24 for seven sea turtle species, displaying He values in non-overlapping 50 kb windows. The normalisation highlights chromosomal hotspots rather than overall diversity. B) Pairwise genetic distances between the six sea turtle species and D. coriacea are shown along the same three chromosomes. Genetic distance was calculated as the ratio of interspecific single variants per 10 kb. Black boxes highlight the chromosome areas of increased genetic distance among sea turtle genomes. C) Multi-copy gene families located in the highlighted regions are displayed and colour-coded by their annotation. Chromosome coordinates are shown by their position in the genome of *D. coriacea*.

Figure 5 A) Inbreeding levels for the seven sea turtle individuals, measured as the proportion of the genome in runs of homozygosity (FROH). FROH are categorised by length (in Mb), where longer runs indicate more recent events associated with a shared common ancestor of the individual's maternal and paternal lineages, while shorter runs suggest older inbreeding events. B) Ancient demographic history for the seven sea turtle species reconstructed with Pairwise Sequential Markovian Coalescent (PSMC) plot. Dashed lines indicate the Last Interglacial (Eemian Period, 130,000 to 115,000 years ago) and mid-Pleistocene transition (1.2-0.5 million years ago). Bootstrap replicates (10 for each lineage) are plotted in lighter lines. Inferred fluctuations in effective population size (Ne) were rescaled assuming 30-year generation time and 1.2×10−8 per generation mutation rate.

Figure 1

Click here to access/download;Figure;Figure_1.pdf

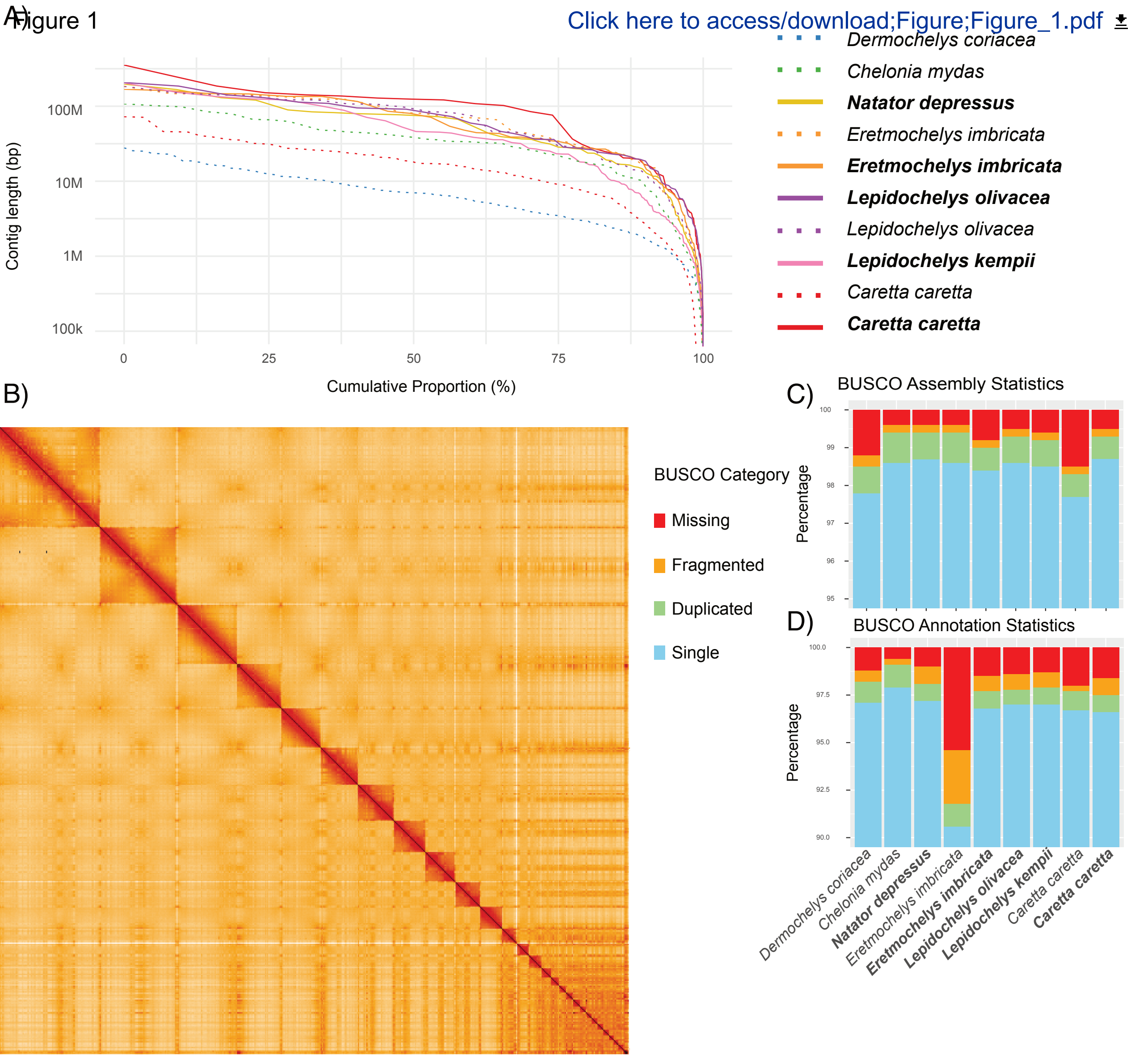

Figure 2

Click here to access/download;Figure;Figure_2.pdf

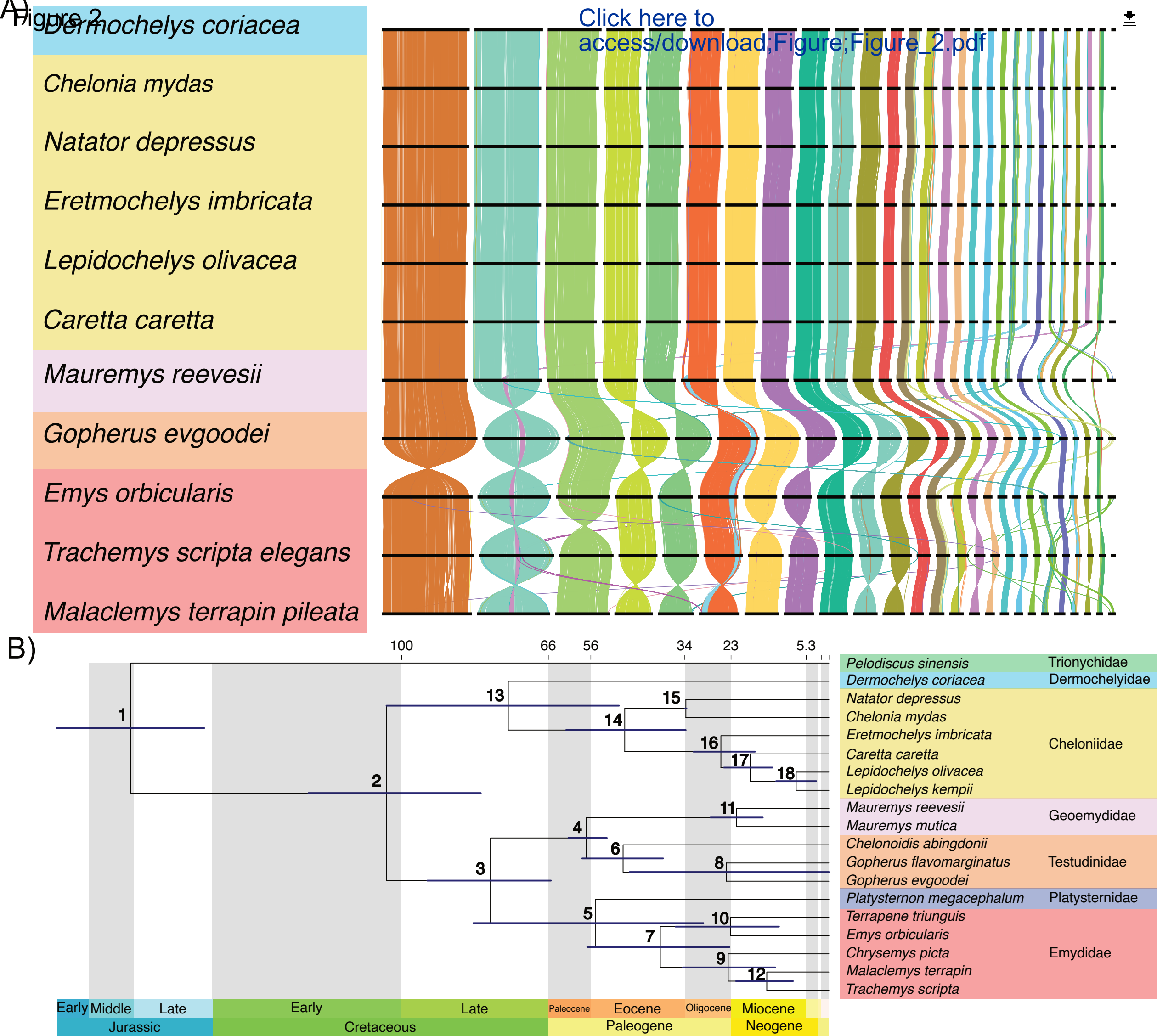

Figure 3

Click here to access/download;Figure;Figure_3.pdf

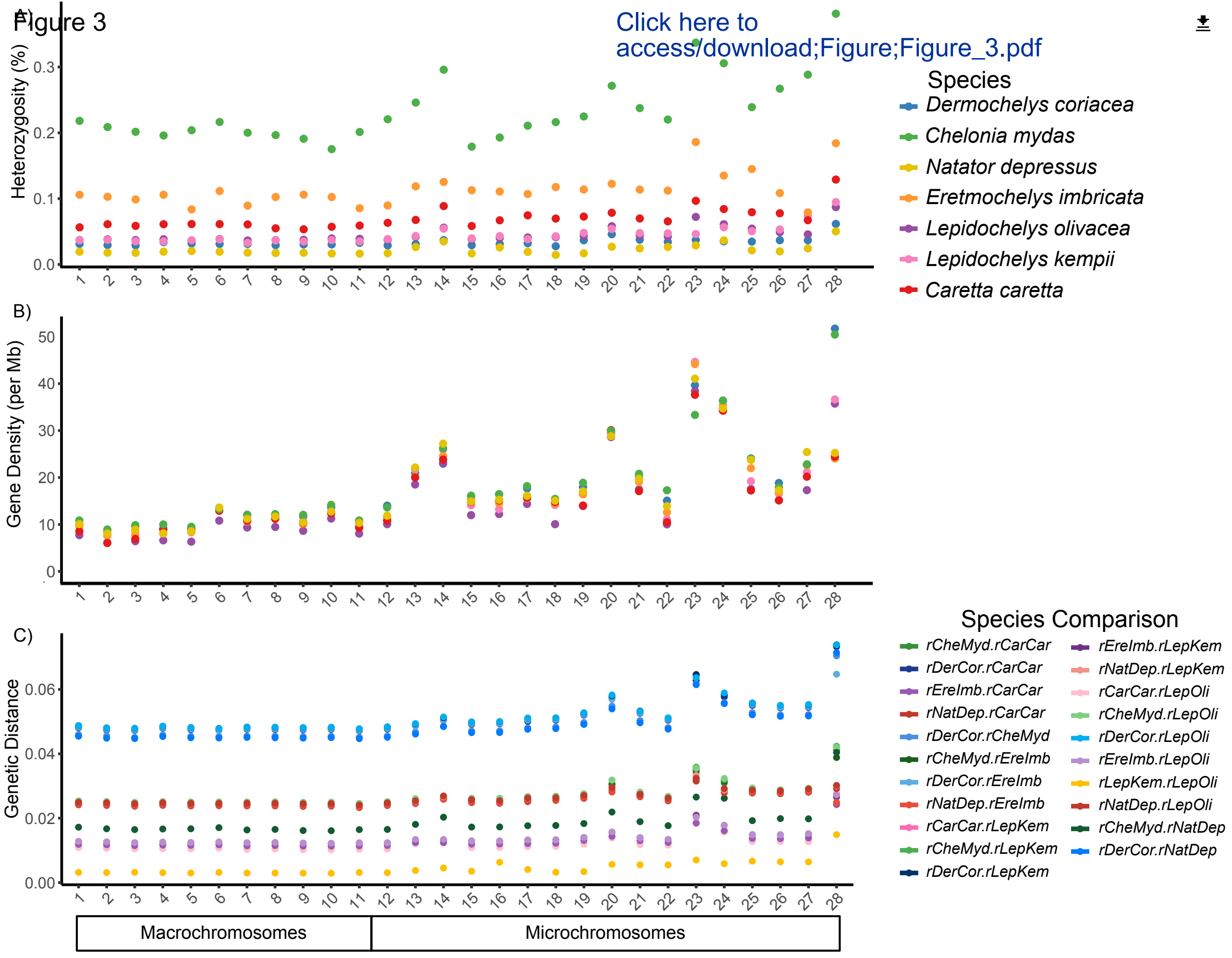

Figure 4

Click here to access/download;Figure;Figure_4.pdf

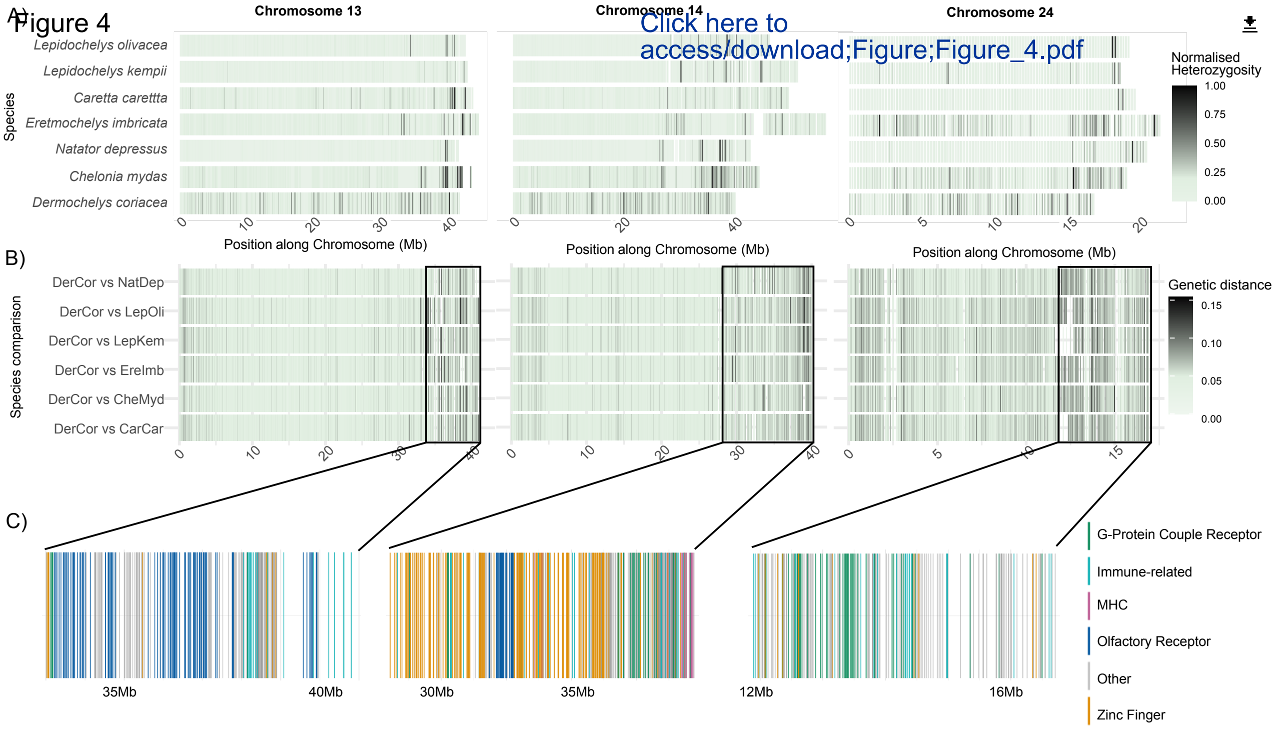

Figure 5

Click here to access/download;Figure;Figure_5 (1).pdf

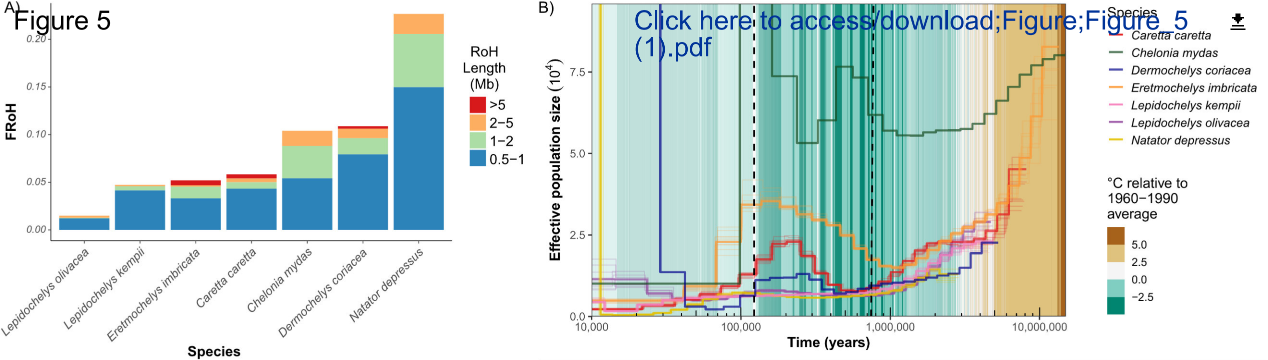

Supplementary Tables S2 and S5

Click here to access/download
Supplementary Material
Supp_Tables_S2_S5.xlsx

Click here to access/download
Supplementary Material
Turtle_SuppMat - Revision 1.pdf

Cover Letter

Click here to access/download;Personal Cover;Cover_Letter.pdf

Institut für Zoo- und Wildtierforschung (IZW)
im Forschungsverbund Berlin e.V.
Alfred-Kowalke-Str. 17
10315 Berlin
tel.: +49 (0)30 5168-0714
web: http://www.izw-berlin.de/

Berlin Center for Genomics in
Biodiversity Research (BeGenDiv)
Königin-Luise-Straße 6-8
14195 Berlin
tel.: +49 (0)30 838 59961
web:http://www.begendiv.de/

Berlin, July 14th 2025

Dear Editors,

We are pleased to submit the revised version of our manuscript **"Haplotype-resolved reference genomes of the sea turtle clade unveil ultra-syntenic genomes with hotspots of divergence"** in light of the comments from the three reviewers.

To address the comments made by the reviewers, we have added much more context to the introduction and discussion section of the manuscript, particularly surrounding the necessity of "reference-quality" genomes as resources for conservation and management practices and a more nuanced interpretation of the results derived from the investigation of homozygous regions of the genome. Furthermore we have validated the findings from PSMC and Darwindow by repeating the analyses using restricted datasets, removing genomic areas likely to cause biases, as well as using complementary tools to those in our initial submission.

During the initial submission we unfortunately did not include Erina J Young as author, who performed sampling alongside Scott D. Whiting. We would greatly appreciate it if we could include Erina as an author to acknowledge the work performed and contributing to the resources. All co-authors are in agreement with this addition.

We hope that with these incorporated changes, you and the reviewers will agree that our manuscript is now ready for publication in Gigascience.

We appreciate your time and consideration and look forward to your feedback. Please do not hesitate to contact us if any additional information is required.

Sincerely,

Dr. Camila Mazzoni (on behalf of all authors)

Evolutionary Genetics Dept, Leibniz Institute for Zoo and Wildlife Research, Alfred-Kowalke-Str. 17, 10315, Berlin, Germany. Tel: +49(30)83859961, email: mazzoni@izw-berlin.de

Reviewer #1:
7. Conclusion
The inferential statistics for the objective "Investigation of multi-copy gene family enrichment in genomic hotspots of sea turtles" were successfully reproduced using the original analysis code provided by the authors. The input data needed to run the code were initially unavailable but were subsequently shared through the Git repository. An inconsistency was noted in the text of the manuscript reporting a threshold for Olfactory Receptors, where the stated 10^-10 should be revised to 10^-9 based on the observed p-value (5.583367e-10).
Thank you for the time taken to thoroughly review our scripts and test their reproducibility. We have updated our human-based rounding error for the Olfactory Receptors in the manuscript (line 234) and incorporated the comments below into both the README file and R script. Particularly, we have made clearer in the README which files or results are used as the input for each script and detailed which Figure was generated using each script.

- Recommendations for authors
While the original analysis code was successfully used to reproduce the results, we recommend improving the documentation to enhance clarity and reproducibility. In particular:
-- Code annotation: The scripts would benefit from more detailed comments within the code to clarify the logic of each step. This would greatly help users follow the analyses more easily and understand the purpose of specific commands or operations.
-- README file: The current README provides only a general overview. We suggest expanding it to include:
--- A brief description of each script or analysis pipeline.
--- An indication of which figure, table, or result in the manuscript each script corresponds to.
--- Clear instructions on how to execute the analyses in the correct order, if applicable.
-- Metadata: For the datasets used or generated by the scripts, it would be helpful to include accompanying metadata files that explain:
--- The definition of each variable name.
--- The origin of each dataset (raw, processed, etc).
--- Any preprocessing steps applied before analysis.
-- Data availability: At this stage, we have only verified the reproducibility of one part of the study. To facilitate full reproducibility of the entire study, we recommend sharing all necessary data files required to run every script present in the repository.

We have incorporated the above comments into the gitlab repository https://git.imp.fu-berlin.de/begendiv/sea_turtlegenomes including the edits made directly by the reviewer. Many thanks again.

These improvements would make the repository significantly more user-friendly and would strengthen the reproducibility of the study.

Reviewer #2: The authors of this work provide a fantastic addition to the genomic resources currently available for marine turtles with five new, apparently high-quality reference genomes. These new resources enable a number of interesting cross-species analyses in this group, including phylogenetic reconstruction, inference of demographic history, and identification of hotspots of diversity and divergence. I though this paper was quite clearly written and easy to read overall, and I have one major and a few more minor comments/suggestions.
We appreciate the reviewer’s positive feedback and thoughtful comments.

Major comment: there is an extensive literature on hybridization among marine turtle lineages (see Vilaca et al. 2021, https://doi.org/10.1111/mec.16113, for a recent genomic example), with lots of evidence for ancient gene flow after initial lineage divergence as well as recent hybridization. The authors do not really mention this phenomenon at all, and since I think it has a lot of bearing on all of the results it would make sense to re-think your findings in light of the fact that some level of gene flow has occurred. Would extensive synteny/lack of genomic rearrangements potentially enable hybridization? Is overall low divergence among lineages potentially a function of gene flow? Are regions of high divergence the result of selection (as you suggest), or could these regions potentially be resistant to gene flow? I believe that IQtree assumes a strictly bifurcating tree, and gene flow can influence PSMC inferences (see Mazet et al. 2016, https://doi.org/10.1038/hdy.2015.104) - how would gene flow among lineages affect your inference of divergence dates and demographic histories?
We thank the reviewer for this insightful comment. Indeed, hybridization has played a significant role in the evolutionary history of sea turtles, with extensive evidence for both ancient gene flow following lineage divergence and ongoing hybridization events. As the reviewer correctly points out, such gene flow can influence divergence time estimates derived from nuclear genomes, often resulting in estimates that appear more recent due to post-divergence gene flow. Despite potential bias, the bifurcating tree model used by MCMCtree provides a widely accepted approximation of the major split events among sea turtle lineages. In response to this suggestion, we have incorporated a comment of this phenomenon into the revised manuscript, specifically in the context of divergence time estimation. We have also added a paragraph speculating how the conservation of gene order may have allowed hybridisation to happen along the evolution of sea turtle lineages. We have added a mitigating statement to this effect in lines 217-221

MInor commentsL [note - line numbers would have been helpful for providing comments on specific items! I will refer to the lower-left page numbers and paragraph instead]:
Apologies for the oversight, we have added line numbers to this submission and referenced the lines in our responses.

page 3, paragraph 2: Some of the applications you refer to here don't seem terribly germane to the relevance of "genomic resources" in management and conservation per se, and several are just methods using some kind of genetic data ... e.g., "abundance"/close-kin mark recapture doesn't require full genomes (and the reference you cite used microsat data), and the "community"/eDNA applications don't generally rely on genomes but instead on databases of a few (usually mitochondrial) genes. Either include methods that truly benefit from the development of high-quality reference genomes or broaden this to something like "growth in molecular ecology techniques".
We appreciate the feedback that we have not highlighted the necessity of highly-accurate, contiguous, chromosome-level assemblies in conservation management, as was also highlighted by review 3 below. We have tried to better highlight how the increased contiguity and scaffolding in particular helps accurate inference of features of the genome architecture itself, such as detecting Runs of Homozygosity, necessary for much of our analysis here. The main strength lies in the “catch-all” nature of these assemblies, allowing not only investigation of the chromosome structure, but also those features possible with fragmented, or draft, assemblies such as gene evolution and overall heterozygosity. We have added sentences to this effect in the introduction at lines 81-91.

page 4, paragraph 2: last sentence is a bit of a run-on, could break this up a bit.
We have split and shortened this sentence in lines 114-115 to hopefully make this section more readable

page 10, paragraph 3: for me, the ROH methods need some additional explanation and interpretation. The more detailed methods indicate that the ROH were identified on the basis of lower-than-average heterozygosity rather than true homozygosity - I can understand why this might have been done (since the baseline level of

heterozygosity varies across species) but it still seems a bit arbitrary and could risk mistaking stretches with simply low variation for IBD tracts. I wonder if a ROH-detection method like ROHan that explicitly incorporates baseline genomic heterozygosity into its model would be more appropriate for comparing results across species and could give different results.

We appreciate the reviewer's comments on ROH detection methods. We chose Darwindow specifically because it can perform the analysis using a single individual. While ROHan would indeed explicitly incorporate baseline heterozygosity, it cannot process long-read data, making it unsuitable for our dataset.
To address concerns about method consistency, we show here a validation using a sea turtle species (*Chelonia mydas*) for which we have both a high-quality genome assembly and short-read data (GCA_015237465.2; SRS18701202). We ran both ROHan and Darwindow on this dataset using comparable parameters (50 Kbp windows) and found similar ROH patterns between the two methods (see below), suggesting that Darwindow's approach of identifying regions with lower-than-average heterozygosity produces comparable results to ROHan's model-based method .

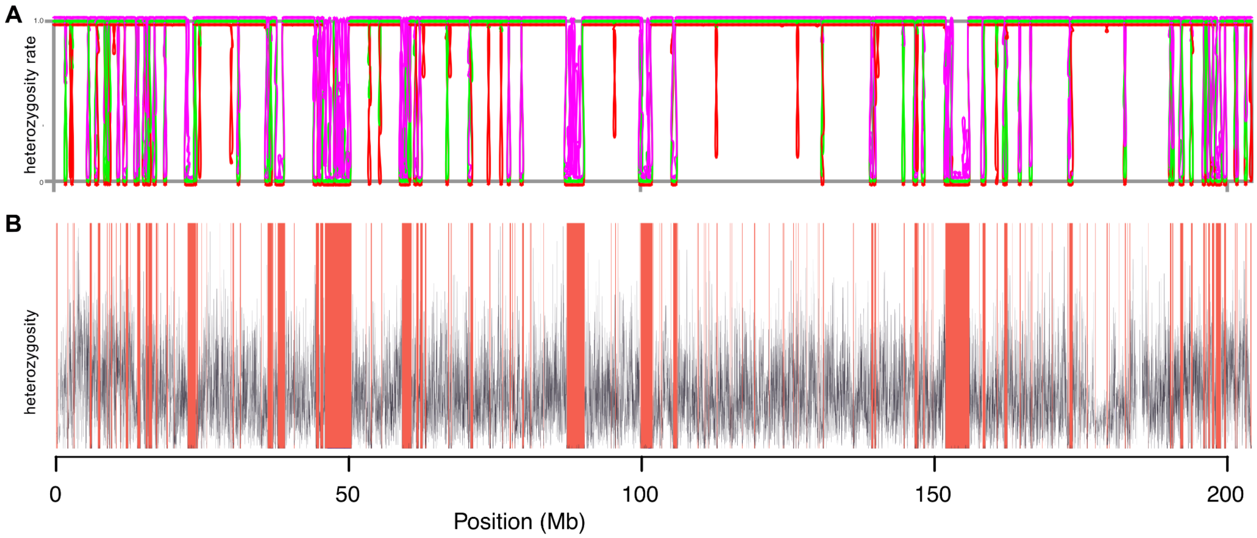

In the above comparative analysis of the *C. mydas* reference genome (GCA_015237465.2), using chromosome 3 (NC_057851.1) as an example, we show that depressions in heterozygosity detected by ROHan generally coincide with ROH segments identified by Darwindow. In panel (A), ROHan results are visualized: green represents point estimates, while magenta and red indicate the upper and lower bounds of local heterozygosity rates, respectively. In panel (B), ROH segments identified by Darwindow are shown as red boxes overlaid on heterozygosity levels (black lines).
Genome-wide, the heterozygosity estimate from ROHan is 0.28% (mid-value), with 12.93% of the genome identified as ROH. For Darwindow, these values are 0.21% and 18.63%, respectively. These results align with those reported by Bentley et al. (2023) for the same individual and dataset, but using PLINK, which estimated a ROH% of 17.7.
To allow a better comparison with the previous PLINK result, ROHan was run with parameters `--size 50000` and `--rohmu 2e-5`. Darwindow was run with `WIN_SIZE="10000"`, `NUM_WIN="5"`, and `MAX_MISS="0.4"`. Bentley et al. (2023) ran PLINK with the `--homozyg` option using the parameters: `--homozyg-kb 50`, `--homozyg-snp 20`, `--homozyg-window-missing 5`, and `--homozyg-window-het 1`.

I also question a bit the interpretation of these low-diversity tracts as evidence of inbreeding per se. The authors do not comment much on the length distributions of these ROH - given that many of them are quite short I would expect that if there was mating between close kin it probably happened
far back in the past and the IBD tracts have been broken up by recombination.
The reviewer makes an important point and is correct that the length distribution of ROH is crucial for distinguishing between ancient demographic processes and recent population restrictions. Although our analysis already included ROH length distributions (Fig 5a), we had not properly incorporated these results into our manuscript. We have now revised the text to better describe the ROH length patterns and provide more nuanced interpretations. We thank the reviewer for this comment as it helps improve our manuscript. We have extensively revised the text in section 3.4 to make it clearer the implications of the lengths of the detected ROHs, lines 254-267

page 11, paragraph 2: for PSMC analyses it is important to note the method assumes that differences in coalescence time/Ne across the genome result from demography alone. If portions of the genome are under balancing/diversifying selection (such as the areas of high diversity that you detect in this study), the local Ne for inferred these regions would be expected to be larger than the rest of the genome, which could lead to the spurious detection of population expansion or contraction (more likely a contraction for balancing selection). See Boitard et al. 2022 (https://doi.org/10.1093/genetics/iyac008) for a more detailed treatment. I would try excluding the regions putatively under diversifying selection and re-run PSMC to see if your inferences change.
We agree that regions under balancing or diversifying selection can bias demographic inferences based on PSMC. To address this concern, we conducted an additional PSMC analysis using data exclusively from the 11 macrochromosomes, excluding the identified high-diversity regions that may be subject to balancing or diversifying selection. The resulting demographic trajectory was highly consistent with our original findings. This likely reflects the fact that many of the high diversity regions are actually repetitive elements that were already masked during the genome preprocessing, and thus excluded from the original PSMC analysis. We have now included this additional analysis and its implications in the revised manuscript, which can be found in Figure S14 (lines 281-286 and 622-625).

Reviewer #3: (1)It is recommended to add keywords such as "conservation genomics" or "adaptive evolution" to better align with the content.
Thank you for your suggestion. We have added both keywords.

(2)In the background section, after discussing the current status of sea turtles and existing genomic research, the study's content is introduced directly without adequately explaining why it is necessary to sequence the genomes of the remaining five species of sea turtles on top of the existing partial genomic data. The introduction of the research objectives appears somewhat abrupt.
Thank you for highlighting our oversight, which was also raised by reviewer 2. As above, we have added sentences to highlight the improved usefulness of highly contiguous, accurate and chromosome-level assemblies for conservation and other management strategies in lines 81-91.

(3)Last line of page four" ……within this ancient clade [34,38]" : When introducing the broad context of genomics and biodiversity conservation, it is important to provide detailed explanations for key concepts such as 'genomic synteny' and 'colinearity'. Although these concepts are covered later in the analysis of the turtle genome, providing initial elaboration can help readers better understand subsequent content.
Thank you for the suggestion. We have added a short definition covering synteny and collinearity to the introduction (Lines 121-123) that we hope will make these concepts clearer to the reader.

(4)Page 6 Section 2.2:The range of this quality value, 38.7, is incorrect. Please verify carefully.
Apologies for the confusion in listing this value. We meant to only highlight the “primary” assemblies and have made this clearer in the text, noting the species from which each value derives and that we restrict this to the primary assembly in each case in lines 163-165.

(5)Result 3.1：High conservation at the chromosomal level is supported, but repetitive sequences must be excluded from synteny analysis.
We agree entirely with this statement and have tried to ensure this is clearer in our methods and results sections. Syntenic comparisons in section 3.1 were performed based on identified protein orthologs, so would not include repetitive sequences. We have added a statement to this effect in section 3.1 and made this clearer in the methods section 6.5. We have also made it clearer in methods section 6.7 that these repetitive regions were also masked before alignments were performed using cactus in order to calculate genetic distances between species.

(6)Section 3.4, Second Paragraph：The reliability of PSMC in low-diversity species, such as N. depressus, may be limited; it is recommended to validate findings with other methods, such as MSMC2.
We agree that validating demographic reconstructions through multiple complementary approaches enhances their robustness. To this end, we applied MSMC2 to the seven sea turtle reference genomes. The resulting effective population size trajectories closely mirrored those inferred from PSMC analyses, providing strong cross-validation of the demographic patterns observed (Figure S15).
While PSMC and MSMC2 relies on the distribution of heterozygous sites across the genome to reconstruct historical effective population size trajectories, to our knowledge, no study has formally evaluated or systematically quantified how reduced heterozygosity in low-diversity species impacts the resolution and reliability of demographic inferences.
To mitigate our demographic analysis limitations, we have taken careful steps to assess the reliability of our PSMC and MSMC2 inferences by (i) evaluating the robustness of their results through bootstrap analyses, and (ii) excluding microchromosomes and regions of high genetic diversity in the 11 macrochromosomes (Figure S14), which are potentially under selection, from the PSMC analysis. Additionally, we have discussed the limitations of generalizing the demographic history of an entire species based on a single genome (Lines 357-363).

(7)It is recommended to include a detailed description of sample selection in the methods section, covering aspects such as geographic distribution, population size, and sample collection methods, to demonstrate the representativeness and reliability of the selected samples.
We have added further details to the manuscript in section 6.1 on the origin of these individuals and the current Regional Management Units responsible in each case.
