# Supplementary material for: Haplotype-resolved reference genomes of the sea turtle clade unveil ultra-syntenic genomes with hotspots of divergence
Source: Gigascience. 2025 Sep 18;14:giaf105. doi: 10.1093/gigascience/giaf105 (PMC12448945; doi:10.1093/gigascience/giaf105)
Supplement: giaf105_Authors_Response_To_Reviewer_Comments_Original_Submission [file giaf105_authors_response_to_reviewer_comments_original_submission.pdf]

Author's Response To Reviewer Comments

Reviewer #1:
7. Conclusion
The inferential statistics for the objective "Investigation of multi-copy gene family enrichment in genomic hotspots of sea turtles" were successfully reproduced using the original analysis code provided by the authors. The input data needed to run the code were initially unavailable but were subsequently shared through the Git repository. An inconsistency was noted in the text of the manuscript reporting a threshold for Olfactory Receptors, where the stated 10^-10 should be revised to 10^-9 based on the observed p-value (5.583367e-10).
Thank you for the time taken to thoroughly review our scripts and test their reproducibility. We have updated our human-based rounding error for the Olfactory Receptors in the manuscript (line 234) and incorporated the comments below into both the README file and R script. Particularly, we have made clearer in the README which files or results are used as the input for each script and detailed which Figure was generated using each script.

- Recommendations for authors
While the original analysis code was successfully used to reproduce the results, we recommend improving the documentation to enhance clarity and reproducibility. In particular:
-- Code annotation: The scripts would benefit from more detailed comments within the code to clarify the logic of each step. This would greatly help users follow the analyses more easily and understand the purpose of specific commands or operations.
-- README file: The current README provides only a general overview. We suggest expanding it to include:
--- A brief description of each script or analysis pipeline.
--- An indication of which figure, table, or result in the manuscript each script corresponds to.
--- Clear instructions on how to execute the analyses in the correct order, if applicable.
-- Metadata: For the datasets used or generated by the scripts, it would be helpful to include accompanying metadata files that explain:
--- The definition of each variable name.
--- The origin of each dataset (raw, processed, etc).
--- Any preprocessing steps applied before analysis.
-- Data availability: At this stage, we have only verified the reproducibility of one part of the study. To facilitate full reproducibility of the entire study, we recommend sharing all necessary data files required to run every script present in the repository.

We have incorporated the above comments into the gitlab repository https://git.imp.fu-berlin.de/begendiv/sea_turtlegenomes including the edits made directly by the reviewer. Many thanks again.

These improvements would make the repository significantly more user-friendly and would strengthen the reproducibility of the study.

Reviewer #2: The authors of this work provide a fantastic addition to the genomic resources currently available for marine turtles with five new, apparently high-quality reference genomes. These new resources enable a number of interesting cross-species analyses in this group, including phylogenetic reconstruction, inference of demographic history, and identification of hotspots of diversity and divergence. I though this paper was quite clearly written and easy to read overall, and I have one major and a few more minor comments/suggestions.
We appreciate the reviewer's positive feedback and thoughtful comments.

Major comment: there is an extensive literature on hybridization among marine turtle lineages (see Vilaca et al. 2021, https://doi.org/10.1111/mec.16113, for a recent genomic example), with lots of evidence for ancient gene flow after initial lineage divergence as well as recent hybridization. The authors do not really mention this phenomenon at all, and since I think it has a lot of bearing on all of the results it would make sense to re-think your findings in light of the fact that some level of gene flow has occurred. Would

extensive synteny/lack of genomic rearrangements potentially enable hybridization? Is overall low divergence among lineages potentially a function of gene flow? Are regions of high divergence the result of selection (as you suggest), or could these regions potentially be resistant to gene flow? I believe that IQtree assumes a strictly bifurcating tree, and gene flow can influence PSMC inferences (see Mazet et al. 2016, https://doi.org/10.1038/hdy.2015.104) - how would gene flow among lineages affect your inference of divergence dates and demographic histories?
We thank the reviewer for this insightful comment. Indeed, hybridization has played a significant role in the evolutionary history of sea turtles, with extensive evidence for both ancient gene flow following lineage divergence and ongoing hybridization events. As the reviewer correctly points out, such gene flow can influence divergence time estimates derived from nuclear genomes, often resulting in estimates that appear more recent due to post-divergence gene flow. Despite potential bias, the bifurcating tree model used by MCMCtree provides a widely accepted approximation of the major split events among sea turtle lineages. In response to this suggestion, we have incorporated a comment of this phenomenon into the revised manuscript, specifically in the context of divergence time estimation. We have also added a paragraph speculating how the conservation of gene order may have allowed hybridisation to happen along the evolution of sea turtle lineages. We have added a mitigating statement to this effect in lines 217-221

MInor commentsL [note - line numbers would have been helpful for providing comments on specific items! I will refer to the lower-left page numbers and paragraph instead]:
Apologies for the oversight, we have added line numbers to this submission and referenced the lines in our responses.

page 3, paragraph 2: Some of the applications you refer to here don't seem terribly germane to the relevance of "genomic resources" in management and conservation per se, and several are just methods using some kind of genetic data ... e.g., "abundance"/close-kin mark recapture doesn't require full genomes (and the reference you cite used microsat data), and the "community"/eDNA applications don't generally rely on genomes but instead on databases of a few (usually mitochondrial) genes. Either include methods that truly benefit from the development of high-quality reference genomes or broaden this to something like "growth in molecular ecology techniques".
We appreciate the feedback that we have not highlighted the necessity of highly-accurate, contiguous, chromosome-level assemblies in conservation management, as was also highlighted by review 3 below. We have tried to better highlight how the increased contiguity and scaffolding in particular helps accurate inference of features of the genome architecture itself, such as detecting Runs of Homozygosity, necessary for much of our analysis here. The main strength lies in the “catch-all” nature of these assemblies, allowing not only investigation of the chromosome structure, but also those features possible with fragmented, or draft, assemblies such as gene evolution and overall heterozygosity. We have added sentences to this effect in the introduction at lines 81-91.

page 4, paragraph 2: last sentence is a bit of a run-on, could break this up a bit.
We have split and shortened this sentence in lines 114-115 to hopefully make this section more readable

page 10, paragraph 3: for me, the ROH methods need some additional explanation and interpretation. The more detailed methods indicate that the ROH were identified on the basis of lower-than-average heterozygosity rather than true homozygosity - I can understand why this might have been done (since the baseline level of heterozygosity varies across species) but it still seems a bit arbitrary and could risk mistaking stretches with simply low variation for IBD tracts. I wonder if a ROH-detection method like ROHan that explicitly incorporates baseline genomic heterozygosity into its model would be more appropriate for comparing results across species and could give different results.

We appreciate the reviewer's comments on ROH detection methods. We chose Darwindow specifically because it can perform the analysis using a single individual. While ROHan would indeed explicitly incorporate baseline heterozygosity, it cannot process long-read data, making it unsuitable for our dataset. To address concerns about method consistency, we show here a validation using a sea turtle species (Chelonia mydas) for which we have both a high-quality genome assembly and short-read data (GCA_015237465.2; SRS18701202). We ran both ROHan and Darwindow on this dataset using comparable parameters (50 Kbp windows) and found similar ROH patterns between the two methods (see below), suggesting that Darwindow's approach of identifying regions with lower-than-average heterozygosity

produces comparable results to ROHan's model-based method .

In the above comparative analysis of the C. mydas reference genome (GCA_015237465.2), using chromosome 3 (NC_057851.1) as an example, we show that depressions in heterozygosity detected by ROHan generally coincide with ROH segments identified by Darwindow. In panel (A), ROHan results are visualized: green represents point estimates, while magenta and red indicate the upper and lower bounds of local heterozygosity rates, respectively. In panel (B), ROH segments identified by Darwindow are shown as red boxes overlaid on heterozygosity levels (black lines).
Genome-wide, the heterozygosity estimate from ROHan is 0.28% (mid-value), with 12.93% of the genome identified as ROH. For Darwindow, these values are 0.21% and 18.63%, respectively. These results align with those reported by Bentley et al. (2023) for the same individual and dataset, but using PLINK, which estimated a ROH% of 17.7.
To allow a better comparison with the previous PLINK result, ROHan was run with parameters --size 50000 and --rohmu 2e-5. Darwindow was run with WIN_SIZE="10000", NUM_WIN="5", and MAX_MISS="0.4". Bentley et al. (2023) ran PLINK with the --homozyg option using the parameters: --homozyg-kb 50, --homozyg-snp 20, --homozyg-window-missing 5, and --homozyg-window-het 1.

I also question a bit the interpretation of these low-diversity tracts as evidence of inbreeding per se. The authors do not comment much on the length distributions of these ROH - given that many of them are quite short I would expect that if there was mating between close kin it probably happened far back in the past and the IBD tracts have been broken up by recombination.
The reviewer makes an important point and is correct that the length distribution of ROH is crucial for distinguishing between ancient demographic processes and recent population restrictions. Although our analysis already included ROH length distributions (Fig 5a), we had not properly incorporated these results into our manuscript. We have now revised the text to better describe the ROH length patterns and provide more nuanced interpretations. We thank the reviewer for this comment as it helps improve our manuscript. We have extensively revised the text in section 3.4 to make it clearer the implications of the lengths of the detected ROHs, lines 254-267

page 11, paragraph 2: for PSMC analyses it is important to note the method assumes that differences in coalescence time/Ne across the genome result from demography alone. If portions of the genome are under balancing/diversifying selection (such as the areas of high diversity that you detect in this study), the local Ne for inferred these regions would be expected to be larger than the rest of the genome, which could lead to the spurious detection of population expansion or contraction (more likely a contraction for balancing selection). See Boitard et al. 2022 (https://doi.org/10.1093/genetics/iyac008) for a more detailed treatment. I would try excluding the regions putatively under diversifying selection and re-run PSMC to see if your inferences change.
We agree that regions under balancing or diversifying selection can bias demographic inferences based on PSMC. To address this concern, we conducted an additional PSMC analysis using data exclusively from the 11 macrochromosomes, excluding the identified high-diversity regions that may be subject to balancing or diversifying selection. The resulting demographic trajectory was highly consistent with our original findings. This likely reflects the fact that many of the high diversity regions are actually repetitive elements that were already masked during the genome preprocessing, and thus excluded from the original PSMC analysis. We have now included this additional analysis and its implications in the revised manuscript, which can be found in Figure S14 (lines 281-286 and 622-625).

Reviewer #3: (1)It is recommended to add keywords such as "conservation genomics" or "adaptive evolution" to better align with the content.
Thank you for your suggestion. We have added both keywords.

(2)In the background section, after discussing the current status of sea turtles and existing genomic research, the study's content is introduced directly without adequately explaining why it is necessary to sequence the genomes of the remaining five species of sea turtles on top of the existing partial genomic data. The introduction of the research objectives appears somewhat abrupt.

Thank you for highlighting our oversight, which was also raised by reviewer 2. As above, we have added sentences to highlight the improved usefulness of highly contiguous, accurate and chromosome-level assemblies for conservation and other management strategies in lines 81-91.

(3)Last line of page four" ……within this ancient clade [34,38]"：When introducing the broad context of genomics and biodiversity conservation, it is important to provide detailed explanations for key concepts such as 'genomic synteny' and 'colinearity'. Although these concepts are covered later in the analysis of the turtle genome, providing initial elaboration can help readers better understand subsequent content.
Thank you for the suggestion. We have added a short definition covering synteny and collinearity to the introduction (Lines 121-123) that we hope will make these concepts clearer to the reader.

(4)Page 6 Section 2.2:The range of this quality value, 38.7, is incorrect. Please verify carefully.
Apologies for the confusion in listing this value. We meant to only highlight the "primary" assemblies and have made this clearer in the text, noting the species from which each value derives and that we restrict this to the primary assembly in each case in lines 163-165.

(5)Result 3.1：High conservation at the chromosomal level is supported, but repetitive sequences must be excluded from synteny analysis.
We agree entirely with this statement and have tried to ensure this is clearer in our methods and results sections. Syntenic comparisons in section 3.1 were performed based on identified protein orthologs, so would not include repetitive sequences. We have added a statement to this effect in section 3.1 and made this clearer in the methods section 6.5. We have also made it clearer in methods section 6.7 that these repetitive regions were also masked before alignments were performed using cactus in order to calculate genetic distances between species.

(6)Section 3.4, Second Paragraph：The reliability of PSMC in low-diversity species, such as N. depressus, may be limited; it is recommended to validate findings with other methods, such as MSMC2.
We agree that validating demographic reconstructions through multiple complementary approaches enhances their robustness. To this end, we applied MSMC2 to the seven sea turtle reference genomes. The resulting effective population size trajectories closely mirrored those inferred from PSMC analyses, providing strong cross-validation of the demographic patterns observed (Figure S15).
While PSMC and MSMC2 relies on the distribution of heterozygous sites across the genome to reconstruct historical effective population size trajectories, to our knowledge, no study has formally evaluated or systematically quantified how reduced heterozygosity in low-diversity species impacts the resolution and reliability of demographic inferences.
To mitigate our demographic analysis limitations, we have taken careful steps to assess the reliability of our PSMC and MSMC2 inferences by (i) evaluating the robustness of their results through bootstrap analyses, and (ii) excluding microchromosomes and regions of high genetic diversity in the 11 macrochromosomes (Figure S14), which are potentially under selection, from the PSMC analysis. Additionally, we have discussed the limitations of generalizing the demographic history of an entire species based on a single genome (Lines 357-363).

(7)It is recommended to include a detailed description of sample selection in the methods section, covering aspects such as geographic distribution, population size, and sample collection methods, to demonstrate the representativeness and reliability of the selected samples.
We have added further details to the manuscript in section 6.1 on the origin of these individuals and the current Regional Management Units responsible in each case.

Cookie Preference Center

We use cookies which are necessary to make our site work. We may also use additional cookies to analyze, improve and personalize our content and your digital experience. For more information, see our Cookie Policy.

You may choose not to allow some types of cookies. However, blocking some types may impact your experience of our site and the services we are able to offer. See the different category headings below to find out more or change your settings.

Allow all

Manage Consent Preferences

Strictly Necessary Cookies

Always active

These cookies are necessary for the website to function and cannot be switched off in our systems. They are usually only set in response to actions made by you which amount to a request for services, such as setting your privacy preferences, logging in or filling in forms. You can set your browser to block or alert you about these cookies, but some parts of the site will not then work.

Functional Cookies

These cookies enable the website to provide enhanced functionality and personalisation. They may be set by us or by third party providers whose services we have added to our pages. If you do not allow these cookies then some or all of these services may not function properly.

Performance Cookies

These cookies allow us to count visits and traffic sources so we can measure and improve the performance of our site. They help us to know which pages are the most and least popular and see how visitors move around the site.

Targeting Cookies

These cookies may be set through our site by our advertising partners. They may be used by those companies to build a profile of your interests and show you relevant adverts on other sites. If you do not allow these cookies, you will experience less targeted advertising.

Cookie List

Clear

Apply Cancel
Consent Leg.Interest

Confirm my choices

Powered by onetrust
