# Supplementary material for: Haplotype-resolved reference genomes of the sea turtle clade unveil ultra-syntenic genomes with hotspots of divergence
Source: Gigascience. 2025 Sep 18;14:giaf105. doi: 10.1093/gigascience/giaf105 (PMC12448945; doi:10.1093/gigascience/giaf105)
Supplement: giaf105_Reviewer_1_Report_Original_Submission — Laura Caquelin -- 4/9/2025 [file giaf105_reviewer_1_report_original_submission.pdf]

Reviewer Report

Title: Haplotype-resolved reference genomes of the sea turtle clade unveil ultra-syntenic genomes with hotspots of divergence

Version: Original Submission **Date:** 4/9/2025

Reviewer name: Laura Caquelin

Reviewer Comments to Author:

Reproducibility report for: Haplotype-resolved reference genomes of the sea turtle clade unveil ultra-syntenic genomes with hotspots of divergence
Journal: GigaScience
ID number/DOI: GIGA-D-25-00103
Reviewer(s): Laura Caquelin, Department of Clinical Neuroscience, Karolinska Institutet, Sweden
----
1. Summary of the Study
The authors aimed to create high-quality reference genomes for five sea turtle species to better understand their genetic diversity, evolutionary adaptations, and ecological traits. They used haplotype-resolved, chromosome-level reference genomes and gene annotations to reveal conserved genome structures, genetic hotspots linked to immune response and sensory evolution, and patterns of demographic expansion. Their findings highlight areas of genetic diversity critical for adaptation and conservation efforts.
----
2. Scope of reproducibility
According to our assessment the primary objective is: Investigation of multi-copy gene family enrichment in genomic hotspots of sea turtles.
- Outcome: Significant enrichment of "MHC", "Immunology-related", "G-Protein Coupled Receptor" (GPCR), "Olfactory Receptor" or "Zinc-Finger" in genomic hotspots with high genetic divergence, diversity, and gene density.
- Analysis method outcome: Fisher's exact test followed by Benjamini-Hochberg correction
- Main result: "Following functional annotation of the genes found in these hotspots, we found enrichment for multi-copy gene families coding for proteins with functions in immune response, olfactory receptors (ORs), zinc fingers, and G-protein-coupled receptors (GPCRs_ (Fig 4c, Tables S6 & S7). This included enrichment of immunology-related genes, GPCRs, ORs, and Zinc-finger genes in chromosome 13 (adjusted p < 10-42, 10-47, 10-79, 0.01, respectively), MHC genes, Immunology-related genes, GPCRs, ORs, and Zinc-finger genes in chromosome 14 (adjusted p < 10-24, 10-6, 10-2, 10-10, 10-52, respectively) and Immunology-related genes and GPCRs in chromosome 24 (adjusted p < 10-3 and 10-3, respectively)." (page 10).
----
3. Availability of Materials
a. Data

- Data availability: Open
- Data completeness: Complete
- Access Method: Repository
- Repository: https://git.imp.fu-berlin.de/begendiv/sea_turtlegenomes
- Data quality: The data files have been shared and appear sufficient for running the analyses. However, no metadata is provided to describe the content, structure, or origin of the files which limits interpretability and reusability.

b. Code
- Code availability: Open
- Programming Language(s): R (for the enrichment test)
- Repository link: https://git.imp.fu-berlin.de/begendiv/sea_turtlegenomes
- License: MIT license
- Repository status: Public
- Documentation: Short README, describe only the presentation of the directory.

----

4. Computational environment of reproduction analysis
- Operating system for reproduction: MacOS 14.7.4
- Programming Language(s): R
- Code implementation approach: Using shared code
- Version environment for reproduction: R version 4.4.1/RStudio 2024.09.0

----

5. Results

5.1 Original study results

Results 1: The main results are presented in Figure 4 and the numerical p-values are available on supplementary table 6 and table 7.

5.3 Steps for reproduction

-> Run the code "enrichment_test.R" shared on Git
- Issue 1: Files needed to run the code are not shared in the Git repository: "GCF_009764565.3_rDerCor1.pri.v4_genomic.longest.aa.tsv", "hotspots_chr13.longest.aa.tsv", "hotspots_chr14.longest.aa.tsv", "hotspots_chr24.longest.aa.tsv".
-- Resolved: These analysis data are not shared in the internal Gigascience FTP server or the Git repository. After request, the authors uploaded all the files into the Git repository.

5.4 Statistical comparison Original vs Reproduced results
- Results: The table S6 and S7 was reproduced:
-- Supplementary table S6: see screenshot from R console
-- Supplementary table S7: see screenshot from R console
- Comments: The original R code "enrichment_test.R" simply stored the p-values results in a value object. To simplify the comparison process, directly obtain the final table, and ensure reproducibility while minimizing errors, we implemented the creation of the table.

------------------ Start of R code ------------------

```
# Creating final tables
## Corresponding to supplementary table S6
```

```
table_S6 <- data.frame(
enrichment = c("MHC", "Immunology", "GPCR", "Olfactory", "Zinc-finger"),
Chr13 = c(p_mhc13, p_immune13, p_gpcr13, p_or13, p_zinc13),
Chr14 = c(p_mhc14, p_immune14, p_gpcr14, p_or14, p_zinc14),
Chr24 = c(p_mhc24, p_immune24, p_gpcr24, p_or24, p_zinc24))
## Corresponding to supplementary table S7
### Create a vector of names for rows and columns ( ! warning the pvalues in fdrs are not in the same order as the table S7)
enrichment <- c("MHC", "Olfactory", "GPCR", "Immunology", "Zinc-finger")
chromosomes <- c("Chr13", "Chr14", "Chr24")
### Reorganizing fdrs in a matrix
table_S7 <- matrix(fdrs, nrow = length(enrichment), byrow = TRUE)
rownames(table_S7) <- enrichment
colnames(table_S7) <- chromosomes
### Organizing rows as the original table S7
library(dplyr)
table_S7 <- as.data.frame(table_S7) # Convert matrix to data frame
table_S7 <- table_S7 %>%
slice(match(c("MHC", "Immunology", "GPCR", "Olfactory", "Zinc-finger"), enrichment))
```

------------------- End of R code -------------------

- Errors detected: The statement "MHC genes, Immunology-related genes, GPCRs, ORs, and Zinc-finger genes in chromosome 14 (adjusted p < 10^-24, 10^-6, 10^-2, 10^-10, 10^-52, respectively)" (page 10) appears to contain an error. Specifically, the p-value for Olfactory Receptors (5.583367e-10) is greater than the threshold of 10^-10, suggesting that this value should instead be below 10^-9. Therefore, the threshold for Olfactory Receptors should be revised to 10^-9.
- Statistical Consistency: The p-values are consistent (see screenshot from R console).

----

7. Conclusion

- Summary of the computational reproducibility review

The inferential statistics for the objective "Investigation of multi-copy gene family enrichment in genomic hotspots of sea turtles" were successfully reproduced using the original analysis code provided by the authors. The input data needed to run the code were initially unavailable but were subsequently shared through the Git repository. An inconsistency was noted in the text of the manuscript reporting a threshold for Olfactory Receptors, where the stated 10^-10 should be revised to 10^-9 based on the observed p-value (5.583367e-10).

- Recommendations for authors

While the original analysis code was successfully used to reproduce the results, we recommend improving the documentation to enhance clarity and reproducibility. In particular:

-- Code annotation: The scripts would benefit from more detailed comments within the code to clarify the logic of each step. This would greatly help users follow the analyses more easily and understand the purpose of specific commands or operations.

-- README file: The current README provides only a general overview. We suggest expanding it to

include:
--- A brief description of each script or analysis pipeline.
--- An indication of which figure, table, or result in the manuscript each script corresponds to.
--- Clear instructions on how to execute the analyses in the correct order, if applicable.
-- Metadata: For the datasets used or generated by the scripts, it would be helpful to include accompanying metadata files that explain:
--- The definition of each variable name.
--- The origin of each dataset (raw, processed, etc).
--- Any preprocessing steps applied before analysis.
-- Data availability: At this stage, we have only verified the reproducibility of one part of the study. To facilitate full reproducibility of the entire study, we recommend sharing all necessary data files required to run every script present in the repository.
These improvements would make the repository significantly more user-friendly and would strengthen the reproducibility of the study.

Methods

Are the methods appropriate to the aims of the study, are they well described, and are necessary controls included? Choose an item.

Conclusions

Are the conclusions adequately supported by the data shown? Choose an item.

Reporting Standards

Does the manuscript adhere to the journal's guidelines on minimum standards of reporting? Choose an item.

Choose an item.

Statistics

Are you able to assess all statistics in the manuscript, including the appropriateness of statistical tests used? Choose an item.

Quality of Written English

Please indicate the quality of language in the manuscript: Choose an item.

Declaration of Competing Interests

Please complete a declaration of competing interests, considering the following questions:

- Have you in the past five years received reimbursements, fees, funding, or salary from an organisation that may in any way gain or lose financially from the publication of this manuscript, either now or in the future?
- Do you hold any stocks or shares in an organisation that may in any way gain or lose financially from the publication of this manuscript, either now or in the future?
- Do you hold or are you currently applying for any patents relating to the content of the manuscript?
- Have you received reimbursements, fees, funding, or salary from an organization that holds or has applied for patents relating to the content of the manuscript?
- Do you have any other financial competing interests?
- Do you have any non-financial competing interests in relation to this paper?

If you can answer no to all of the above, write 'I declare that I have no competing interests' below. If your reply is yes to any, please give details below.

1. No 2. No 3. No 4. No 5. No 6. No

I agree to the open peer review policy of the journal. I understand that my name will be included on my report to the authors and, if the manuscript is accepted for publication, my named report including any attachments I upload will be posted on the website along with the authors' responses. I agree for my report to be made available under an Open Access Creative Commons CC-BY license (http://creativecommons.org/licenses/by/4.0/). I understand that any comments which I do not wish to be included in my named report can be included as confidential comments to the editors, which will not be published.

Choose an item.

To further support our reviewers, we have joined with Publons, where you can gain additional credit to further highlight your hard work (see: https://publons.com/journal/530/gigascience). On publication of this paper, your review will be automatically added to Publons, you can then choose whether or not to claim your Publons credit. I understand this statement.

Yes Choose an item.
