# Supplementary material for: Haplotype-resolved reference genomes of the sea turtle clade unveil ultra-syntenic genomes with hotspots of divergence
Source: Gigascience. 2025 Sep 18;14:giaf105. doi: 10.1093/gigascience/giaf105 (PMC12448945; doi:10.1093/gigascience/giaf105)
Supplement: giaf105_Reviewer_2_Report_Original_Submission — Brendan Reid -- 4/10/2025 [file giaf105_reviewer_2_report_original_submission.pdf]

Reviewer Report

Title: Haplotype-resolved reference genomes of the sea turtle clade unveil ultra-syntenic genomes with hotspots of divergence

Version: Original Submission **Date:** 4/10/2025

Reviewer name: Brendan Reid

Reviewer Comments to Author:

The authors of this work provide a fantastic addition to the genomic resources currently available for marine turtles with five new, apparently high-quality reference genomes. These new resources enable a number of interesting cross-species analyses in this group, including phylogenetic reconstruction, inference of demographic history, and identification of hotspots of diversity and divergence. I though this paper was quite clearly written and easy to read overall, and I have one major and a few more minor comments/suggestions.

Major comment: there is an extensive literature on hybridization among marine turtle lineages (see Vilaca et al. 2021, https://doi.org/10.1111/mec.16113, for a recent genomic example), with lots of evidence for ancient gene flow after initial lineage divergence as well as recent hybridization. The authors do not really mention this phenomenon at all, and since I think it has a lot of bearing on all of the results it would make sense to re-think your findings in light of the fact that some level of gene flow has occurred. Would extensive synteny/lack of genomic rearrangements potentially enable hybridization? Is overall low divergence among lineages potentially a function of gene flow? Are regions of high divergence the result of selection (as you suggest), or could these regions potentially be resistant to gene flow? I believe that IQtree assumes a strictly bifurcating tree, and gene flow can influence PSMC inferences (see Mazet et al. 2016, https://doi.org/10.1038/hdy.2015.104) - how would gene flow among lineages affect your inference of divergence dates and demographic histories?

MInor commentsL [note - line numbers would have been helpful for providing comments on specific items! I will refer to the lower-left page numbers and paragraph instead]:

page 3, paragraph 2: Some of the applications you refer to here don't seem terribly germane to the relevance of "genomic resources" in management and conservation per se, and several are just methods using some kind of genetic data ... e.g., "abundance"/close-kin mark recapture doesn't require full genomes (and the reference you cite used microsat data), and the "community"/eDNA applications don't generally rely on genomes but instead on databases of a few (usually mitochondrial) genes. Either include methods that truly benefit from the development of high-quality reference genomes or broaden this to something like "growth in molecular ecology techniques".

page 4, paragraph 2: last sentence is a bit of a run-on, could break this up a bit.

page 10, paragraph 3: for me, the ROH methods need some additional explanation and interpretation. The more detailed methods indicate that the ROH were identified on the basis of lower-than-average heterozygosity rather than true homozygosity - I can understand why this might have been done (since the baseline level of heterozygosity varies across species) but it still seems a bit arbitrary and could risk mistaking stretches with simply low variation for IBD tracts. I wonder if a ROH-detection method like

ROHan that explicitly incorporates baseline genomic heterozygosity into its model would be more appropriate for comparing results across species and could give different results. I also question a bit the interpretation of these low-diversity tracts as evidence of inbreeding per se. The authors do not comment much on the length distributions of these ROH - given that many of them are quite short I would expect that if there was mating between close kin it probably happened far back in the past and the IBD tracts have been broken up by recombination.
page 11, paragraph 2: for PSMC analyses it is important to note the method assumes that differences in coalescence time/Ne across the genome result from demography alone. If portions of the genome are under balancing/diversifying selection (such as the areas of high diversity that you detect in this study), the local Ne for inferred these regions would be expected to be larger than the rest of the genome, which could lead to the spurious detection of population expansion or contraction (more likely a contraction for balancing selection). See Boitard et al. 2022 (https://doi.org/10.1093/genetics/iyac008) for a more detailed treatement. I would try excluding the regions putatively under diversifying selection and re-run PSMC to see if your inferences change.

Methods

Are the methods appropriate to the aims of the study, are they well described, and are necessary controls included? Choose an item.

Conclusions

Are the conclusions adequately supported by the data shown? Choose an item.

Reporting Standards

Does the manuscript adhere to the journal’s guidelines on [minimum standards of reporting?]() Choose an item.

Choose an item.

Statistics

Are you able to assess all statistics in the manuscript, including the appropriateness of statistical tests used? Choose an item.

Quality of Written English

Please indicate the quality of language in the manuscript: Choose an item.

Declaration of Competing Interests

Please complete a declaration of competing interests, considering the following questions:

- Have you in the past five years received reimbursements, fees, funding, or salary from an organisation that may in any way gain or lose financially from the publication of this manuscript, either now or in the future?
- Do you hold any stocks or shares in an organisation that may in any way gain or lose financially from the publication of this manuscript, either now or in the future?
- Do you hold or are you currently applying for any patents relating to the content of the manuscript?
- Have you received reimbursements, fees, funding, or salary from an organization that holds or has applied for patents relating to the content of the manuscript?
- Do you have any other financial competing interests?
- Do you have any non-financial competing interests in relation to this paper?

If you can answer no to all of the above, write 'I declare that I have no competing interests' below. If your reply is yes to any, please give details below.

I declare that I have no competing interests.

I agree to the open peer review policy of the journal. I understand that my name will be included on my report to the authors and, if the manuscript is accepted for publication, my named report including any attachments I upload will be posted on the website along with the authors' responses. I agree for my report to be made available under an Open Access Creative Commons CC-BY license (http://creativecommons.org/licenses/by/4.0/). I understand that any comments which I do not wish to be included in my named report can be included as confidential comments to the editors, which will not be published.

Choose an item.

To further support our reviewers, we have joined with Publons, where you can gain additional credit to further highlight your hard work (see: https://publons.com/journal/530/gigascience). On publication of this paper, your review will be automatically added to Publons, you can then choose whether or not to claim your Publons credit. I understand this statement.

Yes Choose an item.
