# Supplementary material for: Haplotype-resolved reference genomes of the sea turtle clade unveil ultra-syntenic genomes with hotspots of divergence
Source: Gigascience. 2025 Sep 18;14:giaf105. doi: 10.1093/gigascience/giaf105 (PMC12448945; doi:10.1093/gigascience/giaf105)
Supplement: giaf105_Reviewer_3_Report_Original_Submission — Xiaoli Liu -- 4/21/2025 [file giaf105_reviewer_3_report_original_submission.pdf]

Reviewer Report

Title: Haplotype-resolved reference genomes of the sea turtle clade unveil ultra-syntenic genomes with hotspots of divergence

Version: Original Submission **Date:** 4/21/2025

Reviewer name: Xiaoli Liu

Reviewer Comments to Author:

(1)It is recommended to add keywords such as "conservation genomics" or "adaptive evolution" to better align with the content.
(2)In the background section, after discussing the current status of sea turtles and existing genomic research, the study's content is introduced directly without adequately explaining why it is necessary to sequence the genomes of the remaining five species of sea turtles on top of the existing partial genomic data. The introduction of the research objectives appears somewhat abrupt.
(3)Last line of page four"Previous analyses in particular of the……within this ancient clade [34,38]"ï¼šWhen introducing the broad context of genomics and biodiversity conservation, it is important to provide detailed explanations for key concepts such as 'genomic synteny' and 'colinearity'. Although these concepts are covered later in the analysis of the turtle genome, providing initial elaboration can help readers better understand subsequent content.
(4)Page 6 Section 2.2:The range of this quality value, 38.7, is incorrect. Please verify carefully.
(5)Result 3.1ï¼šHigh conservation at the chromosomal level is supported, but repetitive sequences must be excluded from synteny analysis.
(6)Section 3.4, Second Paragraphï¼šThe reliability of PSMC in low-diversity species, such as N. depressus, may be limited; it is recommended to validate findings with other methods, such as MSMC2.
(7)It is recommended to include a detailed description of sample selection in the methods section, covering aspects such as geographic distribution, population size, and sample collection methods, to demonstrate the representativeness and reliability of the selected samples.

Methods

Are the methods appropriate to the aims of the study, are they well described, and are necessary controls included? Choose an item.

Conclusions

Are the conclusions adequately supported by the data shown? Choose an item.

Reporting Standards

Does the manuscript adhere to the journal's guidelines on minimum standards of reporting? Choose an item.

Choose an item.

Statistics

Are you able to assess all statistics in the manuscript, including the appropriateness of statistical tests used? Choose an item.

Quality of Written English

Please indicate the quality of language in the manuscript: Choose an item.

Declaration of Competing Interests

Please complete a declaration of competing interests, considering the following questions:

- Have you in the past five years received reimbursements, fees, funding, or salary from an organisation that may in any way gain or lose financially from the publication of this manuscript, either now or in the future?
- Do you hold any stocks or shares in an organisation that may in any way gain or lose financially from the publication of this manuscript, either now or in the future?
- Do you hold or are you currently applying for any patents relating to the content of the manuscript?
- Have you received reimbursements, fees, funding, or salary from an organization that holds or has applied for patents relating to the content of the manuscript?
- Do you have any other financial competing interests?
- Do you have any non-financial competing interests in relation to this paper?

If you can answer no to all of the above, write 'I declare that I have no competing interests' below. If your reply is yes to any, please give details below.

I declare that I have no competing interests

I agree to the open peer review policy of the journal. I understand that my name will be included on my report to the authors and, if the manuscript is accepted for publication, my named report including any attachments I upload will be posted on the website along with the authors' responses. I agree for my report to be made available under an Open Access Creative Commons CC-BY license (http://creativecommons.org/licenses/by/4.0/). I understand that any comments which I do not wish to be included in my named report can be included as confidential comments to the editors, which will not be published.

Choose an item.

To further support our reviewers, we have joined with Publons, where you can gain additional credit to further highlight your hard work (see: https://publons.com/journal/530/gigascience). On publication of this paper, your review will be automatically added to Publons, you can then choose whether or not to claim your Publons credit. I understand this statement.

Yes Choose an item.
